# Efficacy and safety of oxfendazole in adults with *Trichuris* spp. infection in Pemba Island, Tanzania: a double-blind, phase 2a, randomised controlled trial

**DOI:** 10.1016/j.eclinm.2026.104273

**Published:** 2026-10-26

**Authors:** Annina Schnoz, Hannah Jeanguenat, Abrahaman S. Msellem, Ibrahim S. Mohammed, Ramadhan S. Abdalla, Salum N. Seif, Amour K. Amour, Mahdi J. Khamis, Fabrice L. Mougeni, Oleksandr Volotko, Olga Sieradzan, Karen Dequatre Cheeseman, George M. Nyangweso, Thaddaeus W. Egondi, Fabiana Barreira, Marc P. Hübner, Sonja Zehetmayer, Said M. Ali, Jennifer Keiser

**Affiliations:** a Department of Research, Swiss Tropical and Public Health Institute, Allschwil, Switzerland; b University of Basel, Basel, Switzerland; c Public Health Laboratory Ivo de Carneri, Chake Chake, Pemba Island, Tanzania; d Center for Medical Data Science, Medical University of Vienna, Vienna, Austria; e Drugs for Neglected Diseases Initiative, Geneva, Switzerland; f University of Bonn, University Hospital Bonn, Institute for Medical Microbiology, Immunology and Parasitology, Bonn, Germany; g German Center for Infection Research, Partner site Bonn-Cologne, Bonn, Germany

**Keywords:** *Trichuris* spp., Trichuriasis, Soil-transmitted helminths, Oxfendazole

## Abstract

**Background:**

Trichuriasis caused by the intestinal parasites *Trichuris trichiura* and *Trichuris incognita,* remains a major public health problem. The widely used benzimidazoles albendazole and mebendazole show low efficacy, highlighting that more effective treatments are needed. One promising candidate is oxfendazole, a highly effective anthelminthic that is widely used in veterinary medicine. Within eWHORM, an adaptive basket trial platform for neglected helminth infections was set up to evaluate the safety and efficacy of oxfendazole against, among others, trichuriasis.

**Methods:**

In this double-blind, phase 2a, randomised, placebo-controlled trial conducted between April 29 and July 16, 2025, on Pemba Island, Tanzania, we evaluated the safety and efficacy of oxfendazole against *Trichuris* spp. infection in adults aged 18–65 years. A total of 127 participants were randomised in a 1:1:1:1 ratio to receive either placebo on three consecutive days, 400 mg oxfendazole on day 1 followed by placebo on two consecutive days, 800 mg oxfendazole on day 1 followed by placebo on two consecutive days or 800 mg oxfendazole on three consecutive days. The primary outcome was *Trichuris* spp. egg count at day 18, expressed as eggs per gram of stool based on quadruplicate Kato-Katz thick smears. Secondary outcomes included cure rates and egg reduction rates for *Trichuris* spp., *Ascaris lumbricoides*, and hookworm. Adverse events were recorded 3 h, 24 h, and 18 days post-treatment. The trial is registered at the Pan African Clinical Trials Registry (PACTR202412611774752).

**Findings:**

Between April 29 and May 21, 2025, 229 participants were screened for *Trichuris* spp. infection, of whom 127 were treated. Of these, 31 received 3 × 800 mg oxfendazole, 34 received 1 × 800 mg oxfendazole, 30 received 1 × 400 mg oxfendazole and 32 received placebo. The egg count at day 18 was 1099.2 (SD 3354.6) for placebo, 218.3 (SD 506.4) for 1 × 400 mg oxfendazole, 148.4 (SD 244.7) for 1 × 800 mg oxfendazole and 36.2 (SD 117.5) for 3 × 800 mg oxfendazole. Cure rate for 3 × 800 mg oxfendazole was 75.9% (95% CI 57.9–87.8). Single oxfendazole doses revealed lower cure rates of 31.0% (95% CI 17.3–49.2) at 400 mg and 35.3% (95% CI 21.5–52.1) at 800 mg. The placebo group had a cure rate of 20.0% (95% CI 9.5–37.3). All doses were well tolerated and showed a similar number of adverse events compared to placebo. The most common adverse event was abdominal pain.

**Interpretation:**

Single doses of oxfendazole showed no benefit over the cure rates reported in the literature for the currently used drugs albendazole and mebendazole against *Trichuris* spp. infections. However, given its high efficacy when used at multiple doses, the drug could play a role in targeted treatment schemes. Moreover, multiple-dose administration of oxfendazole may confer an ancillary public health benefit against *Trichuris* spp. when used to treat helminth infections requiring multi-day regimens, particularly in co-endemic regions.

**Funding:**

EDCTP3 Joint Undertaking and the Swiss State Secretariat for Education, Research and Innovation (SERI).

**Research in context**

**Evidence before this study**

We searched PubMed, Google Scholar and ClinicalTrials.gov from database inception until June 17, 2026, using the search terms “oxfendazole” AND (“trichuriasis” OR “trichuris” OR “soil-transmitted helminths” OR “human”) AND “trial” without language restrictions. Besides numerous studies in animals, we identified three phase 1 trials conducted between 2014 and 2022 exploring the pharmacokinetics, tolerability and safety of oxfendazole in healthy human volunteers. These studies evaluated both single- and multiple-dose regimens of oxfendazole administered as oral suspension or tablet formulations. In the single ascending dose trial (NCT02234570), oral doses ranging from 0.5 to 60 mg/kg body weight were investigated. In the multiple ascending dose trial (NCT03035760), participants received oral suspension doses of 3, 7.5, and 15 mg/kg once daily for 5 days. In the bioequivalence trial (NCT04920292), a field applicable immediate-release formulation was evaluated at single doses of 100 mg and 400 mg, as well as 400 mg once daily for 5 consecutive days. Across these studies, oxfendazole was well tolerated, with no major safety concerns reported and pharmacokinetic analyses consistently demonstrated a long elimination half-life. We also identified an ongoing phase 2 trial in Peru evaluating single dose oxfendazole at 400 mg and 800 mg provided as study drug capsules in adults infected with *Trichuris trichiura* and comparing its efficacy and safety with those of a single 400 mg dose of albendazole, but results have not yet been reported.

**Added value of this study**

This is the first study to report the efficacy and safety of oxfendazole administered as a field applicable tablet in *Trichuris* spp.-infected individuals. Our study shows that oxfendazole was well tolerated. We observed high egg reduction rates following single-dose (1 × 400 mg and 1 × 800 mg) oxfendazole and high cure rates in the multiple-dose treatment arm (3 × 800 mg).

**Implications of all the available evidence**

Soil-transmitted helminths affect more than 1.5 billion people worldwide and remain a major public health problem. Despite ongoing control efforts, *Trichuris* spp. infections remain difficult to treat effectively, highlighting the need for improved treatment options. Achieving the WHO goal of eliminating soil-transmitted helminthiasis as a public health problem by 2030, defined as fewer than 2% of at-risk individuals harbouring moderate or heavy intensity infections, will require more effective medicines. In this study, single-dose oxfendazole showed low cure rates but high egg reduction rates against *Trichuris* spp., whereas multiple-dose administration achieved high cure and egg reduction rates. These findings suggest that oxfendazole may have a role in targeted treatment strategies for trichuriasis. Moreover, multiple dose oxfendazole could provide an additional public health benefit if proven effective for the treatment of other neglected helminth infections requiring multi-day regimens in co-endemic settings.

## Introduction

Soil-transmitted helminths remain a major public health problem infecting over 1.5 billion people globally.^1^ These intestinal parasites include *Trichuristrichiura, Trichuris* *incognita*, *Ascaris lumbricoides, Strongyloides stercoralis,* and the hookworms *Necator americanus, Ancylostoma duodenale* and *Ancylostoma ceylanicum*. People at risk live in tropical and subtropical areas with poor sanitation and hygiene, and limited access to clean water.^1^, ^2^, ^3^, ^4^ The World Health Organization (WHO) recommends preventive chemotherapy as a core strategy to control soil-transmitted helminth morbidity.^5^ The benzimidazoles albendazole and mebendazole, which are widely used in such control programmes, are highly effective as single-dose treatments against *A. lumbricoides*, achieving cure rates of 95.7% and 96.2%, respectively. For hookworm infections, albendazole is substantially more effective than mebendazole, with cure rates of 79.5% and 32.5%, respectively. In contrast, both drugs show limited efficacy against *Trichuris* spp., with cure rates of only 30.7% for albendazole and 42.1% for mebendazole.^6^ If left untreated, repeated *Trichuris* spp. infections can cause severe symptoms that contribute to malnutrition and impair both physical and cognitive development in children.^2^ To reach the WHO goal to eliminate soil-transmitted helminthiasis as a public health problem by 2030, defined as fewer than 2% of the at-risk population having moderate or heavy infection, more effective medicines must be developed.^6^, ^7^

One drug of interest is oxfendazole, a benzimidazole that degenerates parasite microtubules, blocking glucose uptake and depleting its energy reserves, ultimately leading to parasite death, while having no effect on glucose metabolism in mammals.^8^ Oxfendazole is a broad-spectrum anthelminthic with a long metabolic half-life and high efficacy against intestinal parasites in animals.^9^ Since its approval in 1990, it has been used for treating cestodes and nematodes in dogs, horses, and cattle.^9^, ^10^, ^11^, ^12^

In 2014–2015, a first-in-human study was conducted, investigating the pharmacokinetics, safety, and tolerability of oxfendazole in liquid formulation (a single 0.5–60 mg/kg dose and placebo) in 70 volunteers. Oxfendazole was rapidly absorbed and showed a plasma half-life of 8.5–11 h. There were no significant differences found in haematology, biochemistry, or urine parameters between participants receiving oxfendazole and those receiving placebo. No serious adverse events or deaths were reported.^11^ In 2017–2019, an open label, multiple ascending dose phase 1 study including 36 participants demonstrated the safe use of multiple-dose administration of oxfendazole (3–15 mg/kg once daily for 5 days), an important step for advancing treatment of nematode infections that are more difficult to cure with a single-dose.^10^ In 2022, another phase 1 study was conducted in Tanzania involving 30 healthy adults. Participants received a newly developed tablet formulation, which was also used in the present study, administered either as a single-dose (100 or 400 mg) or as a multiple-dose regimen (400 mg daily for five consecutive days), alongside a placebo group. Oxfendazole was well tolerated, with no adverse events or clinically significant abnormalities observed.^13^ The results of these studies supported moving oxfendazole forward into phase 2 clinical studies.

Within the EU funded eWHORM (enabling the WHO Road Map 2030) consortium, an adaptive clinical trial platform using a basket design was set up, which enabled the implementation of a unified protocol across four sub-Saharan African countries, each sub-study evaluating the safety and efficacy of oxfendazole against one or more helminthiases: onchocerciasis, loiasis, mansonellosis and/or trichuriasis.^14^

In this double-blind, randomised, controlled, phase 2a trial, we aimed to determine the efficacy and safety of oxfendazole in adult participants with *Trichuris* spp. infections on Pemba Island, Tanzania. In addition, we assessed the efficacy of oxfendazole in co-infections with *A. lumbricoides* and hookworm. Participants were randomised to receive either placebo on three consecutive days, 400 mg oxfendazole on the first day followed by placebo on two consecutive days, 800 mg oxfendazole on the first day followed by placebo on two consecutive days or 800 mg oxfendazole on three consecutive days.

## Methods

### Study design

This double-blind, randomised, placebo-controlled phase 2a trial was conducted from April 29 to July 16, 2025 in two villages in the shehia (the smallest official administrative unit in Zanzibar) of Ndagoni on Pemba Island, Tanzania.

### Ethics

The trial was managed in accordance with the Declaration of Helsinki and International Council of Harmonization Guidelines E6(R2) Good Clinical Practice, which were in effect at the time of trial conduct.^15^, ^16^ Written informed consent was obtained from all participants prior to enrolment in the study. For participants who were illiterate, the consent information was provided and explained verbally in the local language, and informed consent was documented by thumbprint in the presence of an impartial witness.

Ethical approvals were obtained from Zanzibar Health Research and Ethical Committee (reference number ZAHREC/01/PR/FEB/2025/09) and the Ethics Committee of Northwestern and Central Switzerland (reference number AO_2025-00012). Regulatory approval was granted by the Zanzibar Food & Drug Administration (reference number JE.5/14/01/17). This trial was registered at the Pan African Clinical Trials Registry (PACTR202412611774752) on February 28, 2025.

### Participants

Consented participants aged 18–65 years testing positive for *Trichuris* spp. in at least two out of four Kato-Katz thick smears were invited for a clinical baseline assessment. Women of childbearing potential (WOCBP) were eligible if they agreed to use a highly effective, culturally acceptable contraceptive method from before the first investigational medicinal product (IMP) dose until 33 days after the final dose. Male participants were eligible if they agreed to use a condom during vaginal intercourse with partners of childbearing potential from the first IMP dose until 33 days after the final dose. Other factors that led to non-eligibility for the trial included: body weight was <45 kg, current or chronic history of liver disease, pregnancy and/or breastfeeding, haemoglobin levels <10 g/dL at baseline, aspartate aminotransferase and/or alanine aminotransferase and/or total bilirubin >1.5× upper limit of normal range at baseline, estimated Glomerular Filtration Rate <60 ml/min at baseline,^17^ more than one epileptic seizure within the past 5 years, acute and/or febrile illness requiring therapy within 14 days prior to baseline, *Paragonimus* sp. eggs or *Taenia* sp. proglottids or eggs in stool, anthelminthic therapy within one month prior baseline, concomitant use of medications contraindicated with benzimidazoles, known hypersensitivity to benzimidazoles or any component of the oxfendazole formulation, current or planned exposure to another IMP, or participation in an interventional clinical study within 3 months prior to screening or within five drug half-lives of the investigational product administered in the previous study, whichever was longer and/or any condition, in the investigator’s opinion, that placed the participant at undue risk.

Sex was reported by the participants with no pre-specified options and stored as a binary variable based on the received responses. Race was not recorded as a demographic variable, because the study was conducted in the Ndagoni shehia of Pemba Island, where the population is racially homogenous.

### Randomisation and masking

Randomisation was performed through a web-based randomisation system called Randomizer.^18^ An independent unblinded statistician was responsible for setting up the randomisation system. Varying block sizes of 8, 12 and 16 were used. The site investigator executed the randomisations after confirmed eligibility by entering the participant’s identification number. The randomisation system issued the ‘Treatment Code’, which indicated the assigned blinded treatment box to which the participant was allocated. Participants were randomised 1:1:1:1 to receive either placebo on three consecutive days, 400 mg oxfendazole on the first day followed by placebo on two consecutive days, 800 mg oxfendazole on the first day followed by placebo on two consecutive days or 800 mg oxfendazole on three consecutive days. The placebo treatment was designed to match the active treatment in appearance, weight, and presentation to ensure maintenance of blinding. The IMP, including the placebo, was manufactured at the Good Manufacturing Practice (GMP) compliant facility of Syngene International Ltd., India. Blinded labelling was subsequently performed under GMP conditions at Bilcare Ltd., India. The final product was then exported to Pemba Island, Tanzania under temperature-controlled conditions in compliance with Good Distribution Practice.

This study was double blinded, all investigators, study team members, physicians, outcome assessor, and participants were masked to treatment allocation and did not know which treatment participants received.

### Procedures

We recruited adults from the community and invited them to participate in informational sessions in their village. Local study personnel informed individuals about the trial objectives including study procedures, potential benefits and risks, and what participation would entail, emphasizing that involvement was voluntary and withdrawal from the study was possible at any time without consequences. Potential participants had the opportunity to ask questions. Upon providing written informed consent, their name and demographics were registered. Illiterate participants consented with a thumb print in presence of an independent witness. Birth certificates were reviewed when a participant’s reported age was close to the age inclusion limits. Consented participants were asked to provide two morning stool samples, if possible, on two consecutive days. Stool samples were transported in cool boxes to the Public Health Laboratory-Ivo de Carneri. There, from each sample, duplicate Kato-Katz thick smears (each replicate 41.7 mg of stool) were prepared and examined by experienced technicians for the presence of eggs of *Trichuris* spp. as well as *A. lumbricoides* and hookworm via bright-field microscopy. For quality control of *Trichuris* spp. and *A. lumbricoides* egg counts, 10% of slides from each day were randomly selected and independently re-examined by a second reader the following day. If quality control reading exceeded defined limits of no difference in presence of parasite eggs between the two readings, ±10 egg counts for values ≤ 100 eggs or ±20% for values > 100 eggs, a third reading was performed by another independent lab technician. The result closest to the third reading was chosen as the accepted result.^19^

*Trichuris* spp. positive participants were assessed for eligibility during a baseline visit conducted within one week prior to the IMP administration. During this visit, participants’ medical history and concomitant medications were recorded and height, weight, and vital signs (including respiratory rate, pulse rate, temporal body temperature, and blood pressure) were measured. Physical and neurological examinations were conducted by study physicians using a standardized physical examination checklist and neurological questioning and reflex testing. The physicians informed participants about concomitant medications prohibited during the trial period (Supplementary Table S1). For WOCBP, the first day of the last menstrual period was recorded. Venous blood (maximum of 2 × 4 ml) and urine (10–20 ml) samples were collected at the village health centre. Blood samples for clinical chemistry were centrifuged before transport. Blood and urine samples were then placed in cool boxes and transported to the laboratory for analysis. Clinical chemistry and haematology were conducted at the Chake Chake Health Centre Laboratory while urinalysis was performed at the Public Health Laboratory-Ivo de Carneri within the same day.

Participants meeting the aforementioned criteria assessed were enrolled into the study and were invited to attend the treatment days scheduled from May 29 to May 31, 2025. Participants were requested to fast for 8 h prior to IMP administration and were provided with breakfast 2 h after IMP administration. Before IMP administration, vital signs and baseline symptoms were recorded and WOCBP were asked for a urine sample to assess pregnancy status. Upon confirmation of eligibility, participants were randomised and received eight tablets with water under observation. Each tablet contained either 100 mg oxfendazole or matching placebo. Participants remained under observation for 3 h following treatment, after which adverse events were recorded with general, non-leading questions, vital signs were measured, and lunch was provided. Before the next IMP dose was administered and 24 h after the last dose, participants were asked for any symptoms experienced since the 3 h follow-up on the previous day. Adverse events were assessed for severity based on the National Cancer Institute Common Terminology Criteria for Adverse Events, version 5.0.^20^ At 18 days (±3 days) after IMP administration, two stool samples were collected for parasitological diagnosis as described above. Venous blood and urine samples were collected and analysed as described above and results reviewed by the study physicians. Any clinically significant laboratory abnormalities that occurred after administration of the study treatment were recorded as adverse events, if applicable. For WOCBP, a follow-up pregnancy test was performed and the first day of the last menstrual period was recorded. Adverse events and concomitant medications taken since IMP administration were recorded. Physical and neurological examinations were performed and vital signs were measured. WOCBP attended the day 47 follow-up for a pregnancy test, and any adverse events were recorded. Participants who tested positive for any soil-transmitted helminth infection at follow-up, as well as excluded individuals, received oral albendazole (400 mg daily for three days).

### Outcomes

The primary outcome was egg count at day 18, defined as eggs per gram of stool (EPG) calculated for each participant as mean *Trichuris* spp. egg count in quadruplicate Kato-Katz thick smears multiplied by 24 collected 18 days after treatment among participants who were infected at baseline. Secondary outcomes included cure rate and egg reduction rate for *Trichuris* spp., as well as cure rates and egg reduction rates for *A. lumbricoides* and hookworm. Safety outcomes included the frequency and severity of adverse events as well as differences in vital signs, physical and neurological examination, clinical chemistry, haematology and urinalysis.

### Statistical analysis

An overall sample size of N = 160 (resulting in 40 participants per dose) and a drop-out rate of 10% was calculated, power to reject any active dose compared to placebo exceeds 90% across several considered scenarios where at least one active dose has a reduction rate of 60% (Bonferroni-Holm adjustment for the three comparisons with the Wilcoxon test, one-sided, alpha = 0.025). Source data were recorded on paper and subsequently entered into the Medrio electronic data capture (EDC) system (Medrio, Inc., San Francisco, CA, USA), a validated, regulatory-compliant platform. Data management activities, including query generation and resolution, were conducted within the EDC system to ensure data quality and integrity.

For the primary analysis the modified intention to treat (mITT) population (participants who received at least one intake of IMP and for which the primary endpoint was observed) was included which was identical to the per protocol (PP) population (participants in the mITT population who were free from major protocol violations). The primary objective of the study is to identify a safe and efficient dose of oxfendazole for participants infected with *Trichuris* spp.

Cure rates and geometric and arithmetic egg reduction rates were calculated at day 18 post-treatment for *Trichuris* spp. and *A. lumbricoides* and hookworm co-infections. For the comparison of egg counts and reduction rates of placebo group to active doses, one-sided Wilcoxon tests were calculated. Cure rates were compared with Chi-square tests. For each, Bonferroni-Holm adjustment for three comparisons at level alpha = 0.025 was performed. In addition, a post hoc analysis was conducted to calculate cure rates and egg reduction rates according to baseline infection intensity, categorized as light or moderate-to-heavy based on WHO criteria.^21^^,^^22^

$${ERR}_{GM}=\left(1-\frac{e^{\frac{1}{n}\sum \log\left({EPG}_{post\;-\;treatment}\;+\;1\right)}-1}{e^{\frac{1}{n}\sum \log\left({EPG}_{pre\;-\;treatment}\;+\;1\right)}-1}\right)\;\times \;100$$

$${ERR}_{AM}=\left(1-\frac{\frac{1}{n}\sum {EPG}_{post\;-\;treatment}}{\frac{1}{n}\sum {EPG}_{pre\;-\;treatment}}\right)\;\times \;100$$

As a sensitivity analysis, missing primary endpoints were handled using multiple imputation with the mice package in R.^23^ For each comparison, missing outcome values were imputed using a zero-inflated Poisson model including dose group, sex, age, and baseline egg count as predictors in both the zero and count components of the imputation model (20 repetitions). In each completed dataset, the probabilistic index was estimated using the Wilcoxon approach. The resulting probabilistic index estimates were pooled on the logit scale using Rubin’s rules, with confidence intervals back-transformed to the probabilistic index scale. Ties were retained in the Wilcoxon analysis. The sensitivity analysis using multiple imputation yielded results consistent with the primary analysis, supporting the robustness of the findings (Supplementary Table S2).

The safety analysis set comprised all randomised participants who received at least one dose of study treatment. Descriptive summaries of vital signs, clinical chemistry, haematology, and urinalysis were provided at baseline and day 18, while physical and neurological examination findings were summarized as the number and percentage of normal and abnormal findings. An independent Data and Safety Monitoring Board was established to monitor safety data.

Statistical analysis of all data was performed using R version 4.5.1.

### Role of the funding source

The funders of this study had no role in study design, data collection and analysis, decision to publish, or preparation of the manuscript.

## Results

From April 29 to May 21, 2025, 229 individuals were screened for *Trichuris* spp. infection of which five refused to provide stool samples, three were below 18 years, and 184 were positive (Fig. 1). Of the 184 individuals, 11 were on long-term travel, 10 refused participation, and 34 were excluded due to exclusion criteria including low body weight, increased liver enzyme values, use of concomitant or anthelminthic treatment, low haemoglobin levels, and pregnancy. The remaining 129 participants were invited for treatment, two of whom were absent at treatment days. 127 participants were randomised, 31 were assigned to 800 mg oxfendazole administered on three consecutive days, 34 to 1 × 800 mg oxfendazole, 30 to 1 × 400 mg oxfendazole and 32 to placebo. Five participants did not provide follow-up stool samples, two of whom were allocated to 3 × 800 mg oxfendazole, two to placebo, and one to 1 × 400 mg oxfendazole. Including four participants who did not provide follow-up stool samples, a total of 13 participants did not present themselves for the safety assessment on day 18 and of those, two WOCBP also missed the day 47 visit. Participants missing safety assessments at follow-up but providing stool samples were included in the mITT population which was identical to the PP population.

**Fig. 1:**
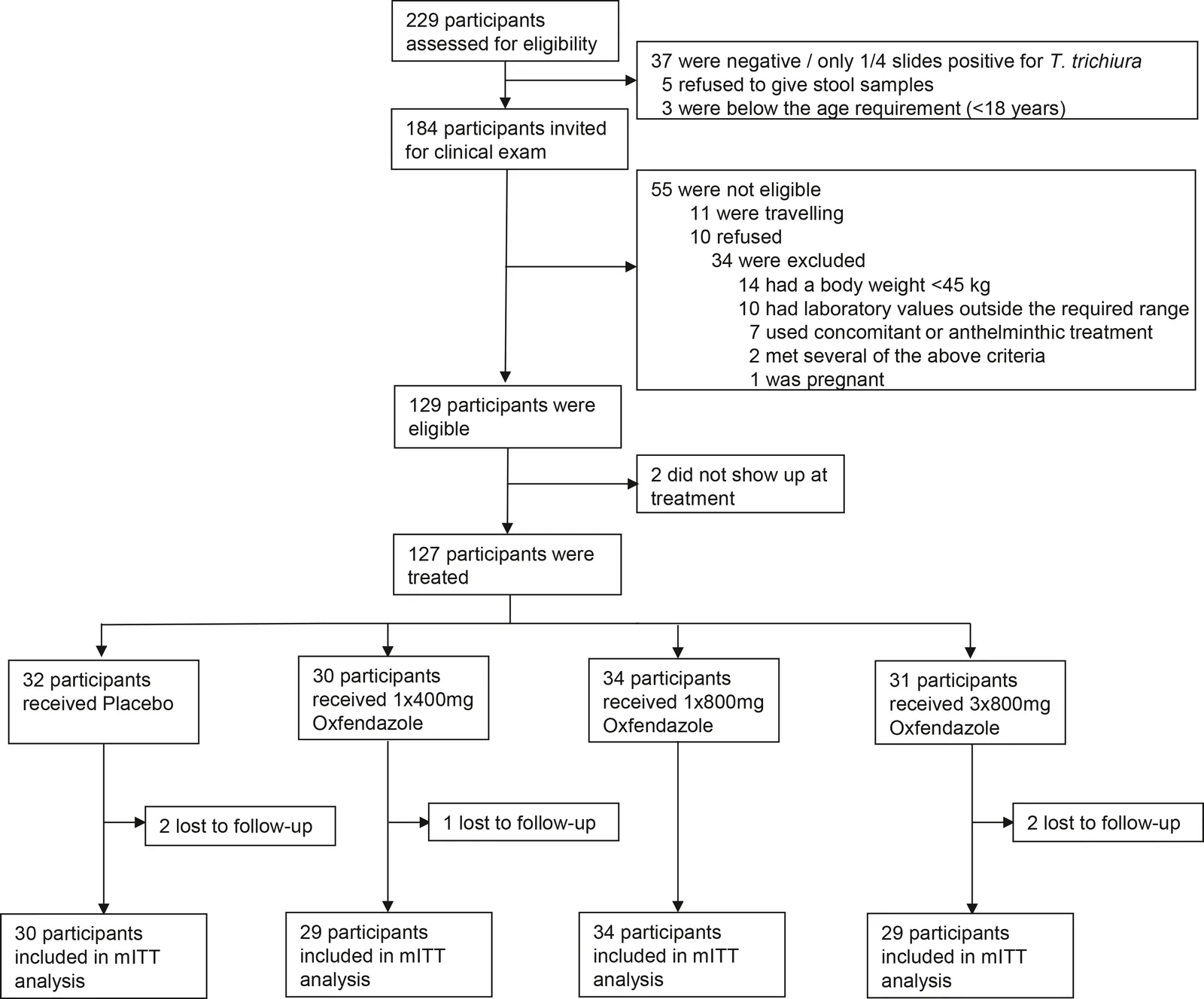
Trial profile.

Baseline demographics and parasitological characteristics of randomised participants are shown in Table 1. There were more female participants (66.1%, 84/127) treated compared to male participants (33.9%, 43/127). The proportion of female participants in the different study arms ranged from 50.0% to 79.4%. The mean age across the different treatment arms ranged from 35.5 to 39.4 years. The distributions of body weight and height, as well as the number of co-infections, were comparable across the treatment arms. The intensity of *Trichuris* spp. infection varied among treatment groups, with 71.0%–88.2% being lightly infected. The baseline mean EPG was substantially higher in the 3 × 800 mg oxfendazole arm with a geometric mean EPG of 527.6 compared to the other arms ranging from 207.7 to 337.0. A baseline adjusted sensitivity analysis of the primary endpoint is shown in Supplementary Table S3.

**Table 1:** Baseline characteristics of randomised study participants (ITT population = all randomised participants).

	Placebo (N = 32)	1 × 400 mg OXF (N = 30)	1 × 800 mg OXF (N = 34)	3 × 800 mg OXF (N = 31)
Sex				
Female	22 (68.8%)	15 (50.0%)	27 (79.4%)	20 (64.5%)
Male	10 (31.2%)	15 (50.0%)	7 (20.6%)	11 (35.5%)
Age (years)	36.1 (14.1)	35.5 (12.8)	38.1 (13.3)	39.4 (15.1)
Weight (kg)	59.6 (10.3)	60.9 (9.8)	58.5 (8.0)	63.8 (14.3)
Height (cm)	163.0 (8.8)	163.5 (8.6)	161.9 (7.3)	163.5 (8.4)
*Trichuris* spp. infection intensity				
Arithmetic mean EPG	932.3 (2120.3)	1247.0 (2525.5)	413.8 (502.7)	1524.8 (3126.6)
Geometric mean EPG	207.7 (5.4)	337.0 (5.4)	223.4 (3.3)	527.6 (4.1)
Light (<1000 EPG)	27 (84.4%)	24 (80.0%)	30 (88.2%)	22 (71.0%)
Moderate/High (≥1000 EPG)	5 (15.6%)	6 (20.0%)	4 (11.8%)	9 (29.0%)
Co-infection status				
*Ascaris lumbricoides*	6 (18.8%)	10 (33.3%)	8 (23.5%)	7 (22.6%)
Light (<5000 EPG)	4 (66.7%)	6 (60.0%)	6 (75.0%)	3 (42.9%)
Moderate/High (≥5000 EPG)	2 (33.3%)	4 (40.0%)	2 (25.0%)	4 (57.1%)
Hookworm	10 (31.2%)	12 (40.0%)	9 (26.5%)	12 (38.7%)
Light (<2000 EPG)	10 (100.0%)	12 (100.0%)	9 (100.0%)	11 (91.7%)
Moderate/High (≥2000 EPG)	0 (0.0%)	0 (0.0%)	0 (0.0%)	1 (8.3%)
*Ascaris lumbricoides* and Hookworm	3 (9.4%)	5 (16.7%)	1 (2.9%)	4 (12.9%)

Data are n (%) or mean (standard deviation (SD)). EPG = eggs per gram of stool. OXF = Oxfendazole. Percentages might not add up to 100% due to rounding.

Egg count at day 18 differed significantly between the placebo group and 3 × 800 mg oxfendazole (p < 0.001; probabilistic index = 0.81) but not between the placebo group and the two single-dose treatments (p = 0.17; probabilistic index = 0.57 for 1 × 400 mg and p = 0.13; probabilistic index = 0.61 for 1 × 800 mg) (Supplementary Table S4). The observed cure rate for *Trichuris* spp. was higher with 3 × 800 mg oxfendazole at 75.9% (95% CI 57.9–87.8) compared to the two single-dose treatments 1 × 400 mg and 1 × 800 mg oxfendazole at 31.0% (95% CI 17.3–49.2) and 35.3% (95% CI 21.5–52.1), respectively. The placebo group had a cure rate of 20.0% (95% CI 9.5–37.3) (Table 2). The difference between the placebo group and 3 × 800 mg oxfendazole was significant (p < 0.001) whereas the difference between the two single-dose treatments was not (both p = 0.17). Sensitivity analyses repeating the primary analyses using exact tests are presented in the supplement (Supplementary Table S5).

**Table 2:** Observed cure rates and egg reduction rates for *Trichuris* spp. in the mITT population. Data are % (95% confidence interval) unless specified otherwise.

	Placebo (N = 30)	1 × 400 mg OXF (N = 29)	1 × 800 mg OXF (N = 34)	3 × 800 mg OXF (N = 29)
Day 18 Egg Count (Mean EPG (SD))	1099.2 (3354.6)	218.3 (506.4)	148.4 (244.7)	36.2 (117.5)
Cured (n)	6	9	12	22
Observed cure rates	20.0% (9.5–37.3)	31.0% (17.3–49.2)	35.3% (21.5–52.1)	75.9% (57.9–87.8)
Observed cure rates by infection intensity at baseline				
Light (% [n/N])	24.0% (6/25)	39.1% (9/23)	36.7% (11/30)	85.0% (17/20)
Moderate and heavy (% [n/N])	0.0% (0/5)	0.0% (0/6)	25.0% (1/4)	55.6% (5/9)
Egg reduction rates				
Observed geometric egg reduction rate	66.4% (28.3–84.7)	91.3% (81.9–96.1)	90.2% (78.2–95.9)	99.7% (99.2–99.9)
Observed arithmetic egg reduction rate	−14.3% (−123.1 to 67.6)	83.1% (59.0–94.0)	64.1% (41.3–81.7)	97.8% (93.4–99.5)

Data are % (95% CI) unless specified otherwise. OXF = Oxfendazole. EPG = eggs per gram of stool. SD = standard deviation.

The observed geometric egg reduction rate was 99.7% (99.2–99.9) for 3 × 800 mg oxfendazole, 90.2% (78.2–95.9) for 1 × 800 mg oxfendazole, 91.3% (81.9–96.1) for 1 × 400 mg oxfendazole and 66.4% (28.3–84.7) for the placebo arm (Table 2, Fig. 2). Arithmetic mean egg counts and egg reduction rates are presented in Table 2.

**Fig. 2:**
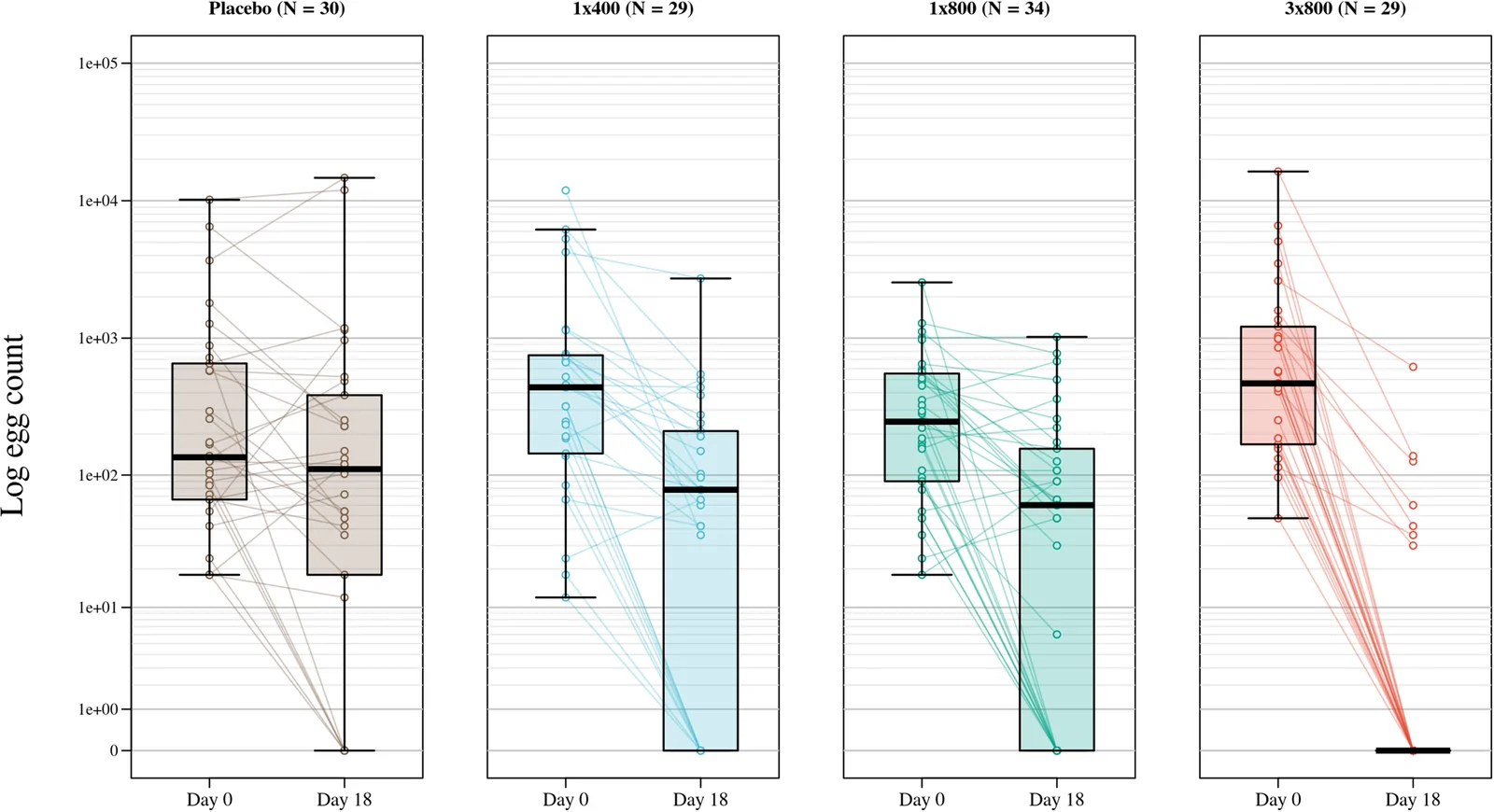
Log egg counts per gram of stool displayed for every participant at baseline (day 0) and follow-up (day 18), by treatment arm. Every point represents a single participant, paired values (same participant at day 0 and day 18) are connected with a line. The boxplots represent the median, 25% and 75% percentile (lower and upper limit, respectively).

In a post hoc analysis stratifying the treatment groups by baseline infection intensity for *Trichuris* spp., oxfendazole did not cure any participants with moderate to heavy infection in the 1 × 400 mg arm (0/6) and cured 39.1% of participants with light infection (9/23). In the 1 × 800 mg arm, 25.0% (1/4) participants with moderate to heavy infection—and 36.7% (11/30) of participants with light infection—were cured. On the other hand, with 3 × 800 mg oxfendazole, 85.0% (17/20) of participants with light infection were cured, as were 55.6% of those with moderate to heavy infection [5/9]. For comparison, none of the participants with moderate to high infection were cured in the placebo group (0/5) while a cure rate of 24.0% (6/25) was observed in participants harbouring a light infection and treated with placebo (Table 2).

Co-infections with *A. lumbricoides* or hookworm in each treatment arm were rather low and ranged from 18.8% to 40.0% (Table 1). Complete clearance of *A. lumbricoides* and hookworm infections was observed in all participants receiving oxfendazole, independent of the dose, corresponding to cure rates of 100.0% (Supplementary Tables S6 and S7, Supplementary Fig. S1a and b). No cures were observed among participants with *A. lumbricoides* infection who received placebo (0/6). However, a cure rate of 40.0% (4/10) was observed in participants with hookworm infection in the placebo group.

Arithmetic and geometric egg reduction rates of *Trichuris* spp., *A. lumbricoides* and hookworm grouped by baseline infection intensity are shown in Supplementary Table S8.

During the study, no serious adverse events were reported. In total, 37 adverse events were reported of which 10 (27.0%) were reported from participants receiving 3 × 800 mg oxfendazole, 12 (32.4%) receiving 1 × 800 mg oxfendazole, five (13.5%) receiving 1 × 400 mg oxfendazole and 10 (27.0%) receiving placebo (Table 3). Across all treatment groups, all observed adverse events were mild, with the exception of one event classified as moderate severity in arm 1 × 800 mg oxfendazole (panic attack, not related to IMP). The majority of adverse events were reported within the first day of treatment administration (14/37, 37.8%) on which most participants received the active treatment compared to the second and third treatment days. All adverse events were self-limiting within one to 17 days after first dose, apart from five adverse events with an unknown start or stop date as participants did not remember (Supplementary Table S9). The most common adverse event was abdominal discomfort/pain (12/37, 32.4%) four of which were in the 3 × 800 mg treatment group, two in the 1 × 800 mg treatment group, one in the 1 × 400 mg treatment group and five in the placebo group. Adverse events were classified as related to the IMP if there was a reasonable possibility of a causal relationship. Among the 37 adverse events, 28 (75.7%) were considered related. Seven occurred in participants receiving 3 × 800 mg, seven in participants receiving 1 × 800 mg dose, five in participants receiving a 1 × 400 mg dose, and nine in participants assigned to placebo (Supplementary Table S9).

**Table 3:** Reported adverse events after treatment (safety/ITT population).

	Placebo (N = 32)	1 × 400 mg OXF (N = 30)	1 × 800 mg OXF (N = 34)	3 × 800 mg OXF (N = 31)
Adverse events 1st treatment day				
Abdominal discomfort/pain (mild)	2 (6.2%)	0	2 (5.9%)	2 (6.5%)
Dizziness (mild)	2 (6.2%)	0	3 (8.8%)	0
Headache (mild)	0	0	1 (2.9%)	1 (3.2%)
Nausea (mild)	0	0	1 (2.9%)	0
Adverse events 2nd treatment day				
Abdominal discomfort/pain (mild)	2 (6.2%)	0	0	2 (6.5%)
Headache (mild)	1 (3.1%)	1 (3.3%)	0	0
Adverse events 3rd treatment day				
Abdominal discomfort/pain (mild)	0	1 (3.3%)	0	0
Chest pain (mild)	0	0	0	1 (3.2%)
Diarrhoea (mild)	1 (3.1%)	0	1 (2.9%)	0
Dizziness (mild)	0	0	0	1 (3.2%)
Headache (mild)	0	1 (3.3%)	0	0
Nausea (mild)	0	2 (6.7%)	0	1 (3.2%)
Palpitations (mild)	0	0	1 (2.9%)	0
Panic attack (moderate)	0	0	1 (2.9%)	0
Adverse events day 4				
Headache (mild)	1 (3.1%)	0	0	0
Adverse events with unknown start date after treatment				
Abdominal discomfort/pain (unknown)	1 (3.1%)	0	0	0
Influenza (mild)	0	0	1 (2.9%)	0
Pain in extremity (mild)	0	0	0	1 (3.2%)
Pollakiuria (mild)	0	0	0	1 (3.2%)
Pyrexia (unknown)	0	0	1 (2.9%)	0
Total adverse events	10 (27.0%)	5 (13.5%)	12 (32.4%)	10 (27.0%)

Data are n (%) unless specified otherwise. OXF = Oxfendazole. Symptoms start date as participants reported. Symptoms recorded on day 1, 2, 3, 4, 18 and 47 (only for WOCBP). Next to each adverse event, the severity is provided in parentheses, “unknown” indicates that no information could be collected regarding the severity of the adverse event.

Two participants tested positive for pregnancy at day 47 despite negative tests at day 18 (>5 terminal half-lives after oxfendazole administration). Both pregnancies resulted in the delivery of healthy infants. Unblinding showed that both participants had received a single 800 mg dose of oxfendazole.

No clinically relevant changes in vital signs were observed between treatment groups or across study visits (Supplementary Table S10). Physical and neurological examinations revealed no new findings at day 18 (Supplementary Tables S11 and S12). At day 18, three participants had elevated liver enzyme values compared with baseline (two in the 3 × 800 mg group and one in the 1 × 400 mg group). However, all values remained below three times the upper limit of laboratory normal range and were not considered clinically significant; all had returned to the normal range by day 47 when it was repeated (Supplementary Table S13). Haematological parameters remained largely unchanged, except for elevated eosinophil levels in one participant who received 1 × 400 mg oxfendazole and elevated neutrophil levels in one participant who received 3 × 800 mg oxfendazole compared to baseline (Supplementary Table S14). No clinically relevant changes were observed in urinalysis results compared with baseline (Supplementary Table S15).

## Discussion

The widely used benzimidazoles, albendazole and mebendazole, show unsatisfactory treatment efficacy against *Trichuris* spp. highlighting the medical need for alternative treatment options.^6^ A promising alternative is oxfendazole, also a benzimidazole, which demonstrates efficacy against a broad range of intestinal nematodes infecting animals and is currently under development for infections in humans.^9^ To our knowledge, this is the first completed study to evaluate the activity of oxfendazole against *Trichuris* spp. and co-occurring soil-transmitted helminth infections in humans.

We conducted a double-blind, phase 2a, randomised controlled trial on Pemba Island, Tanzania. Our study shows, that oxfendazole administered in multiple doses achieved a higher cure rate (75.9%) compared to single doses (31.0% and 35.3%). The 20.0% cure rate observed in the placebo arm may be attributed to the well described variability in egg shedding, low worm burden, natural fluctuations in female worm fertility and diagnostic limitations of the Kato-Katz method,^24^, ^25^, ^26^ in particular as the placebo arm had the lowest baseline infection intensity. This phenomenon has been observed previously, with placebo groups showing considerable cure rates (e.g., 19.5% in a previous study).^27^

Our study supports previous findings that the efficacy of benzimidazoles is increased when multiple doses are administered. In our study, treatment with 3 × 800 mg oxfendazole achieved a promising cure rate of 75.9%. When considering only infections of light intensity, the observed cure rate of the post-hoc analysis reached 85.0% for oxfendazole. For comparison, in multiple-dose treatments, Steinmann et al. showed cure rates against *T. trichiura* of 56.2% (95% CI 41.2–70.5) with 3 × 400 mg albendazole and 70.7% (95% CI 57.3–81.9) with 3 × 500 mg mebendazole. Corresponding geometric egg reduction rates found by Steinmann et al. were 94.0% (95% CI 89.4–96.8) (3 × 400 mg albendazole) and 97.3% (95% CI 94.9–98.8) (3 × 500 mg mebendazole) which was slightly lower than our results with 3 × 800 mg oxfendazole (99.7%).^28^ It would be worthwhile evaluating a 3 × 400 mg oxfendazole regimen in the future. If the 3 × 400 mg regimen would achieve efficacy comparable to that of 3 × 800 mg oxfendazole, the total number of tablets required could be reduced by half, potentially improving treatment feasibility and reducing costs. Such a regimen would also benefit from the development of a 400 mg tablet formulation instead of the current 100 mg tablets, which could further simplify administration and enhance programmatic implementation. Ongoing studies are currently evaluating improved tablet formulations that may facilitate future large-scale use. Although multiple-dose regimens are less suitable for mass drug administration, they could become valuable alternatives if drug donations cease, supporting a targeted test-and-treat approach in which multiple-dose treatments are both feasible and effective.

The oxfendazole single-dose cure rates for *Trichuris* spp. found in this study were similar to the cure rates found by Moser et al. in their previously published network meta-analysis for single-dose regimen of albendazole and mebendazole (cure rates of 30.7% (95% CI 21.0–42.5) and 42.1% (95% CI 25.9–60.2) for albendazole and mebendazole, respectively).^6^ However, the egg reduction rates described by Moser et al. were substantially lower for both albendazole and mebendazole (49.9% (95% CI 39.0–60.6) and 66.0% (95% CI 54.6–77.3), respectively) than the egg reduction rates we observed for oxfendazole. In our study, single dose oxfendazole achieved high geometric egg reduction rates of 91.3% and 90.2% for 1 × 400 mg and 1 × 800 mg, respectively. This characteristic warrants further investigation *in vivo* and *in vitro* to determine whether oxfendazole may affect worm fecundity rather than directly causing worm death. If oxfendazole reduces worm fecundity, this could lead to an overestimation of drug efficacy when based on egg reduction rates.^29^

In addition, consistent with findings from previous studies on other benzimidazoles, a single dose of oxfendazole showed excellent cure rates in participants infected with *A. lumbricoides* and hookworm. However, the prevalence of these co-infections was low in the study population and hookworm cure rates in the placebo arm was high, therefore further studies are needed to confirm the high efficacy observed in this study.

Oxfendazole was generally well tolerated, with most adverse events being mild in severity, and demonstrated an adverse event profile comparable to that reported for albendazole and mebendazole despite its longer half-life and advantageous pharmacokinetic profile.^28^, ^30^ Comparable safety findings were reported in the phase 1 clinical trials.^10^, ^11^, ^13^ The most reported adverse event was abdominal pain, which may also be attributable to the underlying disease itself.

This study comes with some limitations. First, we did not reach the anticipated sample size of 160 (144 with 10% drop-out) due to an unexpectedly high number of exclusions at the point of clinical examination. We treated 127 participants—and collected primary endpoint data of for 122 of them. Second, no stratification by infection intensity was applied to ensure similar baseline EPG levels across treatment arms, resulting in the multiple-dose arm having the highest baseline EPG values and potentially underestimated the efficacy of the multiple-dose arm. Third, clinical chemistry assessments were not performed at an earlier time point following IMP administration. Earlier testing (e.g., on day 3) may have identified additional transient laboratory abnormalities that were not captured by the study schedule.

Taken together, the comparison with published results for the widely used single-dose benzimidazoles albendazole or mebendazole in MDA suggests that a single-dose of oxfendazole is unlikely to offer an advantage over either drug, given the similarly low cure rates for the single-dose observed against *Trichuris* spp. Furthermore, as oxfendazole has the same mode of action as the other benzimidazoles, its introduction may not overcome potential resistance mechanisms. However, oxfendazole performed better than what has been reported for albendazole and mebendazole, in terms of egg reduction rates and revealed good activity when given at multiple doses. The development of oxfendazole is ongoing and even higher doses and longer treatment schedules (5 × 400 mg and 5 × 800 mg) are currently being tested for onchocerciasis, loiasis and mansonellosis within the eWHORM basket trial. While we await the outcomes of these studies, we speculate that a multiple-dose regimen of oxfendazole may provide an opportunity to address multiple co-endemic helminths simultaneously, potentially improving the efficiency of disease control and elimination programmes.

## Contributors

MH, SZ and JK designed the study. AS, JK and SMA planned the study. KDC provided trial management support. AS, HJ, ASM, ISM, RSA, SNS, AKA, MJK, SMA and JK implemented the study. AS, HJ, ASM, ISM, RSA, SNS, AKA, MJK and SMA collected the data. GMN and TWE were responsible for data management and randomisation. FB conducted medical checks in the database. SZ, OV, OS, FLM and AS analysed the data. AS, JK and SZ interpreted the data. OV, OS, AS and SZ created the data visualisations. AS wrote the first draft and manuscript revisions. JK, HJ, MH, KDC, SZ, OS, ASM and SMA reviewed and edited the manuscript. All authors had full access to all the data in the study, read and approved the final version of the manuscript, and had final responsibility for the decision to submit for publication. AS, OS and SZ directly accessed and verified the underlying data reported in the manuscript.

## Data sharing statement

De-identified individual participant data and the data dictionary, as well as supporting clinical trial documents, will be available upon request beginning at the time of publication for 1 year thereafter. Access will be granted to researchers who provide a methodologically sound proposal. Proposals will be assessed and approved by the sponsor, investigators, and collaborators based on scientific merit. Requests should be directed to the corresponding author. A data access agreement will be required before access is granted.

## Declaration of generative AI and AI-assisted technologies in the manuscript preparation process

During the preparation of this work the author(s) used ChatGPT for language editing, and grammatical correction. After using this tool, the author(s) reviewed and edited the content as needed and take(s) full responsibility for the content of the published article.

## Declaration of interests

We declare no competing interests.

## Appendix A Supplementary data

Supplementary Fig. S1 and Table S1–S15

Protocol_v2
