# Supplementary material for: Efficacy and safety of oxfendazole in adults with Trichuris spp. infection in Pemba Island, Tanzania: a double-blind, phase 2a, randomised controlled trial
Source: eClinicalMedicine. 2026 Oct 26;101:104273. doi: 10.1016/j.eclinm.2026.104273 (PMC13734800; doi:10.1016/j.eclinm.2026.104273)
Supplement: Supplementary file 1 [file mmc2.pdf]

Clinical Trial Protocol

**Protocol Title:**	A phase IIa, multi-country, randomized, placebo-controlled, double-blinded, adaptive, basket trial to assess the efficacy and safety of oxfendazole in adults with trichuriasis, and /or mansonellosis, and/or onchocerciasis and/or loiasis
**Brief Title:**	A clinical trial to investigate efficacy and safety of oxfendazole compared with placebo in adults with trichuriasis, and /or mansonellosis, and/or onchocerciasis and/or loiasis
**Protocol Number:**	OXF-01
**Protocol Version /Date:**	Version 2.0 / 15 November 2024
**Amendment number / Date**	N/A
**Compound(s) and Drug Class(s):**	Oxfendazole<br>Anthelmintic
**Clinical trial Phase:**	Phase IIa
**Sponsor Name and Legal Registered Address:**	Swiss Tropical and Public Health Institute<br>Kreuzstrasse2, 4123 Allschwil<br>Switzerland
**Regulatory Agency Identifier Number(s):**	Oxfendazole CAS number: 53716-50-0

The information contained in this document is confidential. It is to be used by investigators, potential investigators, consultants, or applicable independent ethics committees. It is understood that this information will not be disclosed to others without written authorization from sponsor, except where required by applicable local laws.

Sponsor's Signature

Sponsor Representative Signatory:

DocuSigned by:
Jennifer Keiser
E8E6BD544F39400...

18-Dec-2024 | 11:24:05 CET

Prof. Dr. Jennifer Keiser
Swiss Tropical and Public Health Institute
Kreuzstrasse2, 4123 Allschwil
Switzerland

Date

Sponsors Medical Responsible Name and Contact Information:

Prof. Dr. Daniel Paris
Swiss Tropical and Public Health Institute
Kreuzstrasse2, 4123 Allschwil
Switzerland

Protocol Amendment Summary of Changes Table

Not applicable for the current version of the protocol.

Table of Contents

Clinical Trial Protocol 1
Sponsor's Signature 2
Protocol Amendment Summary of Changes Table 3

Table of Contents 4

List of Tables/Figures 8

List of Abbreviations 9

Protocol Summary 11

Schedule of Activities (SoA) 17
Schedule of Activities 1: Arms 0-9 17
Schedule of Activities 2: Arm 10, 11 only 19

1. Introduction 21
1.1. Background 22
1.1.1. Summary of non-clinical data 22
1.1.2. Summary of clinical data 23
1.2. Clinical Trial Rationale 25
1.3. Benefit/Risk Assessment 27

2. Objectives, Endpoints, and Estimands 29
2.1. Summary 29
2.2. Primary Estimands 30
2.3. Secondary Estimand(s) 31
2.4. Exploratory Estimand(s) 32

3. Clinical Trial Design 33
3.1. Overall Design 33
3.2. Scientific Rationale for Clinical Trial Design 34
3.3. Justification for Dose 37
3.4. End-of-Clinical Trial Definition 40

4. Clinical Trial Population 42
4.1. Inclusion Criteria 42
4.2. Exclusion Criteria 43
4.3. Lifestyle Considerations 44
4.3.1. Meals and Dietary Restrictions 44
4.3.2. Alcohol 44
4.4. Screen Failures 45
4.4.1. Rescreening Criteria 45
4.5. Criteria for Temporarily Delaying 45

5. IP(s) and Concomitant Therapy 46
5.1. IP(s) Administered 46
5.2. Preparation, Handling, Storage, and Accountability 48
5.3. Assignment to IP 49
5.4. Blinding, Masking 49
5.5. IP Administration Compliance 50

5.6. Dose Modification 50
5.7. Continued Access to IP after the End of the Clinical Trial 50
5.8. Treatment of Overdose 50
5.9. Prior and Concomitant Therapy 51
5.9.1. Rescue Medication and End of Clinical Trial medication 51
5.9.2. Permitted Concomitant Therapy 51
5.9.3. Prohibited Concomitant Therapy 51

6. Discontinuation of IP Administration and Participant Discontinuation/Withdrawal 53
6.1. Discontinuation of IP 53
6.1.1. Liver Chemistry Stopping Criteria 53
6.1.2. Hematologic Stopping Criteria 53
6.1.3. Temporary Discontinuation 53
6.2. Participant Discontinuation/Withdrawal from the Clinical Trial 54
6.3. Lost to Follow up 54

7. Clinical Trial Assessments and Procedures 55
7.1. Assessments and Procedures 55
7.1.1. Screening 55
7.1.2. Baseline 55
7.1.3. Randomisation and Treatment Period 56
7.1.4. Follow-Up Period 57
7.1.5. Unscheduled visits 58
7.2. Baseline Procedures 59
7.2.1. Demographic Data 59
7.2.2. Medical History 59
7.2.3. Concomitant Medication 59
7.2.4. Incidental Findings 59
7.3. Efficacy Assessments 60
7.3.1. *Onchocerca volvulus* 60
7.3.2. *Loa loa* 60
7.3.3. *Mansonella perstans* 60
7.3.4. *Trichuris trichiura* 60
7.4. Safety Assessments 61
7.4.1. Physical Examinations 61
7.4.2. Vital Signs 61
7.4.3. Neurological Assessment 61
7.4.4. Clinical Safety Laboratory Tests 61
7.4.5. Pregnancy Testing 62
7.4.6. Urine Test 62
7.5. Adverse Events, Serious Adverse Events, and Other Safety Data Collection, Assessment and Reporting 63
7.5.1. Time Period and Frequency for Collecting AE and SAE Information 63
7.5.2. Method of Detecting AEs and SAEs 64
7.5.3. Reporting of SAE 64
7.5.4. Follow-up of AEs and SAEs 64

7.5.5. Regulatory Reporting Requirements for SAEs 65
7.5.6. Exposure During Pregnancy 65
7.6. Pharmacokinetics 66
7.7. Pharmacodynamics 67
7.8. Genetics 67
7.9. Biomarkers 67
7.10. Immunogenicity Assessments 67
7.11. Medical Resource Utilization and Health Economics 67

8. Statistical Considerations 68
8.1. Statistical Hypotheses 68
8.1.1. Multiplicity Adjustment 68
8.2. Analysis Sets 68
8.3. Statistical Analyses 69
8.3.1. General Considerations 69
8.3.2. Primary Endpoint/Estimand Analysis 69
8.3.3. Secondary Endpoint(s)/Estimand(s) Analysis 72
8.3.4. Safety Analyses 72
8.3.5. Other Analyses 72
8.4. Sample Size Determination 73

9. Supporting Documentation and Operational Considerations 77
9.1. Appendix 1: Regulatory, Ethical, and Trial Oversight Considerations 77
9.1.1. Regulatory and Ethical Considerations 77
9.1.2. Informed Consent Process 77
9.1.3. Recruitment strategy 78
9.1.4. Data Protection 78
9.1.5. Committees Structure 79
9.1.6. Dissemination of Clinical Trial Data 79
9.1.7. Data Quality Assurance 80
9.1.8. Source Documents 80
9.1.9. Trial and Site Start and Closure 81
9.1.10. Publication Policy 82
9.1.11. Insurance 82
9.1.12. Funding 82
9.2. Appendix 2: Clinical Laboratory Tests 83
9.3. Appendix 3: AEs and SAEs: Definitions and Associated Instructions 84
9.3.1. Definition of AE 84
9.3.2. Definition of SAE 85
9.3.3. Recording and Follow-Up of AE and/or SAE 86
9.4. Appendix 4: Contraceptive and Barrier Guidance 90
9.5. Appendix 5: Country-specific Requirements 91
9.6. Appendix 6: Suggested Actions and Follow-up Assessments [and IP Restart/Rechallenge Guidelines] 96
9.6.1. Liver Safety Management 96
9.6.2. Hematologic Safety Management 97

9.7. Appendix 7: Protocol Amendment History 98
9.8. Appendix 8: Adverse Event Severity Grading 99
10. References 100

List of Tables/Figures

Table 1	Description of the Diseases	26
Table 2	Dosing illustration for the fixed doses used in OXF-01	39
Table 3	Percentage of participants achieving target concentrations (tissue nematodes) for a minimum of 1-5 days	40
Table 4	Meals and Dietary Restrictions	44
Table 5	Alcohol	44
Table 6	IP(s) Administered	46
Table 7	Clinical Trial Arm(s)	47
Table 8	Time-points and Time Windows for PK Sample Collection in stage 1	66
Table 9	Definition of scenarios of reduction rates and total responder rates (low dose (LD)/medium dose (MD)/high dose (HD)) for the power calculations for onchocerciasis, mansonellosis and loiasis	74
Table 10.	Power in % for low dose (LD)/medium dose (MD)/high dose (HD), defined as the probability that the dose is selected and that the corresponding null hypothesis is rejected.	75
Table 11.	Selection probabilities in % for low dose/medium dose/high dose, which is the frequency (%) the dose is selected for/started in stage 2 for scenarios efficacy only in the high dose, linear trend in effect sizes, and equal efficacy across all doses.	75
Table 12.	Definition of scenarios of reduction rates and total responder rates at day 18 and resulting power in % for low dose (LD)/medium dose (MD)/high dose (HD) for trichuriasis	76
Table 13	Considered baseline parameters for mean and standard deviation of the raw values for each disease	76
Table 14	Protocol-required Safety Laboratory Tests	83
Figure 1	Study Design Overview	35
Figure 2	Dosing scenarios with 100, 400, and 800 mg oxfendazole	41

List of Abbreviations

Abbreviation	Definition
AE	Adverse Event
ALT	Alanine Aminotransferase
AST	Aspartate aminotransferase
AUC	Area under the curve
BCS	Biopharmaceutics Classification System
C	Concentration
CR	Cure rates
CRF	Case Report Form
CSF	Child Surveillance Form
D	Day
DALYS	Disability Adjusted Life Years
DEC	diethylcarbamazine citrate
DSMB	Data Safety Monitoring Board
ECG	Electrocardiogram
eCRF	Electronic Case Report Form
EPG	Eggs per gram
GCP	Good Clinical Practice
GLP	Good Laboratory Practice
HED	Human Equivalent Dose
HIV	Human Immunodeficiency Virus
IB	Investigator's Brochure
ICE	Intercurrent Events
ICF	Informed Consent Form
ICH	International Council for Harmonization of Technical Requirements for Pharmaceuticals for Human Use
IEC	Independent Ethics Committee
IP	Investigational Product
IRB	Institutional Review Boards
ITT	Intention to treat
IUD	Intrauterine device
IUS	Intrauterine hormone-releasing system
M	Month
MAD	Multiple Ascending Dose

Abbreviation	Definition
MDA	Mass Drug Administration
mf	Microfilariae
mITT	Modified Intent-to-treat
NIAID	National Institute of Allergy and Infectious Diseases
NOAEL	No Observed Adverse Effect Level
NTD	Neglected Tropical Disease
ODG	Oxfendazole Development Group
OXF	Oxfendazole
PCR	Polymerase Chain Reaction
PP	Per protocol
PK	Pharmacokinetics
PSF	Pregnancy Surveillance Form
QTc	Corrected QT Interval
R_{acc}	Accumulation ratio
SAD	Single Ascending Dose
SAE	Serious Adverse Event
SAR	Serious Adverse Reaction
SAP	Statistical Analysis Plan
SoA	Schedule of Activities
SOP	Standard Operating Procedure
STH	Soil-transmitted Helminth
SUSAR	Suspected Unexpected Serious Adverse Reaction
$t_{½}$	Half-life
T	Duration
ULN	Upper Limit of Normal
WBC	White Blood Cells
WHO	World Health Organization
WOCBP	Women of Childbearing Potential

Protocol Summary

Synopsis summary table

**Protocol Title**	A phase IIa, multi-country, randomized, placebo-controlled, double-blinded, adaptive, basket trial to assess the efficacy and safety of oxfendazole in adults with trichuriasis, and /or mansonellosis, and/or onchocerciasis and/or loiasis
**Brief Title**	A clinical trial to investigate efficacy and safety of oxfendazole compared with placebo in adults with trichuriasis, and /or mansonellosis, and/or onchocerciasis and/or loiasis
**Rationale and Brief Summary**	Oxfendazole (Biopharmaceutics Classification System (BCS) class II drug, i.e. drug with low solubility and high permeability) offers a number of advantages that are desirable for a new anthelmintic agent:<br>• As with other well-known anthelmintic drugs belonging to the benzimidazole class, oxfendazole has shown potent activity against a broad range of nematodes;<br>• The pharmacokinetics, safety and broad spectrum of efficacy of oxfendazole are consistently demonstrated in intestinal helminth infections in animals, as well as tissue-dwelling larval cestode and trematode infections in diverse animal species. Oxfendazole is a registered drug for veterinary use;<br>• Published first-in-human safety and pharmacokinetic data have shown its safety in humans; at acceptable exposure<br>• Oxfendazole acts against adult worms, but not the microfilarial stage and is thus a promising drug candidate that could be used in *Loa*-co-endemic areas.<br><br>Thus, oxfendazole represents a promising candidate to expand the limited portfolio of antiparasitic drugs available to treat helminth infections in humans.
**Objectives and Endpoints**	Primary objective:<br>To identify a safe and efficient dose of oxfendazole for each of the four indications: participants infected with one or more of the following: *Onchocerca volvulus* (onchocerciasis), *Loa Loa* (loiasis), *Mansonella perstans* (mansonellosis) or *Trichuris trichiura* (trichuriasis).<br><br>Primary endpoint:<br>Parasite load at day 18 (egg count for trichuriasis) and/or month 12 (microfilaria (mf) count for all other indications).<br><br>Secondary objectives:

	To demonstrate the safety, tolerability, efficacy and pharmacokinetics characteristics of oxfendazole in helminth infected individuals.<br><br>Secondary endpoints:<br>• Frequency of adverse events (AEs) and serious adverse events (SAEs) up to day 18 (and day 45 for Arms 10, 11)<br>• Frequency of adverse events (AEs) and serious adverse events (SAEs) up to the end of the trial<br>• PK parameters (AUC0-t, AUC0-∞, AUCtau, Cmax, Tmax, t1/2 and Racc) of oxfendazole<br><br>Disease specific secondary endpoints:<br><br>*Onchocerca volvulus* infection<br>• Relative reduction of skin mf at day 18 (and day 45 for Arms 10, 11), month 3, 6, 9 and 12<br>• Absence of live female adult worms with normal embryogenesis (assessed by histological examination of nodules collected at month 12)<br>• Absence of skin mf at day 18 (and day 45 for Arms 10, 11), month 3, 6, 9 and 12<br>• Absence of signs and symptoms of onchocerciasis day 18 (and day 45 for Arms 10, 11), month 3, 6, 9 and 12<br><br>*Loa loa* infection<br>• Relative reduction of *L. loa* blood mf at day 18 (and day 45 for Arms 10, 11), month, 3, 6, 9 and 12<br>• Area under the curve of *L. loa* blood mf during follow-up period for each dose level<br>• Absence of blood mf at day 18 (and day 45 for Arms 10, 11), month 3, 6, 9 and 12<br>• Absence of signs and symptoms due to eye worm migration, Calabar swelling, and dermal migration at day 18 (and day 45 for Arms 10, 11), month 3, 6, 9 and 12<br><br>*Mansonella perstans* infection<br>• Relative reduction of *M. perstans* mf at day 18 (and day 45 for Arms 10, 11), month, 3, 6, 9 and 12<br>• Area under the curve of *M. perstans* blood mf during follow-up period for each dose level<br>• Absence of *M. perstans* blood mf at day 18 (and day 45 for Arms 10, 11), month 3, 6, 9 and 12<br>• Absence of signs and symptoms of pruritus, urticaria and oedema at day 18 (and day 45 for Arms 10, 11), month 3, 6, 9 and 12<br><br>*Trichuris trichiura* infection

	• Relative reduction of eggs (egg reduction rate) at day 18 (assessed by quadruplicate Kato-Katz)<br>• Absence of eggs (cure rate) at day 18 (assessed by quadruplicate Kato-Katz)<br>• Relative reduction of eggs (egg reduction rate) at day 18 (assessed by quadruplicate Kato-Katz) for additional soil-transmitted helminth (STH) infections (*Ascaris lumbricoides* and hookworm)<br>• Absence of eggs (cure rate) at day 18 (assessed by quadruplicate Kato-Katz) for additional STH infections (*Ascaris lumbricoides* and hookworm)<br><br>Exploratory objectives:<br>• Facultative subgroup analysis to demonstrate efficacy characteristics of oxfendazole in co-infection with other helminth infections.<br>• Assessment of parasite DNA in the samples at day 18 (trichuriasis) and month 12 (all other infections) (PCR and loop-mediated isothermal amplification (LAMP)) for helminth infections, from skin snip, blood, and stool samples.
**Overall Clinical trial Design**	Multi-country, randomized, placebo-controlled, double-blinded. Study day 18 (47 days post last dose for WOCBP) for *T. trichiura,* or month 12 (all other indications) is the final required study visit.
**IP**	Oxfendazole tablet 100 mg<br>Matching placebo for oxfendazole tablet 100 mg
**Clinical trial /Treatment Duration**	Clinical trial details include:<br>• The clinical trial duration for each participant will be up to day 18 (47 days post last dose for WOCBP) for *T. trichiura*, and month 12 for all other infections<br>• The treatment duration will be up to 5 days given once or twice one month apart<br>• The visit frequency will be daily during treatment, day 18 and 47 days post last dose for WOCBP (final follow-up visit for *T. trichiura*), day 45 (Arms 10, 11), and every 3 months up to month 12 for all other infections.
**Inclusion/ Exclusion Criteria**	Inclusion Criteria<br>1. Participant must be 18 or the legal age of consent in the jurisdiction in which the clinical trial is taking place to 65 years of age inclusive, at the time of signing the informed consent.

	2. A diagnosis of one or more of the following: trichuriasis, mansonellosis, onchocerciasis and loiasis confirmed within 3 months before randomization.<br>3. Body weight at screening ≥ 45 kg.<br>4. Willingness of women of child-bearing potential (WOCBP[1]) to use a culturally acceptable, highly effective method of contraception (see appendix 4), which is effective before the first IP intake and for at least 33 days after the last IP intake.<br>5. Willingness of male participants, in each course of study treatment: from first day of IP intake to 33 days after the last IP intake, to:<br>• refrain from donating sperm.<br>• use a male condom for vaginal intercourse with female partners of childbearing potential . Male participants should also be advised of the benefit for a female partner to use a highly effective method of contraception throughout the same duration, as a condom may break or leak.<br>6. Capable of giving signed informed consent which includes compliance with the requirements and restrictions listed in the informed consent form (ICF) and in this protocol.<br>7. Participant is deemed clinically suitable for a placebo-controlled study of the duration planned.<br><br>Disease Specific Inclusion Criteria:<br>*Onchocerca volvulus*<br>• Presence of at least 1 excisable subcutaneous nodule/ onchocercoma detected through palpation<br>• *O. volvulus* infection diagnosed by skin snip method: documented mf positivity on skin assessment on at least 2 out of 4 skin snips.

[1] The definition of WOCBP will be applied in the study as follows: a woman is considered of childbearing potential, i.e. fertile, following menarche and until becoming post-menopausal unless permanently sterile. Permanent sterilization methods include hysterectomy, bilateral salpingectomy and bilateral oophorectomy. A postmenopausal state is defined as no menses for 12 months without an alternative medical cause (Clinical Trials Facilitation and Coordination Group, 2020)

	Loa loa<br>• *Loa loa* infection as evidenced by peripheral microfilaremia assessed between 10am-4pm between 100-8,000 mf/ml<br>*Mansonella perstans*<br>• *M. perstans* infection diagnosed by mf-positivity in blood ≥ 200 mf/ml blood (10 mf via finger prick/~50µl)<br>*Trichuris trichiura*<br>• Being able and willing to provide two stool samples at screening and at follow-up assessment.<br>• Having at least two out of four Kato-Katz slides positive for *T. trichiura* at screening.<br><br>Exclusion Criteria:<br>1. Any condition, in the investigator's opinion, that places the participant at undue risk (symptoms, physical signs, vital signs or biological/laboratory signs suggestive of past or present systemic disorders, including but not limited to, cardiovascular, pulmonary, cutaneous, immunodeficiency, hepatic, renal, hematologic, psychiatric disorders, drug / alcohol abuse and other abnormalities likely to interfere with the interpretation of results of the trial) or to significantly affect the study outcomes as judged by the investigator.<br>2. Current or chronic history of liver disease. This includes (but is not limited to) hepatitis virus infections, drug- or alcohol-related liver disease, nonalcoholic steatohepatitis, autoimmune hepatitis, hemochromatosis, Wilson's disease, α-1 antitrypsin deficiency, primary biliary cholangitis, primary sclerosing cholangitis, or any other liver disease considered clinically significant by the investigator.<br>3. Pregnant and breastfeeding women<br>4. Haemoglobin below 10mg/dl at baseline<br>5. Aspartate aminotransferase/serum glutamic oxaloacetic transaminase (AST/SGOT) and/or alanine aminotransferase/serum glutamic pyruvic transaminase (ALT/SGPT) and/or total bilirubin > 1.5xULN at baseline.<br>6. Estimated Glomerular Filtration Rate (using the Modification of Diet in Renal Disease equation, (Beck, 2023)) < 60 mL/min at baseline.<br>7. More than one epileptic seizure within the past 5 years<br>8. *Paragonimus sp*. eggs or *Taenia* sp. proglottids or eggs detectable in stool

	9. Acute and/or febrile illness requiring therapy within 14 days prior to baseline<br>10. Anthelmintic therapy:<br>For STH: within previous 1 month prior baseline: ivermectin, moxidectin, diethylcarbamazine, albendazole, mebendazole<br>For all other diseases:<br>• within previous 6 months prior baseline: ivermectin, moxidectin, diethylcarbamazine, albendazole, mebendazole<br>• within previous 12 months: doxycycline (> 2 weeks course) prior baseline<br>11. Use of concomitant medication that is contraindicated with benzimidazoles and/or known hypersensitivity to benzimidazoles or any other ingredient in the oxfendazole tablet formulation<br>12. Current or planned use of another investigational drug at any point during study participation. Participation in any interventional study within 3 months prior to screening or during the study, or within 5 times the half-life of the drug in the previous clinical trial, whichever is longer (time calculated relative to final intake in previous trial)
**Sample Size**	Onchocerciasis, mansonellosis, loiasis 200 each (120 each for the 1st stage and 80 each for the second stage), trichuriasis 160
**Number of Participants**	Approximately 760 participants to be randomized
**Study Arms and Duration**	• Three days, once daily, oxfendazole 800 mg only for *T. trichiura*<br>• One day, single dose, oxfendazole 400 mg only for *T. trichiura*<br>• One day, single dose, oxfendazole 800 mg only for *T. trichiura*<br>• Five days, once daily, oxfendazole 400 mg, except for *T. trichiura*<br>• Five days, once daily, oxfendazole 800 mg, except for *T. trichiura*<br>• Five days, once daily, oxfendazole 800mg, repeated one month apart, except for *T. trichiura*<br>• Oxfendazole matching placebo
**Statistical Analyses**	Refer to Section 8.3
**Data Monitoring/Other Committee:**	A global Data Safety Monitoring Board (DSMB) will be appointed to monitor accumulating efficacy and safety data at intervals. The mission of the DSMB will be to safeguard the interests of study participants and to enhance the integrity and credibility of the trial. The DSMB will be asked to recommend continuation, adaptation or stopping the study. Information of membership, terms of reference, roles, responsibilities, authority, decision-making and relationships of the independent DSMB, including statistical decision principles, will be detailed in a separate DSMB charter.

Schedule of Activities (SoA)

Schedule of Activities 1: Arms 0-9

Arms 0-9<br>Study Period	Screening	Base-line	Random-isation	Treatment period					Follow up Period					
**Timepoint**	M-3 to D-1	D-2 to D-1	D-1 to D1	D1	D2	D3	D4[a]	D5[a]	D18[c]	47d post last dose	M3[a]	M6[a]	M9[a]	M12[a]
**Visit window**									± 3d	± 3d	± 7d	± 14d	± 21d	± 28d
Informed consent	X													
Medical history	X	X												
Concomitant Treatment	X	X	X	X	X	X	X	X	X		X	X	X	X
Demographic data		X												
Vital signs		X		X	X	X	X	X	X			X		X
Physical Examination - Complete		X							X			X		X
Physical Examination - Symptom directed				X							X		X	
Neurological Examination		X							X			X		X
WOCBP Pregnancy test	X	X							X	X				
Blood (safety)		X				X [d]		X	X					
Blood (PK)[a]								X						
Urine (safety)		X				X [d]		X	X					
Inclusion / Exclusion Criteria	X	X	X											

Randomisation			X											
Treatment				X	X	X	X	X						
Collection of Adverse Events	⟶													
Parasitological Assessment: *Onchocerca volvulus*														
Nodule Palpation	X								X		X	X	X	X
Skin Snip	X	X(b)							X		X	X	X	X
Signs and symptoms (disease related)		X							X		X	X	X	X
Nodulectomy														X
Parasitological Assessment: *Loa loa*														
Blood	X	X(b)							X		X	X	X	X
Signs and symptoms (disease related)		X							X		X	X	X	X
Parasitological Assessment: *Mansonella perstans*														
Blood	X	X(b)							X		X	X	X	X
Signs and symptoms (disease related)		X							X		X	X	X	X
Parasitological Assessment: *Trichuris trichiura*														
Stool	X								X					

a) Only for *O. volvulus* infection, *L. loa* infection and *M. perstans* infection
b) To be done if baseline visit is conducted more than 2 months apart from screening visit
c) Or early discontinuation
d) Only for *T. trichiura*

Schedule of Activities 2: Arm 10, 11 only

Arms 10, 11 Study Period	Screening	Base -line	Treatment period						Treatment period – repeat 1 month apart						Follow-Up Period					
**Timepoint**	M-3 to D-1	D-2 to D-1	D-1 to D1	D1	D2	D3	D4	D5	D26 to D27	D28	D29	D30	D31	D32	D45[(b)]	47d post last dose	M3	M6	M9	M12
**Visit window**									± 3d							± 3d	±7d	±14d	±21d	±28d
Informed consent	X																			
Medical history	X	X																		
Concomitant Treatment	X	X	X	X	X	X	X	X	X	X	X	X	X	X	X		X	X	X	X
Demographic data		X																		
Vital signs		X		X	X	X	X	X	X	X	X	X	X	X	X			X		X
Physical Examination – Complete		X							X						X			X		X
Physical Examination - Symptom directed				X						X							X		X	
Neurological Examination		X							X						X			X		X
WOCBP Pregnancy test	X	X							X						X	X				
Blood (safety)		X						X	X					X	X					
Urine (safety)		X						X	X					X	X					
Inclusion / Exclusion Criteria	X	X	X																	
Randomisation			X																	

**Arms 10, 11 Study Period**	**Screening**	**Base-line**	**Treatment period**						**Treatment period – repeat 1 month apart**						**Follow-Up Period**					
**Timepoint**	M-3 to D-1	D-2 to D-1	D-1 to D1	D1	D2	D3	D4	D5	D26 to D27	D28	D29	D30	D31	D32	D45[(b)]	47d post last dose	M3	M6	M9	M12
**Visit window**									± 3d							± 3d	±7d	±14d	±21d	±28d
Treatment				X	X	X	X	X		X	X	X	X	X						
Collection of Adverse Events	→	→	→	→	→	→	→	→	→	→	→	→	→	→	→	→	→	→	→	→
Parasitological Assessment: *Onchocerca volvulus*																				
Nodule Palpation	X														X		X	X	X	X
Skin Snip	X	X[(a)]													X		X	X	X	X
Signs and symptoms (disease related)		X													X		X	X	X	X
Nodulectomy																				X
Parasitological Assessment: *Loa loa*																				
Blood	X	X[(a)]													X		X	X	X	X
Signs and symptoms (disease related)		X													X		X	X	X	X
Parasitological Assessment: *Mansonella perstans*																				
Blood	X	X[(a)]													X		X	X	X	X
Signs and symptoms (disease related)		X													X		X	X	X	X

a) To be done if baseline visit is conducted more than 2 months apart from screening visit
b) Or early discontinuation

1. Introduction

Only a very limited number of drugs are available for the treatment of neglected tropical diseases (NTDs) and in particular helminth infections, such as soil-transmitted helminths or filariae. Global efforts to eliminate nematodes in humans or to provide individual care for patients are hampered by the absence of effective vaccines and of medicines that kill adult worms (macrofilaricidal) using a short treatment regimen. Oxfendazole (OXF) has an excellent adult worm killing efficacy in livestock and companion animals as well as rodent filarial models. Recent work on oxfendazole has enhanced our knowledge of its nonclinical safety profile and demonstrated its safety and bioavailability in healthy human volunteers, thus advancing the possibility of a new and much needed option for antiparasitic treatment of soil-transmitted helminths and tissue parasites.

Oxfendazole (CAS: 53716-50-0, methyl [5-(phenylsulphinyl)-1*H*-benzimidazole-2-yl] carbamate) is a benzimidazole anthelmintic which is used in oral and topical formulations indicated for the treatment of gastrointestinal roundworms, lung worms and tapeworms in cattle, sheep, horses and dogs. It has been approved for veterinary use in the United States (US), the European Union (EU) since the early 1990s and it is also registered in Tanzania (TZ 18 V 0002). It is used in sub-Saharan Africa including mainland Tanzania to control cestode infection in pigs, thus indirectly reducing the transmission of *Taenia solium* to humans. However, a direct treatment of helminth infection in humans is not possible due to the lack of a suitable formulation and its registration for use in humans. Oxfendazole has a particularly long metabolic half-life in ruminants, and its active metabolite fenbendazole also has anthelmintic action (European Medicines Agency, 2004). Like other benzimidazole drugs, it causes degeneration of the parasite microtubule and irreversibly blocks glucose uptake in parasites. Inhibition of glucose uptake causes depletion of energy stores in parasites, eventually resulting in death. However, there is no effect on glucose metabolism in mammals (Gonzalez et al., 2019).

Importantly, OXF has no direct activity against microfilariae (mf), which is expected to avoid mf-induced inflammatory responses in loiasis and onchocerciasis patients, preventing drug-induced serious adverse events (SAE), as are observed following diethylcarbamazine citrate (DEC) or ivermectin treatment in onchocerciasis or loiasis patients (Hübner et al., 2020; Pionnier et al., 2019). OXF, targeting tubulin, has the most promising characteristics, including low cost of goods, target structure present in all nematodes and proven nematocidal activity in veterinary parasitosis (European Medicines Agency, 1997). If proven effective, it will be a major leap towards expansion of the anthelmintic toolkit and ultimately for global health equity. We will address the efficacy and safety profile of OXF as the first paradigmatic drug candidate for *O. volvulus*, *L. loa*, *M. perstans* and/or *T. trichuria* infected patients, aiming to broaden the therapeutic area. Repurposing of an existing drug will allow reduction of development costs and timelines and has a strong potential for an enormous public health benefit by targeting multiple diseases.

Oxfendazole has been tested in two phase I clinical trials. No serious adverse reactions (SAR) occurred in the single ascending dose (SAD) (An et al., 2019) and a multiple ascending dose (MAD) (Bach et al., 2020) study conducted by the Oxfendazole Development Group (ODG) under the auspices of National Institute of Allergy and Infectious Diseases (NIAID) in healthy volunteers with a liquid formulation. Oxfendazole administered orally in a liquid formulation was found to be safe and well tolerated at doses up to 60 mg/kg and showed acceptable safety and tolerability

profiles after five repeated daily doses of up to 15 mg/kg, with a few reports of non-serious adverse events (AEs) in the two studies. There was no increase in AEs with increasing dose. No serious adverse events or deaths were reported. In addition, a Phase I bioavailability study has been performed with the immediate release tablet formulation (Clinicaltrials.gov NCT04920292). Oxfendazole was overall well tolerated and no AEs were reported in this study. Moreover, there were no clinically significant abnormal results in laboratory, vital signs, physical examinations and ECG safety parameters over time. Exposure was comparable to that of the SAD and MAD studies.

Oxfendazole therefore offers a number of advantages that are desirable for a novel pan-nematicidal agent:

- As with other well-known anthelmintic drugs belonging to the benzimidazole class, oxfendazole has shown potent activity against a broad range of nematodes;
- The pharmacokinetics, safety and broad spectrum of efficacy of oxfendazole are consistently demonstrated in intestinal helminth infections in animals, as well as tissue-dwelling larval cestode and trematode infections in diverse animal species. Oxfendazole is a registered drug for veterinary use;
- Published first-in-human safety and pharmacokinetic data have shown its safety in humans;
- At acceptable exposure oxfendazole acts against adult worms, but not the microfilarial stage and is thus a promising drug candidate that could be used in *Loa*-co-endemic areas and due to its broad range activity even as safe drug against loiasis.

Thus, oxfendazole represents a promising candidate to expand the limited portfolio of antiparasitic drugs available to treat helminth infections in humans, including *Onchocerca volvulus, Loa loa, Mansonella perstans, Wuchereria bancrofti* and *Trichuris trichuria.*

1.1. Background

1.1.1. Summary of non-clinical data

An extensive data set on the non-clinical toxicity of oxfendazole is publicly available and it was therefore not necessary to conduct any nonclinical studies at this point.

A variety of toxicity studies (with Good Laboratory Practice (GLP) status not specified in the respective publications) with different exposure (from single dose and up to 1 year duration) have been published. Liver, bone marrow and reproductive organs appear to be the target organs of toxicity. Toxicity observed in various animal species was found after long term treatments. In rats, hepatic vacuolation at 11 mg/kg/day after 14 days, hepatic hypertrophy and necrosis at 49.9 mg/kg/day after 3 months, testicular atrophy at 5 mg/kg/day after 14 days, splenic necrosis or atrophy and bone marrow hyperplasia at 49.9 mg/kg/day after 3 months were observed. In mice, increased liver weights and fatty vacuolation at 75 mg/kg/day for 3 months as well as elevated clinical chemistry (SGPT (=ALT), serum alkaline phosphatase and SGOT (=AST)) at 3000 mg/kg/day after one month and reduced testicular weights and hypospermatogenesis at 300 mg/kg after 3 months were reported. In dogs, reduced myeloid maturation, reduction of splenic lymphoid tissue and thymic atrophy were observed following a 14-day oral treatment with 11 mg/kg/day oxfendazole (Hallesy et al., 1976). Moreover, downward trends for erythrocyte count, hemoglobin,

and hematocrit were observed in Beagle dogs in a 1-year study with dosages of up to 13.5 mg/kg. However, values remained within the physiological range (Fuchs, n.d.). As reference the human efficacious dose is predicted to range from 1.5 to 4.1 mg/kg.

Male reproductive organs might also be a target of toxicity. The effects on male fertility were described by (El- Makawy et al., 2006) at a low oral dose, corresponding to a human equivalent dose (HED) of 0.08 mg/kg in mice, which points to a negative safety margin compared to the intended clinical starting dose of 1.7 mg/kg. However, at the suggested efficacious dose, findings observed in repeated dose animal studies were considered reversible. Moreover, the design of the study did not follow standard recommendations. Reproductive and developmental studies revealed an impaired embryofetal development in rats and mice following oral treatments, characterized by decreased litter size and fetal weight, a decrease in number of live fetuses, a high incidence of total litter resorption, as well as an increased resorption index and a higher percentage of male fetuses in rats and mice following oral treatments during early pregnancy. According to Codd et al. (2015) effects on fertility in rats at HED doses of 4 mg/kg can be regarded as reversible.

Based on published information, the standard genotoxicity test battery as recommended by ICH M3 and ICH S2 including *in vitro* and *in vivo* genotoxicity tests, was negative.

The cumulative toxicology and pharmacokinetic data acquired from animal studies indicate that the nonclinical safety profile of oxfendazole supports the initiation of human studies.

1.1.2. Summary of clinical data

A Phase I single ascending dose (SAD) safety and PK study (NCT02234570) in 70 participants has been completed by ODG under the auspices to the NIAID (An et al., 2019). Oxfendazole was safe throughout the oral dose range of 0.5 – 60 mg/kg. A multiple ascending dose (MAD) Phase I safety and PK study and a food effect study (NCT03035760) has also been completed with daily doses up to 15 mg/kg for five days in 36 participants.

Following oral dose administration, the intestinal absorption of oxfendazole was rapid and plasma PK of oxfendazole of all participants showed a median T_{max} occurring after 2 hours. C_{max} increased from 944 ng/mL in the 0.5 mg/kg single dose group to 6,770 ng/mL in the 60 mg/kg single dose group and AUC increased from 11,700 ng*h/mL in the 0.5 mg/kg single dose group to 109,000 ng*h/mL in the 60 mg/kg single dose group. The increase in C_{max} and AUC with dose escalation was dose proportional up to 15 mg/kg and there was relatively little effect on C_{max} when the dose was increased to 30 or 60 mg/kg, indicating a nonlinear pharmacokinetics of oxfendazole beyond that dose level. In the multiple ascending dose study of orally administered oxfendazole, the C_{max} increased less than dose proportionally with increasing dose. C_{max} was 2960, 4160 and 5990 ng/mL in Group 1 (3 mg/kg), Group 2 (7.5 mg/kg), and Group 3 (15 mg/kg), respectively. $T_{1/2}$ was similar across all doses, with the means ranging from 8 to 12 hours. This suggests that the source of nonlinearity in the PK of oxfendazole is derived from its absorption phase, rather than the elimination phase. Accordingly, calculated apparent accumulation ratios at steady state were ranging from 0.970 to 1.27 over the 3 to 15 mg/kg multiple daily dose range. Like albendazole and other benzimidazole compounds, oxfendazole is a Biopharmaceutics Classification System (BCS) class II drug (i.e. drug with low solubility and high permeability). The observed nonlinear pharmacokinetics was therefore very likely due to its low water solubility. With an increase in dosage, the fraction of dose that was solubilized in gastrointestinal fluid and available to be

absorbed may be decreased, resulting in decreased bioavailability. At higher doses, the amount of oxfendazole may have reached its limit and consequently was no longer dependent on the dose given. When oxfendazole was administered after consumption of a fatty breakfast rather than after fasting, its bioavailability was higher. Both C_{max} (3010 vs 4500 ng/mL) and AUC (31600 vs 58900 ng*hr/mL) values were higher following the meal, and the T_{max} was prolonged from 1.99 to 8.90 hours. The $T_{1/2}$ values in the fasted and fed groups were similar, namely 7.65 and 8.05 hours.

There was no increase in unsolicited AEs with increasing dosage. A decline in hemoglobin was found in a few female participants over multiple days of dosing, however the absolute changes in hemoglobin over time were modest and very few participants had graded declines in hemoglobin. No changes were seen in white blood cells (WBC), neutrophil or platelet counts.

Oxfendazole underwent mild metabolism and the parent compound remained the main moiety in present in human plasma with little oxfendazole metabolized to the sulfone derivative. The metabolite profile of oxfendazole following its oral administration to healthy participants appears to be similar to that found in dog and pig rather than that of rats.

Oxfendazole was well tolerated at single doses up to 60 mg/kg and multiple daily doses of 15mg/kg for 5 days with no serious AEs or deaths. There was no statistically significant difference between the treatment and the placebo group in the proportion of participants experiencing AEs. There was no increase in the number of AEs with increasing dose. In the SAD study, a possible minor trend toward more gastrointestinal symptoms in the 30 and 60 mg/kg oxfendazole groups was observed (An et al., 2019). Adverse events were reported with a low frequency and without dose-dependency significance compared to placebo. In the food effect group, 3 out of 12 participants reported one or more AEs with no relationship to the fasting status. Similarly, there were no significant differences in the distribution of hematology, biochemistry or urine parameters between oxfendazole and placebo recipients. No changes were seen in WBC, neutrophil or platelet counts.

Evaluation of potential gender differences in oxfendazole pharmacokinetics and safety was only possible in the MAD study in which there were higher numbers of female participants than in the SAD study, with 3 female and 5 male participants in the 7.5 mg/kg and 4 female and 4 male participants in the 15 mg/kg dose group. C_{max}, C_{trough} and AUC were higher in female participants than in male participants. Since the terminal half-life was similar in both genders, the difference in pharmacokinetics is more likely a consequence of variations in bioavailability of oxfendazole, potentially related to gender-specific differences in gastric residence time or gastric pH levels. Comparison of safety parameters were similar in both genders, except hemoglobin. There was a minor trend toward a decline in hemoglobin with repeated administration of oxfendazole in female participants, which was more pronounced in the highest dose group. This finding was not clinically significant and was not paralleled by similar declines in other hematology parameters. The incidence of decreased hemoglobin values in females may be due to higher exposure of oxfendazole in female participants. The severity did not exceed grade 2 in any participants, and none of the cases of decreased hemoglobin were reported as AEs. The two female participants with moderate decreased hemoglobin during the study had low but still within normal range baseline hemoglobin levels. Furthermore, the overall number of female participants included in the study was small at 11 out of 36 participants.

In 2023, a randomized, placebo-controlled, double-blinded, single-center phase I study was conducted to evaluate the bioavailability of oxfendazole (tablet formulation) administered as a single dose and as multiple doses to assess safety in healthy volunteer participants.

Exposure to the investigational drug was greatest for oxfendazole followed by fenbendazole sulfone and fenbendazole. Within the limited dose range investigated, the PK of oxfendazole and its metabolites were less than dose proportional. The geometric mean apparent $t_{1/2}$ of oxfendazole was about 13 h and appeared to be independent of dose. The inter-participant variability was high for oxfendazole and moderate for the metabolites after single-dose administration, and decreased after multiple dosing.

Oxfendazole was overall well tolerated. There were no AEs reported in this study. There were no clinically significant abnormal results in laboratory parameters, vital signs, physical examinations, and ECG safety parameters over time. Within the limitations of this study, i.e. the absence of a positive QTc-prolonging control, small participant numbers, and only 2 doses of oxfendazole tested, the results from the present study do not indicate a QTc-prolonging effect of oxfendazole.

1.2. Clinical Trial Rationale

Global efforts to eliminate helminth infections or to provide individual care for patients are hampered by the absence of effective, affordable and accessible medicines and adapted field diagnostics. Diseases targeted and treatment limitations are depicted in Table 1.

Table 1: Description of the Diseases

Disease Pathogen	Pathology	Treatment & Limitations	Endemic in study site:
**Onchocerciasis** ***O. volvulus***	Blindness, severe dermatitis	Ivermectin, Moxidectin: do not kill the adult worms<br>Doxycycline: daily 5-6 week treatment to kill adult worms; children and pregnant women excluded	DRC, Cameroon
**Loiasis** ***L. loa***	Eye worm, angioedema, Calabar swelling,	DEC, Ivermectin: may cause life-threatening adverse events in patients with high mf load; daily 2-4 week treatment, currently not within mass drug administration (MDA) programs<br>Albendazole: limited efficacy against mf (twice daily 3-week treatment to reduce mf load)	DRC, Gabon, Cameroon
**Mansonellosis** ***M. perstans***	Often asymptomatic; subcutaneous oedema, pruritus, fever, joint pain, abdominal pain, pericarditis, pleuritis.	DEC, Ivermectin: MDA treatment (single dose) not efficacious; twice daily 3-week DEC or twice daily 4-week albendazole treatment reduces mf load, currently not within MDA programs	DRC, Gabon, Cameroon

**Trichiuriasis** ***T. trichiura***	Delayed child development, anemia	Albendazole, mebendazole, levamisole and pyrantel pamoate with poor efficacy at single dose	DRC, Gabon, Cameroon, Tanzania

DRC = Democratic Republic of Congo, DEC = Diethylcarbamazine

Onchocerciasis (river blindness) is a vector-transmitted tropical disease that mainly occurs in sub-Saharan Africa where approximately 21 million people are infected with *O. volvulus* and 218 million people live at risk of infection; among those, millions suffer from vision impairment and severe forms of dermatitis (Vinkeles Melchers et al., 2020). The most effective and safe drug used to treat onchocerciasis is ivermectin, resulting in near elimination of the filarial progeny, the microfilariae (mf), from the skin and temporary prevention of new mf release by the adult worms (Basáñez et al., 2008). However, adult worms are not killed and mf return and reach levels able to sustain transmission of the infection within 6-8 months; requiring repeated mass drug administration (MDA) over many years. This has been a successful strategy to lower disease burden, but after three decades of MDA, elimination has only been achieved in a few countries and it is clear that additional tools are needed. In addition, many areas co-endemic with the filaria *L. loa* do not receive MDA due to the risk of life-threatening adverse events in patients with high *L. loa* mf loads (Boussinesq et al., 2003). Furthermore, areas with suboptimal ivermectin responses have been described.

Soil-transmitted helminth (STH) infections are caused by the large roundworm *A. lumbricoides*, the hookworms *Necator americanus* and *Ancylostoma duodenale*, the whipworm *T. trichiura*, and *Strongyloides stercoralis*. An estimated 5.3 billion people are at risk, while 1.5 billion are infected with at least one of the STHs (Jourdan et al., 2018). Disease burden results from disability, influencing school attendance, child development and overall economic productivity. There is no existing pipeline for the development of novel drugs for STH infections, and thus the current treatment relies on less than a handful of drugs registered for treatment and control of human STH infections, all of which have limitations. Albendazole, mebendazole, levamisole and pyrantel pamoate are currently on the WHO list of essential medicines for the treatment of STH infections. The two benzimidazoles albendazole and mebendazole are widely used in preventive chemotherapy programs against STH infections. Although albendazole and mebendazole are highly effective against *A. lumbricoides*, they are poorly efficacious against *T. trichiura* at single doses (Moser et al., 2017). For all drugs, there is an enhanced risk of drug resistance development, as is already well described for STH infections in livestock (Wolstenholme et al., 2004).

The WHO recently published three critical actions to achieve the targeted elimination (interruption of transmission) for onchocerciasis, 1) start MDA in all endemic areas after mapping and implement new strategies where appropriate, 2) develop improved diagnostics to facilitate mapping and decisions to eliminate transmission, and 3) develop a macrofilaricide (adult worm killing drug) to accelerate interruption of transmission.

For STH, a multisectoral action for deworming programs is also suggested by the WHO Road Map 2021-2030. Hence, to control and eliminate both gastrointestinal (STH) and tissue-dwelling (filariae) nematode infections, adaptive clinical trial platforms to test and compare new drug candidates are needed to provide new drug candidates that effectively (i.e. to 90–100%) eliminate adult worms or render them permanently sterile (Hotez et al., 2008). These can either be administered in mass administration or after targeting the remaining infective population using a "test & treat" approach.

Albeit the presence of disease symptoms, *L. loa* infection itself has not been taken up in the global health agenda due to limited financial resources and focus on other global health challenges. Loiasis however does cause pruritus, oedema, Calabar swelling, and adult worms migrate through

the subcutaneous tissue & eye in the infected patient. A recent systemic review indicated that atypical symptoms, including respiratory, cardiac, neurological, gastrointestinal, renal and ophtalmological pathologies, were reported in almost half of the case reports on loiasis (Buell et al., 2019). Thus, loiasis should be considered as a significant public health problem. The current treatment of loiasis requires several weeks of ivermectin or DEC treatment and may require additional weeks of initial albendazole treatment in patients with high mf counts. Due to potential life-threatening adverse events in patients with high mf counts, treatments have to be provided under hospital surveillance.

For infections with *M. perstans* no MDAs are installed, and the current treatment regimen used for onchocerciasis MDA (single combination with ivermectin plus albendazole) has little to no impact on *M. perstans* microfilaremia and therefore transmission of the infection. Thus, this infection is common in tropical countries with an estimated 114 million people being infected, mostly asymptomatic. However, there is evidence that *M. perstans* infection impacts co-infections with *M. tuberculosis* and HIV.

Therefore, drugs are required for *O. volvulus, L. loa, M. perstans,* and STH infections. Candidates should be highly efficacious or even curative, kill or permanently sterilize the adult nematodes, that may be administered in mass administration or by targeting the remaining infective population via a "test & treat" approach. A drug acting safely on onchocerciasis, loiasis, mansonellosis and trichuriasis would be a major leap towards the WHO roadmap goals.

1.3. Benefit/Risk Assessment

As for any experimental drug, there may be side effects of oxfendazole that are not presently known. No serious adverse reactions (SAR) occurred in the SAD and MAD study conducted by ODG (NCT02234570 and NCT03035760) or the relative Bioavailability study conducted by Swiss TPH (NCT04920292). Based on the data from these studies in healthy volunteers, oxfendazole was safe and well tolerated at doses up to 60 mg/kg and showed acceptable safety and tolerability profiles after five repeated daily doses of up to 15 mg/kg, with a few reports of non-serious AEs in the two studies. There was no increase in the number of AEs with increasing dose. No serious adverse events or deaths were reported.

In the non-clinical and Phase-I-data currently available, liver, bone marrow and reproductive organs have been identified as target organs. Based on this, special attention is given to the identified risk of drug-food effect, potential risk of hepatotoxicity, potential risk of bone marrow toxicity, potential risk of male fertility effects and potential risk of teratogenic effects, as well as potential risk of drug-drug interactions (see Summary of Data and Guidance to the Investigator, Investigator Brochure).

Identified and potential risks in humans are also based on the class effect of benzimidazoles, including lab abnormalities such as mild to moderate elevations of liver enzymes that normalize after treatment.

Based on the limited data in humans and the early stage of the clinical development, none of the proposed potential risks and identified risks after administration of oxfendazole in humans are currently considered as important risks.

Nevertheless, to minimize risks:

Participants will be closely monitored by history, physical examination, and laboratory tests including liver function tests and full blood cell counts to assess safety and tolerability.

Pregnant and breast-feeding women will not be eligible for inclusion in the trial, and highly effective contraception methods will be required as inclusion criteria for WOCBP (see Appendix 4) . Male participants will be required to use condoms and refrain from donating sperm.

Oxfendazole has excellent adult worm killing efficacy in livestock and companion animals as well as in rodent filarial models. Consequently, it is anticipated that the benefit for participants is the potential development of an antiparasitic treatment for soil-transmitted helminths and tissue parasites.

2. Objectives, Endpoints, and Estimands

2.1. Summary

Primary objective and endpoint:

The primary objective is to identify a safe and efficient dose of oxfendazole for each of the four indications: participants infected with one or more of the following: *Onchocerca volvulus* (onchocerciasis), *Loa loa* (loiasis), *Mansonella perstans* (mansonellosis) or *Trichuris trichiura* (trichuriasis).

The associated endpoint is parasite load at day 18 (egg count for trichuriasis) and/or month 12 (mf count for all other indications). See sections 2.2 and 2.3 for the estimands.

Secondary objectives and endpoints:

The secondary objectives are:

1. To demonstrate the safety, tolerability, characteristics of oxfendazole in helminth infected individuals. The associated endpoint is frequency of adverse events (AEs), serious adverse events (SAEs) and discontinuations or temporary suspensions of IP up to day 18 (and day 45 for Arms 10, 11) and to the end of the trial. Estimands are to be developed in SAP.
2. To demonstrate pharmacokinetics characteristics of oxfendazole in helminth infected individuals. The associated endpoint is PK parameters (AUC0-t, AUC0-∞, AUCtau, Cmax, Tmax, $t_{1/2}$ and Racc) of oxfendazole. Estimands are to be developed in SAP.
3. To demonstrate efficacy characteristics of different oxfendazole dose regimens in helminth infected individuals. The associated endpoints are:

Onchocerca volvulus infection

- Relative reduction of skin mf at day 18, (and day 45 for arms 10, 11), month 3, 6, 9 and 12
- Absence of live female adult worms with normal embryogenesis (assessed by histological examination of nodules collected at month 12)
- Absence of skin mf at day 18, (and day 45 for arms 10, 11), month 3, 6, 9 and 12
- Absence of signs and symptoms of onchocerciasis day 18 (and day 45 for Arms 10, 11), month 3, 6, 9 and 12

Loa loa infection

- Relative reduction of *L. loa* blood mf at day 18, (and day 45 for arms 10, 11), month 3, 6, 9 and 12
- Area under the curve of *L. loa* blood mf during follow-up period for each dose level
- Absence of *L. loa* blood mf at day 18, (and day 45 for arms 10, 11), month 3, 6, 9 and 12

Absence of signs and symptoms due to eye worm migration, Calabar swelling, and dermal migration at day 18, (and day 45 for arms 10, 11), month 3, 6, 9 and 12

Mansonella perstans infection

- Relative reduction of *M. perstans* blood mf at day 18, (and day 45 for arms 10, 11), month 3, 6, 9 and 12
- Area under the curve of *M. perstans* blood mf during follow-up period for each dose level
- Absence of *M. perstans* blood mf at day 18, (and day 45 for arms 10, 11), month 3, 6, 9 and 12
- Absence of signs and symptoms of pruritus, urticaria and oedema at day 18 (and day 45 for arms 10, 11), month 3, 6, 9 and 12

Trichuris trichiura infection

- Relative reduction of eggs (egg reduction rate) at day 18 (assessed by quadruplicate Kato-Katz)
- Absence of eggs (cure rate) at day 18 (assessed by quadruplicate Kato-Katz)
- Relative reduction of eggs (egg reduction rate) at day 18 (assessed by quadruplicate Kato-Katz) for additional soil-transmitted helminth (STH) infections (*Ascaris lumbricoides* and hookworm)
- Absence of eggs (cure rate) at day 18 (assessed by quadruplicate Kato-Katz) for additional STH infections (*Ascaris lumbricoides* and hookworm)

<u>Exploratory objectives:</u>

1. Facultative subgroup analysis to demonstrate efficacy characteristics of oxfendazole in co-infection with other helminth infections. The associated endpoints are:

- Reduction of eggs at day 18 (for trichuriasis) and mf in blood and skin at month 12 for other parasites
- Absence of eggs at day 18 (for trichuriasis) and mf in blood and skin at month 12 for other parasites

2. Assessment of parasite DNA in the samples at day 18 (trichuriasis) and month 12 (for all other infections) (PCR and loop-mediated isothermal amplification (LAMP)) for helminth infections, from skin snip, blood, and stool samples.
   - o Positivity or negativity in samples
   - o Correlation of PCR/LAMP results with those measured by the standard microscopy diagnostic methods

2.2. Primary Estimands

<u>Primary estimand:</u>

The primary clinical question of interest is for each considered dose: What is the intervention difference in mf or eggs counts at 12 months /18 days respectively in participants with onchocerciasis, loiasis, mansonellosis, /trichuriasis, a) regardless of intervention discontinuation, b) regardless of non-compliance to study treatment or background medication and c) under the assumption that no rescue medication would be used and no patients die.

The primary estimand is described by the following attributes:

- Treatment: Oxfendazole,3 different regimens for onchocerciasis, loiasis, mansonellosis, and trichuriasis and Placebo
- Population: Participants with onchocerciasis, mansonellosis, loiasis/trichuriasis as described by the inclusion and exclusion criteria with at least 1 dose of study drug and day 18/month 12 result for the endpoint.
- Endpoint: Parasite load at month 12 (in mf) and/or at day 18 (in egg counts)
- Intercurrent Events (ICE):
    - ICE 1: Treatment discontinuation, non-compliance.
        - a) A treatment policy strategy will be used to estimate treatment effect, regardless of treatment discontinuation (e.g., discontinuation by the investigator if he/she judges that treatment is no longer appropriate, if the participant's clinical condition is worsening, or for AE).
        - b) Regardless of non-compliance to the study treatment and/or background medication or discontinuation or change in dose regimen of study treatment and/or background medication.
    - ICE 2: death, rescue medication. A hypothetical strategy will be used to estimate what the treatment effect would have been if participants did not die or receive rescue medication. In particular, a worst outcome scenario will be assumed, where the highest possible rank in the primary outcome variable is assigned to participants who died or received rescue medication.

- Population-level summary: probabilistic index of parasite load between Placebo and intervention conditions at month 12 or day 18 (trichuriasis)
- Rationale for estimand: The clinically most relevant estimand was chosen.

2.3. Secondary Estimand(s)

Secondary estimand: The primary clinical question of interest is: What is the intervention difference in mf or eggs counts at 12 months /18 days respectively in participants with onchocerciasis, mansonellosis, loiasis/trichuriasis a) without any major protocol violations including the violation of entry criteria, b) who completed the treatment regimen.

The secondary estimand is described by the following attributes:

- Treatment: Oxfendazole, 3 different regimens for onchocerciasis, loiasis, mansonellosis, and trichuriasis and Placebo
- Population: participants with onchocerciasis, mansonellosis, loiasis/trichuriasis as described by the inclusion and exclusion criteria.
- Endpoint: Parasite load at month 12 (in mf) and/or at day 18 (in egg counts)
- Intercurrent Events (ICE)
    - ICE 1: treatment discontinuation, non-compliance. A hypothetical strategy will be used to estimate treatment effect,
        - a) without any major protocol violations including the violation of entry criteria

b) for completed preplanned treatment regimen

where the measurement of all primary variables are available.

- ICE 2: death unrelated to treatment and disease

A hypothetical strategy will be used to estimate what the treatment effect would have been if participants did not die. It is assumed that the death is unrelated to treatment and the disease such that the outcome distribution of surviving participants is the same as for participants dying unrelated to treatment and disease.

- ICE 3: rescue medication, death related to treatment and disease

A hypothetical strategy will be used to estimate what the treatment effect would have been if participants did not die or receive rescue medication. In particular, a worst outcome scenario will be assumed, where the highest possible rank in the primary outcome variable is assigned to participants who died or received rescue medication.

- Population-level summary: probabilistic index of parasite load between intervention conditions and Placebo at month 12 (filariae) or day 18 (Trichuris)
- Rationale for estimand: Analysis of the primary endpoint for per protocol population.

The secondary estimands for the secondary endpoints will have the same treatment, population and dealing with intercurrent events as the primary endpoint, except for the absence of signs and symptoms endpoints for which the population are patients that received at least 1 dose of study drug. These secondary estimands will be described in the SAP.

2.4. Exploratory Estimand(s)

Not applicable

3. Clinical Trial Design

3.1. Overall Design

This study will be a controlled, randomised, adaptive, double-blinded parallel group study. For each of the diseases, the trial will initially consist of three treatment arms including placebo control.

***T. trichiura* :**
This part will consist of one stage,160 participants will be allocated (1:1:1:1) to the placebo or dose groups:

Arm 0: Three days, once daily, 800 mg oxfendazole (40 participants)
Arm 1: One day, single dose, 400 mg oxfendazole (40 participants)
Arm 2: One day, single dose, 800 mg oxfendazole (40 participants)
Arm 3: One day, single dose, matching placebo (40 participants)
)

O. volvulus, L. loa and M. perstans infections:
This part will consist of two stages.
In the first stage, about a total of 360 (120 per disease) participants will be allocated (1:1:1) to the placebo or dose groups:

Arm 4: Five days, once daily, 400 mg, oxfendazole (40 participants per disease)
Arm 5: Five days, once daily, 800 mg, oxfendazole (40 participants per disease)
Arm 6: Five days, once daily, matching placebo (40 participants per disease)

Study recruitment will be halted as soon as the pre-planned number of first stage participants per group is reached. When all first stage participants have reached the 6 months visit, an interim analysis will be conducted in each of the diseases and the trial may be adapted. Especially, apparently inefficacious or unsafe doses may be dropped. If no dose response signal is observed in the interim analyses, a repeated treatment arm may be added. The trial continues to the second stage where up to 240 (80 per disease) additional participants will be allocated to the selected doses and the placebo group. Furthermore, the sample sizes may be reassessed. While efficacy will be evaluated independently in each disease, safety data from all diseases will inform the safety monitoring of the trial.

In the second stage, about a total of 240 (80 per disease) participants will be allocated (1:1 (if only arm 10 is selected) or 1:1:1 (Arm 9 : Arm 7: Arm 8, if Arms 7 and 8 are selected) and 1:1:2:2) (Arm 9: Arm 11: Arm 8 : Arm 10, if arms 8 and 10 are selected)) to the placebo or selected dose groups, among the following:

Arm 7: Five days, once daily, 400 mg, oxfendazole
Arm 8: Five days, once daily, 800 mg, oxfendazole
Arm 9: Matching placebo to Arm 7, 8
Arm 10: Repeated dose. Five days, once daily, month 1 and month 2, 800 mg, oxfendazole
Arm 11: Matching placebo to Arm 10

A participant will be considered enrolled once the informed consent form has been signed and randomization to an assigned treatment has occurred. Following enrolment, IP administration will begin according to the study group assignment.
Participants will undergo regular clinical and laboratory assessments for safety and efficacy according to the study Schedule of Activities.

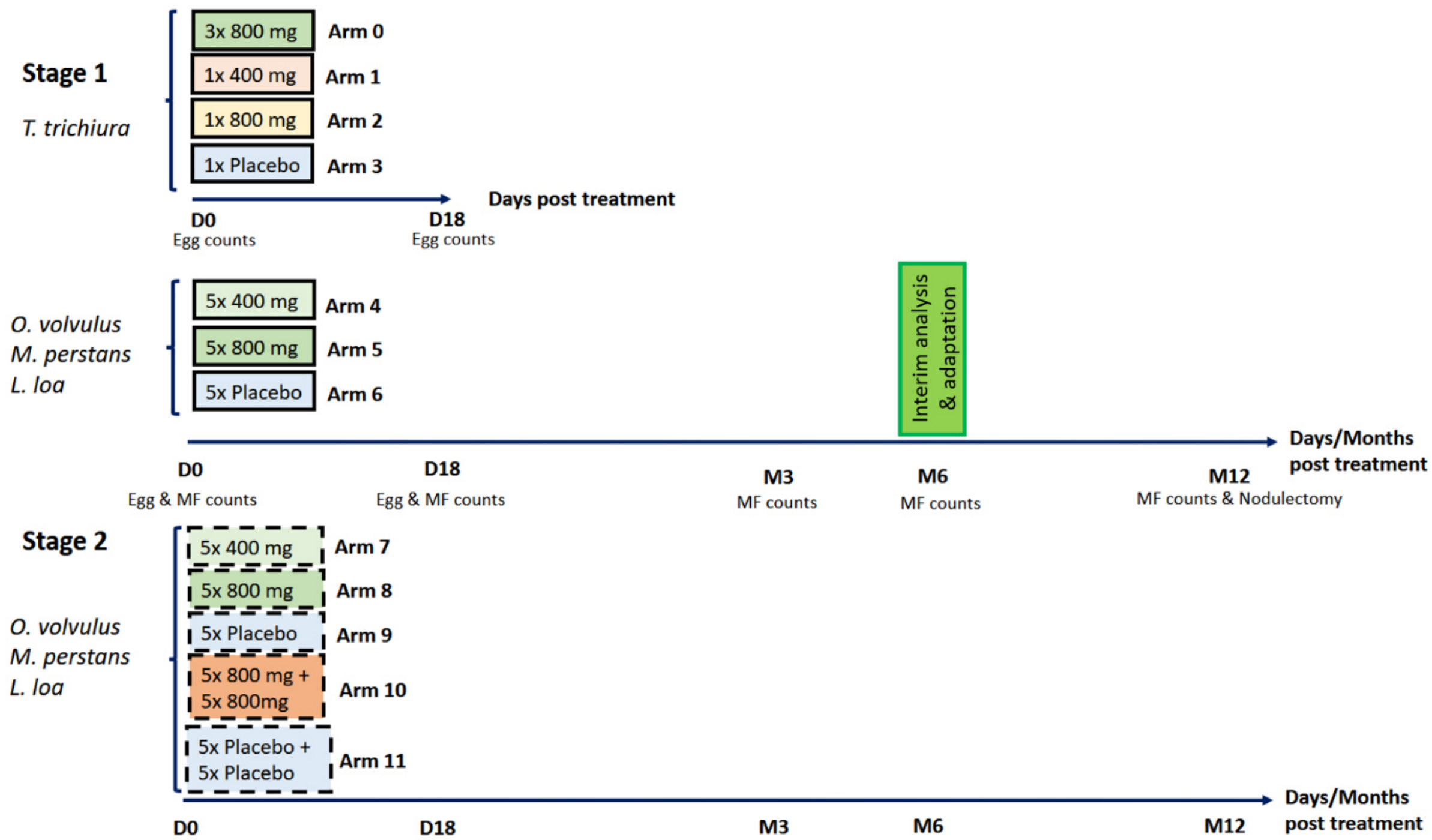

Figure 1: Study Design Overview

3.2. Scientific Rationale for Clinical Trial Design

This study is an adaptive, basket Phase IIa trial that allows the simultaneous evaluation of multiple doses of the experimental treatment in multiple diseases within the same trial structure and will be able to answer efficiently multiple questions in less time. A lack of available compounds for the treatment of helminth infections to be tested in humans exists and no suitable biomarkers have been developed that enable an early detection of drug effects in these mainly chronic infections. Applied the first time in NTDs, an adaptive, collaborative, multi-arm basket trial allows the simultaneous evaluation of multiple diseases and multiple doses within the same clinical trial structure based on a master protocol.

Patient-centered design innovation: This design reduces the need for redundant clinical trials and allows the use of shared placebo groups within each disease to test multiple doses. It will thereby decrease the probability of participants being randomized to a placebo group and increase the probability of a participant in the clinical trial receiving the most promising treatment for their disease. Furthermore, patients in sub-Saharan Africa often have acquired multiple parasite

infections, and either participants will then be excluded or only data related to the indication will be used. Here, we aim to randomize per disease and in the case of coinfections all data will feed into the data analysis. This further maximizes the study output and minimizes the number of participants and costs.

Operational innovation: The structure of a master protocol will include a shared infrastructure including joint database and case report form (CRF). This will allow partners to share the infrastructure and test clinical hypotheses in parallel. A master protocol further offers a patient centric approach by allowing for multiple infections, as patients are very often infected with more than one helminth species. A joint setup and use of such a protocol will harmonize clinical trial setup and conduct, further increasing the quality, comparability and capacity build throughout consortium partners.

Study rationale: Onchocerciasis:

Onchocerciasis represents a major public health problem in affected countries with up to 1.34 million disability adjusted life years (DALYs) lost in 2017 (Kyu et al., 2018). This disproportionately high number is driven by disease-induced disability, and overall loss of economic productivity.

The aim of MDA programs is to eliminate transmission of the parasites by periodic treatment of vulnerable populations with ivermectin. This removes mf from the skin and temporarily prevents the release of mf by the adult female worms. Ivermectin, the current treatment for onchocerciasis used in MDA programs, is only microfilaricidal, killing only the larvae (mf) but not the adult worms and mf return within 6-8 months. Although ivermectin is effective in reducing transmission cycles, treatment must be repeated at regular intervals of 6 or 12 months for 10-15 years (the reproductive life span of the adult worms), making implementation of a sustainable MDA program challenging and extremely difficult in some endemic countries. In addition, there are limitations with the use of ivermectin in areas where *Loa loa* is co-endemic. Patients with *L. loa* infections can suffer from high mf loads and when treated with a microfilaricidal drug, such as ivermectin, they are at risk of serious adverse events. Therefore, areas with coinfections are currently excluded from MDA programs and remain without treatment.

Recent modelling using ONCHOSIM predicts there will be around 10 million infected individuals with positive skin mf in 2025, with >6 million manifesting clinical symptoms (Vinkeles Melchers et al., 2020). The total population at risk will be approximately 200 million. It has become clear that the success of elimination programs is highly dependent on local pre-control endemicity, past and future (therapeutic) coverage patterns, and geographic coverage. Notably, MDA requires extensive logistical efforts and financial commitments, as it is dependent on the sustained coordination of supply logistics to ensure that treatment reaches all target recipients.

Because of these challenges, interruption of transmission and disease elimination worldwide has proven more difficult than initially projected. In addition, if given for long periods, MDA carries the risk of resistance, already described for ivermectin use in animal-health applications, together with sub-optimal response in humans (Osei-Atweneboana et al., 2011). A number of studies and simulations (Kim et al., 2017; Verver et al., 2018; Walker et al., 2017) indicate that elimination of onchocerciasis with the current strategies will not be feasible within the timelines of the UN Sustainable Development Goals (World Health Organization, 2017).

Clearly, additional treatment options are needed to improve clinical care for individual patients, reduce human suffering, achieve cure and ensure advancement toward the WHO's goal of elimination by providing additional tools to shorten the timelines for elimination.
While MDA is effective in reducing transmission and morbidity in some settings, a drug that kills the adult worm or has a long-term sterilizing effect, offers the advantage of reducing the need for multiple cycles of MDA and would be an essential addition to the global strategy to eliminate onchocerciasis.

Study rationale: Loiasis:
The filarial helminth *Loa loa* and its characteristic manifestations of eye worm migration and Calabar swelling have been described in the literature for centuries (Boussinesq, 2006; Metzger & Mordmüller, 2014). Currently it is estimated that more than 10 million people are infected with *L. loa* in central Africa and parts of western Africa, including highly endemic regions with historic eye worm prevalence of more than 60% (Metzger & Mordmüller, 2014). *L. loa* is transmitted by diurnal tabanid *Chrysops* spp flies and can cause extremely high blood microfilaria counts in the human host. During MDA programmes, these hypermicrofilaraemic patients were found to be at risk of developing serious adverse events when treated with certain anthelmintic drugs (Gardon et al., 1997). Hence, co-endemicity with loiasis hinders elimination programmes for onchocerciasis or lymphatic filariasis, and most loiasis research in the past three decades aimed to overcome this difficulty (Kelly-Hope et al., 2018). However, loiasis itself has attracted little interest for clinical research or control programmes. The few existing reports on the clinical syndrome caused by *L. loa* in endemic populations indicate substantial morbidity with this infection (Kelly-Hope et al., 2018). Furthermore, severe organ damage has been attributed to loiasis, although thought to be rare (Buell et al., 2019), and loiasis was described to be a major reason for medical consultation in endemic areas (Buell et al., 2019). Importantly, a recent retrospective study revealed excess mortality in loiasis patients with high microfilariae loads (Chesnais et al., 2017). Even with this growing evidence for the severity of the disease, loiasis remains so neglected that it is not even listed as a neglected tropical disease by WHO, and there remains a lack of funding for research, and an absence of treatment and control programmes for the disease in affected countries. Thus, drugs that also kill adult *L. loa* parasites would be a leap ahead for global health.

Study rationale: Mansonellosis:
Human mansonellosis is caused by *M. perstans, M. ozzardi* and *M. streptocerca*, the three main filarial species in the genus *Mansonella*. A potentially new species was described in Gabon, which is called *Mansonella* spec. DEUX. Despite accumulating evidence of a high prevalence in endemic areas, there is currently no filariasis control programme targeting mansonellosis and treatments currently used for onchocerciasis MDA (single dose ivermectin and albendazole) have no or only minor impact on *M. perstans* microfilaremia. The health-related impact on people living with these filariae remains unknown, and evidence regarding treatment strategies is scarce. Like other neglected diseases, it mainly affects poor populations living in tropical and subtropical climates. Mansonellosis can be considered one of the most NTDs. In-depth clinical studies aiming to describe the symptoms associated with mansonellosis are lacking. Most microfilaremic individuals are reported to be asymptomatic; eosinophilia is a common feature. Nonspecific symptoms, including pruritus, urticaria, arthralgia, abdominal pain and fatigue, have been described. In *M. perstans*, symptoms of infection are probably related to the migration of the worms and include

transient subcutaneous swellings (similar to the Calabar swellings caused by *L. loa*), pericarditis, pleuritis and inflammatory granulomatous nodules surrounding dead adult worms (Simonsen et al., 2011). The symptoms associated with *M. streptocerca* infection are similar to onchocerciasis, with the following characteristics: papular dermatitis and pruritus predominates on the upper parts of the body, and the absence of nodules. It is clear that (a) the data on morbidity, mortality and clinical picture of mansonellosis remain scarce, (b) the limited data on vector transmission hinder the development of effective control strategies, and (c) effective treatment is lacking. Given such inattentiveness, a drug proven effective and implemented for other helminth diseases, could be a game changer for mansonelloses.

Study rationale: STH:
Soil-transmitted helminthiasis belongs to the NTDs and is caused by infections with the roundworm *Ascaris lumbricoides*, the hookworms (*Necator americanus*, *Ancylostoma duodenale* and *Ancylostoma ceylanicum*) or the whipworm *Trichuris trichiura*. Among all NTDs, soil-transmitted helminthiasis affects the largest number of people with an estimated 1.5 billion infected individuals and causes the highest disease burden. Although the global STH prevalence decreased after almost two decades of helminth control activities, there are still more than 100 countries in need for control programs (i.e. infection prevalence > 20%). Albendazole and mebendazole are the most widely used drugs for preventive chemotherapy campaigns against STH infections. Albendazole is characterized by high cure rates (CRs) against infections with *A. lumbricoides* (96%) and moderate CRs against hookworm infections (80%). Both benzimidazoles show low efficacy against *T. trichiura* (31%). The benzimidazole oxfendazole could serve as an alternative treatment and should be evaluated for its efficacy against STH infections.

3.3. Justification for Dose

For filarial infections, the absence of animal models using human parasites, except for Lymphatic filariasis, presents a challenge. *Litomosoides sigmodontis* is commonly employed to infect rodents to assess the efficacy of filarial infection treatments. Such animal models provide a straightforward means to predict the efficacy of novel treatments, evaluate the pharmacokinetic/pharmacodynamic (PK/PD) relationship, and make predictions concerning potential clinically efficacious regimens.

Oxfendazole underwent extensive evaluation in various preclinical models using *L. sigmodontis* with diverse dose regimens and routes of administration. A dose-proportional effect was demonstrated, achieving sterile cure following oral administration of oxfendazole at doses of 25 mg/kg (and above) for 10 to 5 consecutive days. Via the subcutaneous route, a dose of 1 mg/kg administered for 5 consecutive days resulted in a reduction of almost 100% of adult worms in infected animals (Hübner et al., 2020).

The selection of the starting dose for the Single Ascending Dose (SAD) study was informed by the No Observed Adverse Effect Level (NOAEL) derived from Good Laboratory Practice (GLP) toxicology studies conducted in rats (Codd et al., 2015). The determined NOAEL led to defining the SAD study's initial dose at 0.5 mg/kg (Bach et al., 2021). Subsequent dose escalation in healthy volunteers reached 60 mg/kg in the SAD study and 15 mg/kg in the Multiple Ascending Dose

(MAD) study (Bach et al., 2020), resulting in respective Area Under the Curve (AUC) values of 110,000 and 89,300 ng h/ml.

In the SAD study, oxfendazole suspension was administered across the full planned dose range (seven escalating doses from 0.5 to 60 mg/kg), as no serious AEs and few moderate or mild AEs were observed. A total of 12 AEs was observed: none were graded as serious, 10 were graded as mild and two were graded as moderate. The 12 AEs observed occurred in seven participants, 6 (out of 56) receiving OXF and 1 (out of 14) receiving placebo. There was no significant difference in the proportion of AEs occurring in OXF as compared to placebo receiving participants. In the MAD study, oxfendazole was well tolerated at all dose levels studied (three escalating doses from 3 to 15 mg/kg). There were no severe (Grade 3) unsolicited AEs reported, nor were there any SAEs. Among the 36 participants, 11 unsolicited AEs were reported with none being considered related to the study product. Only one of the AEs was considered of moderate severity, and all of the remaining events were characterized as mild. There were no significant trends in tolerability related to increasing dose of oxfendazole. There was no increase in unsolicited AEs with increasing dose. Two women showed a moderate decline in hemoglobin over multiple days of dosing (15 mg/kg), but started the study with low normal hemoglobin values. No changes were seen in WBC, neutrophil or platelet counts. In this study a fixed dose will be used and reflects the following dosing:

Table 2 Dosing illustration for the fixed doses used in OXF-01

**Weight (kg)**	**Oxfendazole 400 mg**	**Oxfendazole 800 mg**
**45**	8.9 mg/kg	17.8 mg/kg
**50**	8.0 mg/kg	16.0 mg/kg
**55**	7.3 mg/kg	14.5 mg/kg
**60**	6.7 mg/kg	13.3 mg/kg
**65**	6.2 mg/kg	12.3 mg/kg

Considering the preclinical efficacy data, the key determinant of efficacy is the maintenance of plasma levels above approximately 100 ng/mL over five days (based on subcutaneous treatment at 1 mg/kg in mice) for filarial diseases (Hübner et al., 2020). A population pharmacokinetic modelling of plasma concentrations of oxfendazole and its metabolites were analysed using nonlinear mixed effects modelling in NONMEM (v7.4, Icon Development Solution, Ellicott City, MD, USA). Plasma concentrations were converted into molar units and transformed logarithmically before analysis. None of the PK samples for oxfendazole and oxfendazole sulfone were below the LLOQ, while 25.3% of fenbendazole samples were below the LLOQ, predominantly in the formation phase. A first-order conditional estimation method with interactions (FOCE-I) was used for model development, facilitated by Pirana, PsN, R, and TIBCO Spotfire. Model selection relied on objective function values (OFV), with a decrease of at least 3.84 indicating significant improvement. A drug-metabolites model was developed, assuming 50% conversion of oxfendazole to fenbendazole and oxfendazole sulfone clearance each. One- and two-compartment models were evaluated, along with different absorption models. Covariates such as body weight, dose, sex, and age were explored, with body weight standardized to 70 kg. Dose-

limited bioavailability was implemented in the final model, with model evaluation conducted through goodness-of-fit diagnostics, visual predictive checks, and bootstrapping.

This population pharmacokinetic modelling predicts that a dose ranging from 400 mg to 800 mg for 5 consecutive days is expected to achieve these plasma levels, thus establishing efficacy in humans in 92% and 97% of treated participants respectively. This is illustrated in the table below.

Table 3 Percentage of participants achieving target concentrations (tissue nematodes) for a minimum of 1-5 days

Percentage of subjects achieving target concentrations for a minimum of 1 – 5 days

Minimum days above target	Scenario 1: 100mg SD	Scenario 2: 400mg SD	Scenario 3: 800mg SD	Scenario 4: 100mg 5days QD	Scenario 5: 400mg 5days QD	Scenario 6: 800mg 5days QD
1	63.8	87.9	94.0	98.0	99.6	100
2	11.5	32.1	44.8	95.5	99.4	99.9
3	0.5	3.5	7.0	92.2	98.9	99.6
4	0	0.3	0.5	85.1	97.3	99.1
5	0	0	0	70.5	92.2	97.1

Threshold set at 90% of patients above the minimum number of days

In addition to predicting the attainment of efficacious drug levels, population pharmacokinetic modelling was employed to ensure that proposed dosing regimens of oxfendazole do not surpass established safe levels identified in phase 1 SAD and MAD studies conducted in healthy volunteers (Bach et al., 2021; Codd et al., 2015). These studies demonstrated the safety of a single dose of 60 mg/kg of oxfendazole administered as a suspension, with no significant side effects reported. Similarly, a dose of 15 mg/kg administered for 5 consecutive days was deemed safe, with no notable side effects. Population pharmacokinetic modelling simulated the administration of oxfendazole tablets to 1000 patients, predicting the drug levels attained following various dosing regimens. Figure 2 depicts scenarios involving single doses of 100, 400, and 800 mg of oxfendazole tablets. The simulations indicate that for a single dose of 100 mg, no patients are expected to exceed the safe drug levels observed in the previous SAD study. For a 400 mg dose, only 0.7% of patients are predicted to exceed levels from this trial, while for an 800 mg dose, the proportion rises to 2.5%. Similarly, multiple dosing regimens involving 5 consecutive doses of 100, 400, and 800 mg were simulated and compared to safe drug levels reached at 15 mg/kg for 5 consecutive days. The simulation indicates 0.4% patients are predicted to exceed these levels for a 100 mg dose, 2.5% for a 400 mg dose and 6.1% for 800 mg dose. Given that the highest tested

doses in phase 1 studies (SAD and MAD) were safe with no notable adverse effects, the risk associated with overdosing is relatively limited and manageable within the scope of this clinical trial.

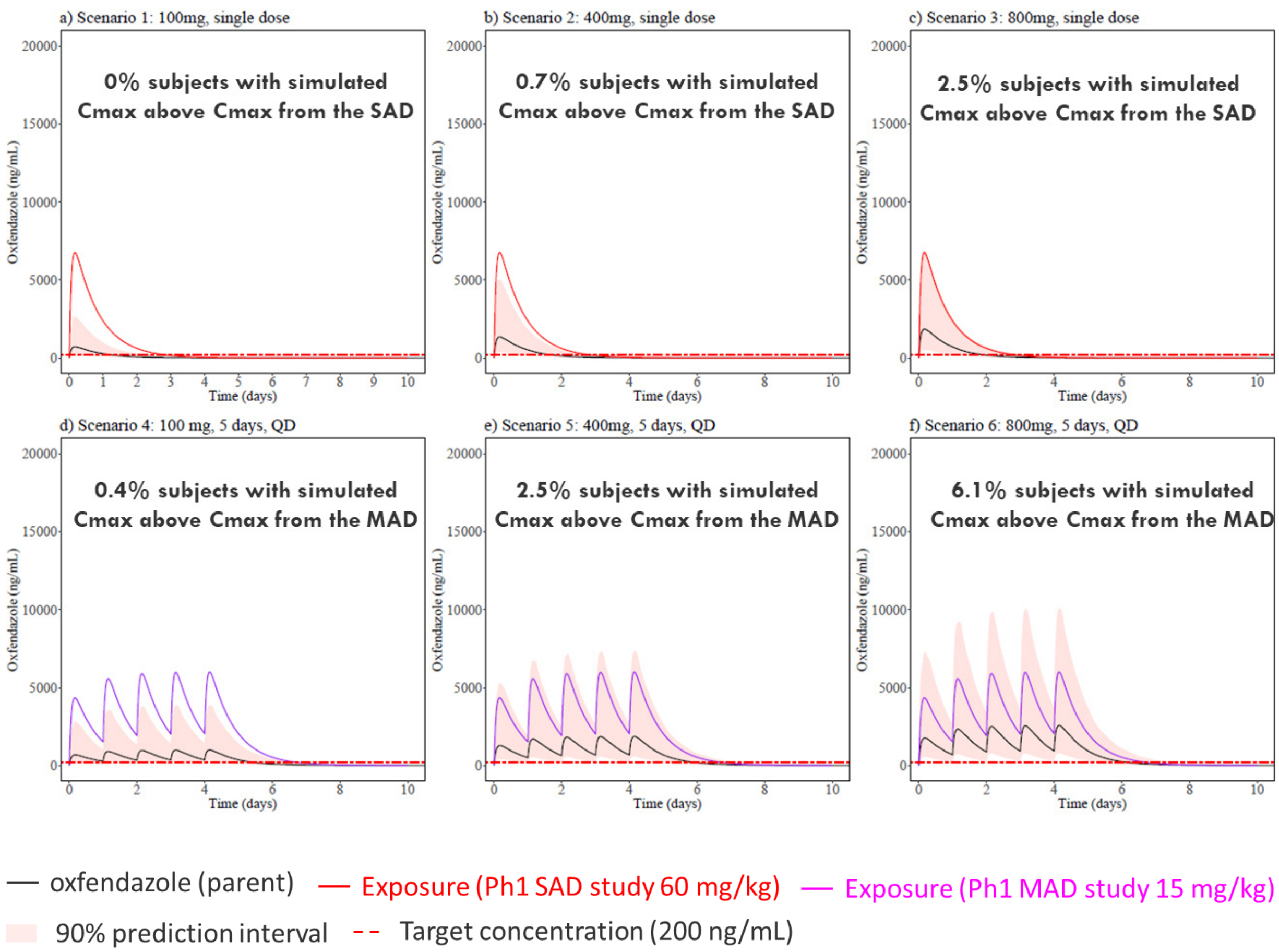

Figure 2: Dosing scenarios with 100, 400, and 800 mg oxfendazole

3.4. End-of-Clinical Trial Definition

The overall end of the clinical trial is defined as the date of the last visit of the last participant in the clinical trial globally. After all participants have completed the clinical phase of the trial (Last Patient Last Visit), activities required for trial analysis and reporting will start.

A participant is considered to have completed the clinical trial if:

- the participant has completed all periods of the clinical trial including the last visit, or
- the participant has completed the last scheduled procedure shown in the SoA.

The end of clinical trial for an individual participant is his or her "Last Visit" defined as whichever of the following events occurs last:

- screening failure, or
- the last clinical trial visit (shown in SoA), or
- early discontinuation visit, or
- the last visit the participant attended for participants lost to follow-up or withdrawn.

4. Clinical Trial Population

Prospective approval of protocol deviations to recruitment and enrollment criteria, also known as protocol waivers or exemptions, is not permitted.

4.1. Inclusion Criteria

Participants are eligible to be included in the clinical trial only if all of the following criteria apply:

1. Participant must be 18 or the legal age of consent in the jurisdiction in which the clinical trial is taking place to 65 years of age inclusive, at the time of signing the informed consent.
2. A diagnosis of one or more of the following: trichuriasis, mansonellosis, onchocerciasis and loiasis confirmed within 3 months before randomization.
3. Body weight at screening ≥ 45 kg.
4. Willingness of women of child-bearing potential (WOCBP[2]) to use a culturally acceptable, highly effective method of contraception (see appendix 4), which is effective before the first IP intake and for at least 33 days after the last IP intake.
5. Willingness of male participants, in each course of study treatment: from first day of IP intake to 33 days after the last IP intake, to
    - refrain from donating sperm.
    - use a male condom for vaginal intercourse with female partners of childbearing potential in each course of study treatment. Male participants should also be advised of the benefit for a female partner to use a highly effective method of contraception throughout the same duration, as a condom may break or leak.
6. Capable of giving signed informed consent which includes compliance with the requirements and restrictions listed in the informed consent form (ICF) and in this protocol.
7. Participant is deemed clinically suitable for a placebo-controlled study of the duration planned.

Disease Specific Inclusion Criteria:

[2]The definition of WOCBP will be applied in the study as follows: a woman is considered of childbearing potential, i.e. fertile, following menarche and until becoming post-menopausal unless permanently sterile. Permanent sterilization methods include hysterectomy, bilateral salpingectomy and bilateral oophorectomy. A postmenopausal state is defined as no menses for 12 months without an alternative medical cause (Clinical Trials Facilitation and Coordination Group, 2020)

Disease Specific Inclusion Criteria:

Onchocerca volvulus

- Presence of at least 1 excisable subcutaneous nodule/ onchocercoma detected through palpation
- *O. volvulus* infection diagnosed by skin snip method: documented mf positivity on skin assessment on at least 2 out of 4 skin snips.

Loa loa

- *Loa loa* infection as evidenced by peripheral microfilaremia assessed between 10am-4pm between 100-8,000 mf/ml

Mansonella perstans

- *M. perstans* infection diagnosed by mf-positivity in blood ≥ 200 mf/ml blood (10 mf via finger prick/~50µl)

Trichuris trichiura

- Being able and willing to provide two stool samples at screening and at follow-up assessment.
- Having at least two out of four Kato-Katz slides positive for *T. trichiura* at screening.

4.2. Exclusion Criteria

Participants are excluded from the clinical trial if any of the following criteria apply:

1. Any condition, in the investigator's opinion, that places the participant at undue risk (symptoms, physical signs, vital signs or biological/laboratory signs suggestive of past or present systemic disorders, including but not limited to, cardiovascular, pulmonary, cutaneous, immunodeficiency, hepatic, renal, hematologic, psychiatric disorders, drug / alcohol abuse and other abnormalities likely to interfere with the interpretation of results of the trial) or to significantly affect the study outcomes as judged by the investigator.
2. Current or chronic history of liver disease. This includes (but is not limited to) hepatitis virus infections, drug- or alcohol-related liver disease, nonalcoholic steatohepatitis, autoimmune hepatitis, hemochromatosis, Wilson's disease, α-1 antitrypsin deficiency, primary biliary cholangitis, primary sclerosing cholangitis, or any other liver disease considered clinically significant by the investigator.
3. Pregnant women and breastfeeding women
4. Haemoglobin below 10mg/dl at baseline
5. Aspartate aminotransferase/serum glutamic oxaloacetic transaminase (AST/SGOT) and/or alanine aminotransferase/serum glutamic pyruvic transaminase (ALT/SGPT) and/or total bilirubin > 1.5xULN at baseline.
6. Estimated Glomerular Filtration Rate (using the Modification of Diet in Renal Disease equation, Beck, 2023) < 60 mL/min at baseline.

7. More than one epileptic seizure within the past 5 years
8. *Paragonimus sp*. eggs or *Taenia* sp. proglottids or eggs detectable in stool
9. Acute and/or febrile illness requiring therapy within 14 days prior to baseline
10. Anthelmintic therapy:
    For STH: within previous 1 month prior baseline: ivermectin, moxidectin, diethylcarbamazine, albendazole, mebendazole
    For all other diseases:
    - within previous 6 months prior baseline: ivermectin, moxidectin, diethylcarbamazine, albendazole, mebendazole
    - within previous 12 months: doxycycline (> 2 weeks course) prior baseline
11. Use of concomitant medication that is contraindicated with benzimidazoles and/or known hypersensitivity to benzimidazoles or any other ingredient in the oxfendazole tablet formulation.
12. Current or planned use of another investigational drug at any point during study participation. Participation in any interventional study within 3 months prior to screening or during the study, or within 5 times the half-life of the drug in the previous clinical trial, whichever is longer (time calculated relative to final intake in previous trial)

4.3. Lifestyle Considerations

4.3.1. Meals and Dietary Restrictions

Table 4 Meals and Dietary Restrictions

**Parameter**	**Restrictions/Guidelines**	**Timing**
Food	Treatment days:	Treatment days:<br>Fasting at least 8 hours prior to each dosing and for 2 hours after administration

4.3.2. Alcohol

Table 5 Alcohol

**Parameter**	**Guidelines/Restrictions**	**Timing**
Alcohol	Treatment days:<br>Participants will be instructed to abstain from alcohol and alcoholic products	Treatment days:<br>alcohol and alcoholic products will be forbidden for 24h prior to 1st oxfendazole administration, throughout

		treatment duration until 5 days after last dose.

4.4. Screen Failures

A screen failure occurs when a participant who has consented to participate in the clinical trial is not subsequently assigned to IP.

Minimal information to be collected includes:

- Demography
- Reason for screen failure
- Eligibility criteria
- Adverse Events and Serious Adverse Events (concomitant medications and procedures if received should also be collected)
- Date screen failure

NOTE: Screening failure: Protocol-related events and updates must be recorded in CRFs using the AE (Adverse Event) or Serious Adverse Event (SAE) forms (as appropriate) until the date the participant was determined to be a screening failure. Beyond that date, only serious or medically relevant protocol-related events will be followed-up. It is therefore important to ensure that the date of screening failure is recorded (i.e., medical records, log maintained by site or clinical trial team).

4.4.1. Rescreening Criteria

Participants regarded as screening failures are permitted to re-enter the screening process at a later time-point, under the following circumstances:

- The inclusion and/or non-inclusion criteria preventing the participant's initial attempt to participate have been changed via protocol amendment;
- The participant failed the initial screening because the required wash-out period of prior treatment had not been completed;
- After successful treatment of a medical condition which had been detected during the initial screening and had excluded the participant from enrolment, providing that condition is not a specific exclusion criterion and that the other inclusion / exclusion criteria are respected.

Participants who re-enter the screening process under the circumstances listed above must sign a new informed consent form and will receive a new participant identification.

4.5. Criteria for Temporarily Delaying

Not applicable

5. IP(s) and Concomitant Therapy

IPs, investigational and non-investigational medicinal products intended to be administered to the clinical trial participants during the clinical trial conduct are all pre-specified.

5.1. IP(s) Administered

Oxfendazole, the sulfoxide derivative of fenbendazole, is a white to off-white powder insoluble in water and slightly soluble in methanol, *N,N*-dimethylformamide, and dimethyl sulfoxide.

- Generic name: oxfendazole
- CAS number: 53716-50-0

Table 6. IP(s) Administered

**IP Name**	**Oxfendazole 100 mg tablet**	**Placebo for oxfendazole 100 mg tablet**
**Type**	Drug	Drug
**Dose Formulation**	Immediate-release tablet	tablet
**Unit Dose Strength(s)**	100 mg	Not applicable – placebo (matching 100 mg oxfendazole tablets)
**Dosage Level(s)**	From 4 × 100 mg once daily single dose to 8 × 100 mg once daily on five consecutive days	From 4 × matching placebo tablets once daily single dose to 8 × matching placebo tablets once daily on five consecutive days
**Route of Administration**	Oral	Oral
**Use**	Experimental	Placebo
**IP and NIMP/AxMP.**	IP	IP
**Sourcing**	Manufactured by Syngene International Ltd.	Manufactured by Syngene International Ltd.
**Packaging and Labeling**	Primary package: Aluminum/aluminum foil blister; each blister will contain 4 tablets; Secondary package: Cardboard box (carton); each carton will contain 2 blisters<br><br>Label will contain:<br>- Name (Oxfendazole 100 mg tablets or Placebo for oxfendazole 100 mg tablets)<br>- Participant identification number<br>- Dosing date and time<br>- Name and address of sponsor<br>- Name and contact number of principal investigator<br>- Trial reference code<br>- Batch number<br>- Expiry date<br>- Storage condition<br>- Statements (For oral administration; Four tablets per blister; For clinical trial use only)	Primary package: Aluminum/aluminum foil blister; each blister will contain 4 tablets; Secondary package: Cardboard box (carton); each carton will contain 2 blisters<br><br>Label will contain:<br>- Name (Oxfendazole 100 mg tablets or Placebo for oxfendazole 100 mg tablets)<br>- Participant identification number<br>- Dosing date and time<br>- Name and address of sponsor<br>- Name and contact number of principal investigator<br>- Trial reference code<br>- Batch number<br>- Expiry date<br>- Storage condition<br>- Statements (For oral administration; Four tablets per blister; For clinical trial use only)

Table 7. Clinical Trial Arm(s)

Clinical Trial Arms for Stage 1:

Arm Type	Regimen	Oxfendazole 100 mg tablet	Placebo for oxfendazole 100 mg tablet
**Arm 0:**<br>**Only for *T. trichiura***	Oxfendazole<br>800 mg Multiple Dose (MD) (3 days)	Day 1-3: 8x 100 mg tablet, once daily, oral dose administration, fasted	n/a
**Arm 1:**<br>**Only for *T. trichiura***	Oxfendazole<br>400 mg Single Dose (SD)	Day 1: 4 x 100 mg tablet, once daily, oral dose administration, fasted	Day 1: 4 × placebo tablets, once daily, oral dose administration, fasted<br>Day 2-3: 8 × placebo tablets, once daily, oral dose administration, fasted
**Arm 2:**<br>**Only for *T. trichiura***	Oxfendazole<br>800 mg SD	Day 1: 8 x 100 mg tablet, once daily, oral dose administration, fasted	Day 2-3: 8 × placebo tablets, once daily, oral dose administration, fasted
**Arm 3:**<br>**Only for *T. trichiura***	Placebo<br>SD	n/a	Day 1-3: 8 × placebo tablets, once daily, oral dose administration, fasted
**Arm 4: all diseases except for *T. trichiura***	Oxfendazole<br>400 mg Multiple Dose (MD) (5 days)	Days 1-5: 4 x 100 mg tablet, once daily, oral dose administration, fasted	Days 1-5: 4 × placebo tablets, once daily, oral dose administration, fasted
**Arm 5: all diseases except for *T. trichiura***	Oxfendazole<br>800 mg MD (5 days)	Days 1-5: 8 x 100 mg tablet, once daily, oral dose administration, fasted	n/a
**Arm 6: all diseases except for *T. trichiura***	Placebo<br>MD (5 days)	n/a	Days 1-5: 8 × placebo tablets, once daily, oral dose administration, fasted

Possible Clinical Trial Arm(s) for Stage 2:

Arm Type	Regimen	Oxfendazole 100 mg tablet	Placebo for oxfendazole 100 mg tablet

**Arm 7: all diseases except for *T. trichiura***	Oxfendazole<br>400 mg MD (5 days)	Days 1-5: 4 x 100 mg tablet, once daily, oral dose administration, fasted	Days 1-5: 4 × placebo tablets, once daily, oral dose administration, fasted
**Arm 8: all diseases except for *T. trichiura***	Oxfendazole<br>800 mg MD (5 days)	Days 1-5: 8 x 100 mg tablet, once daily, oral dose administration, fasted	n/a
**Arm 9: all diseases except for *T. trichiura***	Placebo<br>MD (5 days)	n/a	Days 1-5: 8 × placebo tablets, once daily,<br>oral dose administration, fasted
**Arm 10: all diseases except for *T. trichiura***	Oxfendazole<br>800 mg MD (5 days) x 2	Days 1-5: 8 × 100mg tablet, once daily, oral dose administration, fasted, repeated one month later at Days 28-32	n/a
**Arm 11: all diseases except for *T. trichiura***	Placebo<br>MD (5 days) x 2	n/a	Days 1-5: 8 × placebo tablets, once daily, oral dose administration, fasted, repeated one month later at Days 28-32

5.2. Preparation, Handling, Storage, and Accountability

The investigator or designee must confirm appropriate conditions (*e.g.*, temperature) have been maintained during transit for all IP received, and any discrepancies are reported and resolved before use of the IP.

Only participants enrolled in the clinical trial may receive IP, and only authorized site staff may supply, prepare, or administer IP.

All IP must be stored in a secure, environmentally controlled, and monitored (manual or automated) area in accordance with the labeled storage conditions with access limited to the investigator and authorized site staff.

The investigator or authorized site staff is responsible for IP accountability, reconciliation, and record maintenance (*i.e.*, receipt, reconciliation, and final disposition records).

The investigator or designee must maintain appropriate documentation on IP accountability, including the following information:

- IP delivered to the site
- IP inventory at the site
- IP used by each participant
- IP returned to the investigator or designee
- IP destruction

Returned IP (full, partially empty, and completely empty) will be registered in the IP accountability log for each participant at each study site. The site's procedure for destroying IP must be agreed by the sponsor. The site must obtain written authorization from the sponsor before any sponsor-supplied IP is destroyed and IP destruction must be documented on the

appropriate form. Destruction of IP and empty blisters should be done only once drug accountability logs were reviewed and validated by the study monitor.

5.3. Assignment to IP

The trial participants will be randomized to treatment arm using block randomization with varying block sizes using computer-generated random numbers. Participant randomization will be stratified by the country/disease indication with equal allocation to each treatment arm. The randomization setup will be done for stage 1 and stage 2 separately. All participants will be assigned to randomized IP administration using an interactive web response system (IWRS). Before the clinical trial is initiated, the site staff responsible for the randomization will be trained on the IWRS. A detailed description of the randomization process will be provided in the trial randomization plan.

5.4. Blinding, Masking

Study team members, including the pharmacovigilance team, and with the exception of designated unblinded personnel described below, and participants will be blinded to treatment assignment (drug or placebo) throughout the duration of the study. To preserve blinding, blinded treatment kits (drug or placebo) will be allocated to participants via an IWRS system. The randomisation code will be managed by the designated unblinded statistician, and access to treatment allocation information will be strictly limited to the designated unblinded study team members who require access for their activities e.g., unblinded pharmacovigilance staff - for expedited safety reporting only, and staff involved in bioanalytical analysis and evaluation, IP packaging and labelling, and quality control related to these activities.

The blinding will be kept within dose regimen arms. The IWRS will be programmed with blind-breaking instructions. In case of a medical emergency (unblinding of treatment allocation may be necessary for medical care), the investigator has the sole responsibility for determining if unblinding of a participants' IP assignment is warranted. Participant safety must always be the first consideration in making such a determination. If the investigator decides that unblinding is warranted, the investigator may, at the investigator's discretion, contact the sponsor to discuss the situation prior to unblinding a participant's IP assignment unless this could delay emergency treatment for the participant. If a participant's IP administration assignment is unblinded, sponsor must be notified within 24 hours of this occurrence. The date and reason for the unblinding must be recorded.

Sponsor's drug safety and pharmacovigilance staff may unblind the IP assignment for any participant with an SAE if the SAE requires unblinding for expedited regulatory report be sent to one or more regulatory agencies, in accordance with local regulations.

A copy of the report, identifying the participant's intervention assignment, may be sent to investigators in accordance with local regulations and/or sponsor policy.

5.5. IP Administration Compliance

The person responsible for pharmacy at site will prepare the dose for each participant and record the participant ID, dosing date, and dosing time on the label, if applicable. Additional details regarding the labelling and dispensing will be described in the pharmacy manual. The participants must fast overnight for at least 8 hours prior to administration of study drug/placebo. Study medication must be taken with water only. Participants must remain fasted for two hours after the administration of study drug/placebo.

Participants will receive IP directly from the investigator or designee under medical supervision. The date and time of each dose administered in the clinic will be recorded in the source documents. The dose of IP and clinical trial participant identification will be confirmed at the time of dosing by a member of the clinical trial site staff other than the person administering the IP.

Overdose with the IP should be managed in accordance with the information Investigator Brochure (IB) for the IP. In case of suspected overdose, symptomatic and supportive therapy should be given as appropriate.

If vomiting occurs within 15 minutes following study drug intake, doses will be replaced. No redosing will be provided, if vomiting occurs later. The participant will be followed for safety and pharmacokinetic analyses. Vomiting will be documented as an AE. The onset time and frequency will be documented in the source documents. Participants in arms 4-11 who vomit after study drug administration will receive their next dose of study drug according to the protocol.

5.6. Dose Modification

No dose reduction within an arm is planned.

5.7. Continued Access to IP after the End of the Clinical Trial

Participants will be referred to national elimination program activities in each country.

5.8. Treatment of Overdose

All overdoses with the IP will be recorded in the electronic case report form (eCRF). If the overdose is associated with an AE, it will also be reported as an AE. Overdose involving a medication error, misuse or abuse of the IP should be recorded in the eCRF and participant's medical files and shall be reported as deviation.

In the event of an overdose, the investigator should:

- Evaluate the participant to determine, in consultation with the sponsor Medical Responsible, if possible, whether IP should be interrupted or whether the dose should be reduced.
- Closely monitor the participant for any AE/SAE and laboratory abnormalities

5.9. Prior and Concomitant Therapy

Any medication or vaccine (including over-the-counter or prescription medicines, recreational drugs, vitamins, and/or herbal supplements) or other specific categories of interest that the participant is receiving at the time of enrollment or receives during the clinical trial must be recorded along with:

- International non-proprietary name (i.e. generic name)
- Reason for use
- Dates of administration including start and end dates
- Dosage information including dose and frequency

Sponsor should be contacted if there are any questions regarding concomitant or prior therapy.

Since oxfendazole is not considered an inducer of CYP3A4, no influence on the efficacy of oral contraceptives is expected.

5.9.1. Rescue Medication and End of Clinical Trial medication

The clinical trial site will supply the following rescue medication that will be provided by sponsor or obtained locally. The following rescue medications may be used for symptomatic participants:

- *T. trichiura: albendazole 400mg on 3 consecutive days*

  (https://www.cdc.gov/sth/hcp/clinical-care/?CDC_AAref_Val=https://www.cdc.gov/parasites/whipworm/health_professionals/index.html)

- *O. volvulus: ivermectin (150µg/kg body weight) 1 day.*

Additional medications for all 4 diseases may be given based on symptoms and country specific guidance.

Although the use of rescue medications is allowable at any time during the clinical trial, the use of rescue medications should be delayed, if possible, following the administration of IP.

At the end of the clinical trial, the participant may be referred to the national control programme, or offered relevant medications by the investigator in case of remaining infection.

The date and time of rescue medication and end of clinical trial medication administration as well as the name and dosage regimen must be recorded.

5.9.2. Permitted Concomitant Therapy

Any not prohibited medication can be used with caution with respect to participant safety, the investigator can contact the sponsor in case of doubt.

5.9.3. Prohibited Concomitant Therapy

Throughout the entire clinical trial, the use of medication that may have an impact on the clinical trial objectives is prohibited:

- Anthelmintic treatment: ivermectin, moxidectin, diethylcarbamazine, albendazole, mebendazole and doxycycline. Except if required for symptoms according to 6.9.1 (rescue medication)
- Drugs with known hepatoxicity or with myelosuppressive activity.
- Drugs prohibited while on clinical trial up to end of exposure (day 18, and day 45 for Arms 10, 11), CYP3A4 substrates (Bauters, 2019).

Benzodiazepines[a]<br>Budesonide<br>Calcium Channel Blockers[b]<br>Carbamazepine	Corticosteroids<br>Etoposide<br>Immunosuppressives[c]<br>Macrolide antibiotics[d]	Statins[e]<br>Steroids[f]<br>Miscellaneous[g]

[a]Alprazolam, diazepam, midazolam,[b]Amlodipine, diltiazem, verapamil, [c]Cyclosporine, tacrolimus, sirolimus, [d]Clarithromycin, erythromycin, NOT azithromycin, [e]Atorvastatin, NOT pravastatin, simvastatin, [f]Estradiol, progesterone, testosterone, [g]Aprepitant, fentanyl, ondansetron, thiotepa, zolpidem

- Drugs prohibited while on clinical trial up to end of exposure (day 18, and day 45 for Arm 10, 11), CYP1A2 substrates (Gelbe Liste, 2019).

• Alfentanil<br>• Alosetron<br>• Amitriptylin<br>• Aminophyllin<br>• Betaxolol<br>• Cinacalcet<br>• Clopidogrel<br>• Clozapin<br>• Coffein<br>• Clomipramin<br>• Cyclobenzaprin<br>• Doxepin<br>• Duloxetin<br>• Estradiol<br>• Flutamid<br>• Fluvoxamin	• Haloperidol<br>• Imipramin<br>• Lidocain<br>• Melatonin<br>• Methadon<br>• Mexiletin<br>• Mianserin<br>• Mirtazapin<br>• Nabumeton<br>• NAPQI (Paracetamol-Metabolite)<br>• Naproxen<br>• Olanzapin<br>• Ondansetron<br>• Paracetamol<br>• Phenacetin<br>• Pirfenidon	• Propranolol<br>• Rasagilin<br>• Rifabutin<br>• Riluzol<br>• Ropinirol<br>• Ropivacain<br>• Rucaparib<br>• Tacrin<br>• Terbinafin<br>• Theophyllin<br>• Tiotixen<br>• Tizanidin<br>• Triamteren<br>• Trifluoperazin<br>• Verapamil<br>• Warfarin<br>• Zileuton<br>• Zolmitriptan

6. Discontinuation of IP Administration and Participant Discontinuation/Withdrawal

Discontinuation of specific sites or of the clinical trial as a whole are detailed in Appendix 1.

6.1. Discontinuation of IP

At all times, participants' medical teams remain responsible for decisions about participants' care and safety. Hence, if the medical team decides that a discontinuation from the randomly allocated treatment arm is needed then this should be done.
In rare instances, it may be necessary for a participant to permanently discontinue IP. If IP is permanently discontinued, the participant should, if at all possible, remain in the clinical trial to be evaluated for safety. See the SoA for data to be collected at the time of discontinuation of IP and follow-up and for any further evaluations that need to be completed.

The IP will be discontinued in the following situations:

- If the participant requests discontinuation of treatment with IP.
- If the participant requires treatment with a prohibited concomitant medication.
- If the participant develops contraindications to the IP.
- If clinical reasons arise that are considered life-threatening by the investigator, or if the investigator believes that it is in the best interest of the participant.
- Pregnancy (refer to section 7.5.6)

The reason and date for IP discontinuation will be recorded in the source documents and eCRF.

6.1.1. Liver Chemistry Stopping Criteria

Permanent discontinuation of IP for abnormal liver tests is required by the investigator when a participant meets one of the conditions outlined in Appendix 6, Section 9.6.1 Liver Safety Management, or in the presence of abnormal liver chemistries not meeting protocol-specified stopping rules if the investigator believes that it is in best interest of the participant.

6.1.2. Hematologic Stopping Criteria

Permanent discontinuation of IP for abnormal hematology tests is required by the investigator when a participant meets one of the conditions outlined in Appendix 6, Section 9.6.2 Hematologic Safety Management, or in the presence of abnormal hematologic tests not meeting protocol-specified stopping rules if the investigator believes that it is in best interest of the participant.

6.1.3. Temporary Discontinuation

If treatment with the IP is interrupted for any reason, the decision to resume treatment will be taken by the investigator. The aim is to complete the treatment course, assuming it is safe to do so.

6.2. Participant Discontinuation/Withdrawal from the Clinical Trial

A participant may withdraw from the clinical trial at any time at the participant's own request for any reason (or without providing any reason).
A participant may be withdrawn at any time at the discretion of the investigator for safety, behavioral, or compliance reasons.
At the time of discontinuation from the clinical trial, if possible, an early discontinuation visit should be conducted, as shown in the SoA. See SoA for data to be collected at the time of clinical trial discontinuation and follow-up and for any further evaluations that need to be completed.
The participant will be permanently discontinued from the IP administration and the clinical trial at that time.
If the participant withdraws consent for disclosure of future information, sponsor may retain and continue to use any data collected before such a withdrawal of consent.
If a participant withdraws from the clinical trial, the participant may request destruction of any samples taken and not tested, and the investigator must document this in the site clinical trial records.

6.3. Lost to Follow up

A participant will be considered lost to follow-up if the participant repeatedly fails to return for scheduled visits and is unable to be contacted by the clinical trial site.

The following actions must be taken if a participant fails to return to the clinic for a required clinical trial visit:

- The site must attempt to contact the participant and reschedule the missed visit as soon as possible, counsel the participant on the importance of maintaining the assigned visit schedule and ascertain whether the participant wishes to and/or should continue in the clinical trial.
- Before a participant is deemed lost to follow-up, the investigator or designee must make every effort to regain contact with the participant (where possible, 3 telephone calls on different days, and if necessary, a certified letter to the participant's last known mailing address or local equivalent methods). These contact attempts should be documented in the participant's medical record.
- Should the participant continue to be unreachable, the participant will be considered to have withdrawn from the clinical trial and will be recorded as loss to follow-up in the eCRF.

7. Clinical Trial Assessments and Procedures

Clinical trial procedures and their timing are summarized in the SoA. Protocol waivers or exemptions are not allowed.

Adherence to the clinical trial design requirements, including those specified in the SoA, is essential and required for clinical trial conduct.

All screening evaluations must be completed and reviewed to confirm that potential participants meet all eligibility criteria. The investigator will maintain a screening log to record details of all participants screened and to confirm eligibility or record reasons for screening failure, as applicable.

In the event of a significant clinical trial continuity issue (e.g., caused by a pandemic), alternate strategies for participant visits, assessments, medication distribution and monitoring may be implemented by sponsor or the investigator, as per local health authority/ethics requirements.

7.1. Assessments and Procedures

The evaluation and procedure for the study include screening, baseline assessment, and follow-up at regular intervals. The screening occurs between month -3 and day -1, while the baseline assessment occurs on day -2 to day -1.

Different assessments for a visit may be conducted across more than one day, providing all are conducted within the window specified for that visit in the SoA. The following procedures and data collection will be performed.

7.1.1. Screening

Month -3 to Day -1

All potential participants will have a screening visit prior to inclusion of the study. Potential participants will be informed about the study arms, design, inclusion and exclusion criteria and procedures. Informed consent will be obtained. Once the informed consent is signed, the following screening procedures will be undertaken:

- Assessment of eligibility (Inclusion and Exclusion Criteria)
- Collection of medical history
- Pregnancy test in WOCBP (highly sensitive urine or serum test), verification of last menstrual period, and confirmation of contraceptive method used (see appendix 4)
- Collection of concomitant treatments
- Collection of Adverse Events
- Blood samples for microscopic diagnostics of microfilariae
- Stool samples for parasitological assessment
- Skin snips for parasitological assessment
- Nodule palpation

7.1.2. Baseline

Day – 2 to -1

- Assessment of eligibility (Inclusion and Exclusion Criteria)
- Collection of medical history
- Demographic data
- Vital signs (pulse, blood pressure, temperature, weight, and height)
- Complete physical examination
- Neurological examination
- Blood sample collection for safety laboratory test
- Urine sample collection for safety laboratory test
- Pregnancy test in WOCBP (highly sensitive urine or serum test - within 24 hours of 1st dose of IP), verification of last menstrual period, and evaluation of contraceptive method and potential for failure
- Collection of concomitant treatments
- Collection of Adverse Events
- Collection of signs and symptoms (disease-related, for *Onchocerca volvulus, Loa loa* and *Mansonella perstans* only)
- Blood samples for microscopic diagnostics of microfilariae to be done if baseline visit is conducted more than 2 months apart from screening visit
- Skin snips for parasitological assessment to be done if baseline visit is conducted more than 2 months apart from screening visit

7.1.3. Randomisation and Treatment Period

Day – 1 to 1

- Assessment of eligibility (Inclusion and Exclusion Criteria)
- Randomisation
- Collection of concomitant treatments
- Collection of Adverse Events

D1 Predose

- Vital signs (pulse, blood pressure, temperature)
- Physical examination (symptom directed)
- Collection of concomitant treatments
- Collection of Adverse Events

D1 to D5 (only Day -3 for *T. trichiura* Arms 0 to 3)

- Treatment administration
- Vital signs (pulse, blood pressure, temperature)
- For WOCBP - evaluation of contraceptive method and potential for failure
- PK sampling (refer to Section 7.6) (Stage 1 only, except for *T. trichiura*)
- Collection of concomitant treatments
- Collection of Adverse Events
- Blood sample collection for safety laboratory test D3 (post-dose, *T. trichiura* only), D5 (post-dose, except for *T. trichiura*)

- Urine sample collection for safety laboratory test D3 (post-dose, *T. trichiura* only), D5 (post-dose, except for *T. trichiura*)

D26-27, Arm 10, 11 only

- Vital signs (pulse, blood pressure, temperature)
- Physical examination (complete)
- Neurological examination
- Pregnancy test in WOCBP (highly sensitive urine or serum test - within 24 hours of 1st dose of IP in this course of treatment), verification of last menstrual period, and evaluation of contraceptive method and potential for failure.
- Blood sample collection for safety laboratory test
- Urine sample collection for safety laboratory test
- Collection of concomitant treatments
- Collection of Adverse Events

D28 Predose, Arm 10, 11 only

- Vital signs (pulse, blood pressure, temperature)
- Physical examination (symptom directed)
- Collection of concomitant treatments
- Collection of Adverse Events

D28 to D32, Arm 10, 11 only

- Treatment administration
- Vital signs (pulse, blood pressure, temperature)
- For WOCBP - evaluation of contraceptive method and potential for failure
- Collection of concomitant treatments
- Collection of Adverse Events
- Blood sample collection for safety laboratory test D32 (post-dose)
- Urine sample collection for safety laboratory test D32 (post-dose)

7.1.4. Follow-Up Period

D18 Arms 0-9, (final follow-up visit for *T. trichiura*, except WOCBP); Day 45 for Arms 10, 11 only; or early discontinuation

- Vital signs (pulse, blood pressure, temperature)
- Complete physical examination
- Neurological examination
- Blood sample collection for safety laboratory test
- Urine sample collection for safety laboratory test
- Pregnancy test in WOCBP (highly sensitive urine or serum test), verification of last menstrual period, and evaluation of contraceptive method and potential for failure.

- Collection of concomitant treatments
- Collection of Adverse Events
- Collection of signs and symptoms (disease-related, for *Onchocerca volvulus, Loa loa* and *Mansonella perstans* only)
- Stool samples for parasitological assessment (not for Arms 10, 11)
- Blood samples for microscopic diagnostics of microfilariae
- Skin snips for parasitological assessment
- Nodule palpation

47 days post last dose (WOCBP, final follow-up visit for *T. trichiura* WOCBP)

- Pregnancy test in WOCBP (highly sensitive urine or serum test) and verification of last menstrual period
- Collection of Adverse Events

Month 3, Month 6, Month 9 and Month 12 only for *O. volvulus* infection, *L. loa* infection and *M. perstans* infection

- Vital signs (pulse, blood pressure, temperature) at months 6 and 12 only
- Physical examination (symptom directed) at months 3 and 9 only
- Complete physical examination at months 6 and 12 only
- Neurological examination at months 6 and 12 only
- Collection of concomitant treatments
- Collection of Adverse Events
- Collection of disease-related signs and symptoms (for *Onchocerca volvulus, Loa loa* and *Mansonella perstans* only)
- Blood samples for microscopic diagnostics of microfilariae
- Skin snips for parasitological assessment
- Nodule palpation
- Nodulectomy at month 12 only for onchocerciasis

7.1.5. Unscheduled visits

Unscheduled visits may occur at any time during the study. Unscheduled visit forms will be used to document data collected.

- Symptom directed physical exam
- Vital signs
- AE-monitoring
- Review concomitant treatments
- Clinical safety laboratory evaluations

7.2. Baseline Procedures

7.2.1. Demographic Data

The following demographic data items will be recorded in the eCRF:

- Year of birth and/or age
- Sex

7.2.2. Medical History

The following medical history items will be recorded:

- Relevant past medical history, including prior diseases with start and end dates, prior treatment with start and end dates
- Surgical history, including indications and dates
- Current medical history, including start date and severity of ongoing conditions

7.2.3. Concomitant Medication

The following items for concomitant medication will be recorded:

- International non-proprietary name (i.e. generic name); dose, frequency, route of administration and indication
- Start and stop dates

7.2.4. Incidental Findings

During a clinical trial, previously undiagnosed medical conditions unrelated to the aims of the trial are sometimes discovered as a result of trial examinations or investigations. These are referred to as “incidental findings”. In case there would be such a medical finding, the trial site investigator is responsible for ensuring the participant is referred to receive the appropriate medical care or advice.

An Incidental Findings Plan will describe how findings will be identified, managed, and disclosed to participants. Informed Consent will explain to the participant that incidental findings may occur and how the findings may be communicated to him/her, and/or communicated to another healthcare professional for further investigation and treatment.

Unexpected events (such as social or criminal issues, unrelated to trial procedures or findings), in the case they are recognized by the trial site investigators, may warrant referral of the participant to an appropriate service or practitioner and/or, if required by local law, reporting of the event to the appropriate authorities following a discussion with the trial participant. The investigator remains responsible to assess if it does not inappropriately breach medical confidentiality, and whether the participant requires or not withdrawal from the clinical trial.

7.3. Efficacy Assessments

7.3.1. *Onchocerca volvulus*

- **Skin Snips / Nodule Palpation**

Onchocerca volvulus: Nodule palpation will be conducted, with documentation of *Onchocerca volvulus* nodules. A 1 to 2 mg biopsy of the skin will be taken and will be put in physiological solution. Typically, four skin snips are taken from the iliac crest and upper calf. Microfilariae can be counted via microscopy. Results are quantifiable and are usually expressed as mf/mg skin. For quality control, skin snips will be re-read by a second reader. Skin snips will be preserved for later PCR analysis for remaining mf in the skin.

- **Nodulectomy and Histological Assessment of Nodules**

Onchocerca volvulus: Nodulectomy (M12) will be performed under local anesthesia using aseptic standard surgical procedures. The participant will be scheduled for an operation date and will be transported from their community either the evening before or early in the morning to the investigational centre. Following surgery, the participant will usually stay overnight or longer if medically indicated. The participants will then return to their communities where a nurse will continue wound management for approximately 10 days.

Wound Management will begin following incision and continue until the wounds are completely healed. Participants will be advised to stay off work for the first week post-surgery to promote wound healing. Sutures will be removed approximately 8 days post-surgery. Evidence of an effect on adult worms will be obtained from histological examinations and will be performed according to published procedures (Hoerauf et al., 2008).

7.3.2. *Loa loa*

Thick smear microscopy will be performed using the Lambaréné method, using a fixed volume (50µL of blood on a fixed slide) and read at 10x magnification with a known microscopic optical field diameter in order to allow quantification of microfilaraemia per optical field.

7.3.3. *Mansonella perstans*

Thick blood film technique. A fixed volume of peripheral whole blood (50 µl) will be transferred to a fixed slide, stained with Giemsa and examined for microfilariae using a microscope at 10x magnification.

7.3.4. *Trichuris trichiura*

Trichuris trichiura: At screening, all participants will be asked to provide two stool samples. From each stool specimen, duplicate Kato-Katz thick smears (41.7 mg each) will be prepared and read under a microscope for eggs of *T. trichiura, Ascaris lumbricoides* and hookworm by experienced technicians. Eggs per gram (EPG) will be assessed by adding up the egg counts from the quadruplicate Kato-Katz thick smears and multiplying this number by a factor of six. For quality control of *T. trichiura* and *A. lumbricoides* egg counts, 10% of slides will be re-read by another laboratory technician the same day. Results are considered correct if the following tolerance margin is not exceeded: (i) No difference in presence/absence of *T. trichiura* and *A. lumbricoides*

(ii) egg counts are +/-10 eggs for counts ≤100 eggs or +/-20% for counts >100 eggs (for each species separately). In case discrepancies above the tolerance margin are noted in one or more slides, the respective slides are re-read by another laboratory technician. The new results are discussed, so that in case of discordant results, slides can be re-evaluated to reach consensus. All microscopically analysed quadruplicate Kato-Katz thick smears will be destroyed after passing the quality control. The same diagnostic approach will be applied at 18 days after treatment. Additional stool parasites will be recorded.

7.4. Safety Assessments

7.4.1. Physical Examinations

A full physical examination including a complete review of body systems will be performed at the time-points specified in the Schedule of Events. At some time-points, a shortened symptom-directed physical examination will be performed. Detailed instructions for the physical examination will be provided in the Study Operations Manual/SOPs.

7.4.2. Vital Signs

The following parameters will be assessed for vital signs with one measurement at each specified time-point.

- Supine systolic BP
- Supine diastolic BP
- Supine heart rate
- Resting respiratory rate
- Temperature
- Weight
- Height

Measurements will be repeated if values are outside of the ranges defined in the Study Operational Manual/SOPs. If the result of the repeat measurement is still out of range, it will be assessed for clinical significance and may be reported as an AE.

7.4.3. Neurological Assessment

A brief neurological examination will be performed at various time-points specified in the Schedules of Events. Detailed instructions for the short neurological examination will be provided in the Study Operations Manual/SOPs.

7.4.4. Clinical Safety Laboratory Tests

Blood samples will be obtained only after measurements of body temperature, vital signs and physical examination. Blood samples will be collected by venipuncture during screening, during follow-up visits and in case of an unforeseen visit if deemed necessary by the investigator. In case of needle stick injury, the local personnel in charge will be contacted immediately and the internal institutional guidelines should be followed.

See Appendix 2 for the list of clinical laboratory tests to be performed and the SoA for the timing and frequency.
The investigator must review the laboratory results, to evaluate if the test result is normal or abnormal. This should be made considering normal values in the studied population/country and previous results. If abnormal (after retest and after having checked the absence of technical errors), the investigator will assess if this finding is “clinically significant” or not and will document this review in source documents.
Any “clinically significant” abnormalities occurring during the clinical trial and after medicinal product administration should be recorded as an AE. The laboratory results must be retained with source documents.

Abnormal laboratory findings/evolution associated with the underlying disease are not considered clinically significant (therefore not to be reported as an AE) unless judged by the investigator to be more severe (e.g. abnormal worsening) than expected for the participant’s condition.

All abnormal laboratory tests with values considered clinically significant during participation in the clinical trial should be repeated until the values return to normal or baseline or are no longer considered clinically significant (no more an AE) by the investigator or delegate.

If clinically significant values do not return to normal/baseline within a period of time judged reasonable by the investigator, the etiology should be identified, and sponsor notified.

All protocol-required laboratory tests, as defined below, must be conducted in accordance with the laboratory manual and the SoA.

If abnormal laboratory values from non-protocol-specified laboratory tests performed at the institution’s local laboratory are considered clinically significant by the investigator (e.g., require a change in participant management or dose modification), then the results must be recorded as AE (and SAE, if applicable).

7.4.5. Pregnancy Testing

A highly sensitive pregnancy test, i.e. beta human chorionic gonadotropin level, and the verification of the date of the last menstrual period will be performed in WOCBP at the time-points specified in the Schedule of Events.

If the menstrual period is delayed, with over one month between menstruations, confirmation of absence of pregnancy with a pregnancy test is mandatory. This recommendation also applies to WOCBP with infrequent or irregular menstrual cycles.

7.4.6. Urine Test

Urine samples will be collected at various time-points specified in the SoA for urine dipstick analysis. Urine dipstick analysis will provide information about proteinuria or glucosuria for the evaluation of kidney function.

These urine samples will also be stored. In case kidney impairment is suspected because of proteinuria, haematuria, glucosuria or elevated serum-creatinine in the respective participant, further microscopic analysis of the urine (from screening and day 3) will be performed retrospectively to investigate for potential microfilaremia as a cause of kidney impairment.

See Appendix 2 for the list of clinical laboratory tests to be performed and the SoA for the timing and frequency.

7.5. Adverse Events, Serious Adverse Events, and Other Safety Data Collection, Assessment and Reporting

The definitions of adverse events (AEs), serious adverse events (SAEs) and associated method for AEs assessments (severity, seriousness and causality) of AEs (with each IP, if applicable) can be found in Appendix 3.

The investigator and any qualified designees are responsible for detecting, documenting, and recording events that meet the definition of an AE or SAE and remain responsible for following up. This includes events reported by the participant (or, when appropriate, by a caregiver, surrogate, or the participant's legally authorized representative).

The process for notification to sponsor (including follow-up information and completing "SAE forms") are provided in the next sections.

7.5.1. Time Period and Frequency for Collecting AE and SAE Information

All SAEs will be collected from the signing of the informed consent form (ICF) (during the screening period only if assessed as related to clinical trial participation) until the final follow-up visit at the timepoints specified in the SoA.

All AEs will be collected from the signing of the informed consent form (ICF) until the final follow-up visit at the timepoints specified in the SoA.

<u>Screening Period:</u>

Medical occurrences/conditions that begin before the start of trial treatment but after obtaining informed consent will be recorded as medical history/current medical conditions (not as AEs), except if the events could be caused to specific circumstances in which the participant has been placed (e.g. withdrawal of a previous drug to enter in the clinical trial or "protocol related").

Such event is not considered as AE *per se*, but for monitoring purpose, any non-serious "event" to be reported after signature of the ICF should be reported on an "AE form".

<u>After Trial Closure:</u>

Investigators are not obligated to actively seek information on AEs or SAEs after conclusion of the study participation. However, if the investigator learns of any SAE, including a death, at any time after a participant has been discharged from the study, and the investigator considers the event to be reasonably related to the study intervention or study participation, the investigator must promptly notify the sponsor.

7.5.2. Method of Detecting AEs and SAEs

Safety will be assessed through routine follow-up of participants by clinical trial personnel at the time-points indicated in the SoA to identify and collect adverse events (AEs).

In addition, to avoid bias in eliciting AEs, each participant will be questioned about the occurrence of adverse events, with general, non-leading questions such as : "Since xxx (e.g. last visit) have you had any health problem?" or "How are you feeling?".

AEs may also be directly observed by trial personnel or spontaneously reported by participants and should be reported by the investigator using concise medical terminology.

Information on AEs (including their assessments) must be evaluated by a physician.

Refer to detailed definitions (including assessments) in Appendix 3.

7.5.3. Reporting of SAE

In case the AE qualifies for an SAE, the investigator should notify the SAE whether or not the event is considered trial medication-related, immediately **and no later than 24h of awareness of the SAE** to sponsor.

If the forms (SAE forms) cannot be completed within 24h of awareness, it can be replaced by

- an e-mail notification containing key information for a valid case and to be sent within these timelines,
- promptly followed by more information with or without the SAE form. In the latter case the form will be provided as a future follow-up and not processed as part of the initial notification.

If the investigator is not able to contact sponsor by e-mail or has problems with e-mail/internet connection, alternatively, events could be first preliminary notified (with key information) by telephone, / or using Short Message Service (SMS) or other tools (received information to be reported in an e-mail or phone report by the recipient).

7.5.4. Follow-up of AEs and SAEs

After the initial AE/SAE report, the investigator is required to proactively follow each participant at subsequent visits/contacts.

All SAEs will be followed until final conclusion of the case (outcome is assessed as recovered, recovered with sequelae or death, or until the investigator assesses event as "chronic" or "stable"), the event is otherwise explained, or the participant is lost to follow-up (as defined in Section 6.3)

A closing report will be sent by the investigator after the final conclusion on the case (outcome is assessed as recovered, recovered with sequelae or death, or until the investigator assesses event as "chronic" or "stable").

When new significant information becomes available to the investigator, follow-up forms/notification should be sent by the investigator to sponsor:

- Promptly (preferably within 24 hours of awareness), if they contain key information such as assessment of seriousness or causality, fatal outcome,

- In a timely manner for all other follow-up information.

The investigator is obligated to perform or arrange for the conduct of supplemental measurements and/or evaluations as medically indicated or as requested by sponsor to elucidate the nature and/or causality of the AE or SAE as fully as possible. This may include additional laboratory tests or investigations, histopathological examinations, or consultation with other health care professionals.

7.5.5. Regulatory Reporting Requirements for SAEs

- Prompt notification by the investigator to sponsor of an SAE is essential so that legal obligations and ethical responsibilities towards the safety of participants and the safety of an IP under clinical investigation are met.
- The sponsor has a legal responsibility to notify both the local regulatory authority and other regulatory agencies about the safety of an IP under clinical investigation. Sponsor will comply with country-specific regulatory requirements relating to safety reporting to the regulatory authority, institutional review boards (IRBs)/independent ethics committees (IECs), and investigators.
- An investigator who receives an investigator safety report describing an SAE or other specific safety information (e.g., summary or listing of SAEs) from sponsor will review and then file it along with the IB and will notify the IRB/IEC, if appropriate according to local requirements.

7.5.6. Exposure During Pregnancy

- Details of all exposures during pregnancies in female participants will be collected after the start of IP and until 47 days post last dose.
- In case of exposure during pregnancy**,** the notification of exposure during pregnancy should be made no later than **24h of awareness** by the investigator to sponsor (the exact process is defined in the trial-specific Safety Management Plan (SMP)).
- While pregnancy itself is not considered to be an AE or SAE, any pregnancy complication or termination of a pregnancy for medical reasons will be reported as an AE or SAE.
- An unfavorable pregnancy outcome (e.g., any type of abortion (including spontaneous), fetal death, stillbirth, congenital anomality) are considered SAEs and will be reported as such (an SAE form will be necessary in addition to the Pregnancy Surveillance Form (PSF)).
- In the case of a live birth, a medically qualified person (preferably a paediatrician) should assess the new-born at the time of birth, notify sponsor and submit a report based on the paediatrician examination using an appropriate reporting form (i.e. a Child Surveillance Form, CSF) as soon as new information becomes available to the investigator (following a similar communication process and timelines for SAEs). Follow-up of children exposed to drug in utero is proposed to the pregnant trial participant by the investigator, until the children reach 2 years of age. It shall be reported using the CSF form and the information will be forwarded to sponsor.

- If the forms “Pregnancy Surveillance Form”/” Child Surveillance Form” (PSF/CSF) cannot be completed within 24h of awareness, it can be replaced.
  - by an e-mail notification containing key information and to be sent within 24 hours of awareness by the investigator,
  - followed by significant new information with or without the PSF/CSF form (as per each CT protocol). In this later case the form will provided as a future follow-up and not processed as part of the initial notification.
- Any post-trial pregnancy-related SAE considered reasonably related to the IP by the investigator will be reported to sponsor as described in Section 7.5.3. While the investigator is not obligated to actively seek this information in former trial participants, he or she may learn of an SAE through spontaneous reporting.
- Any female participant who becomes pregnant while participating in the trial will discontinue IP.
- In case of a male trial participant´s female sexual partner conceiving during the protocol-mandated period for use of condoms, the notification of pregnancy should be made no later than **24h of awareness** by the investigator to sponsor (the exact process is defined in the SMP). A specific written informed consent will be sought from the trial participant´s sexual female partner and, if granted, any pregnancy will be followed, and the status of mother and/or child will be reported to the sponsor. The PSF/CSF will be completed as described above.

7.6. Pharmacokinetics

In stage 1, blood samples for PK analyses of oxfendazole will be collected at the time-points (refer below). Sample collection details, including the per participant sampling schedule in case of sparse PK, will be provided in the PK manual.

Table 8. Time-points and Time Windows for PK Sample Collection in stage 1

Study Period	Treatment					
Visit	Day 5					
Time point	1h pre-dose	1h	2h	3h	6h	8h
Time window	+/- 30 min	+/- 30 min	+/- 30 min	+/- 30 min	+/- 30 min	+/- 30 min

Oxfendazole concentrations will be measured using validated analytical methods. Detailed instructions will be included in the Laboratory Manual.

Since the PK calculations will be based on actual sampling and dosing times, the data must be thoroughly documented in the participant’s medical file and eCRF.

IP concentration information that would unblind the trial will not be reported to investigative sites or blinded personnel until the trial has been unblinded.

7.7. Pharmacodynamics

Not Applicable

7.8. Genetics

Not Applicable

7.9. Biomarkers

Not Applicable

7.10. Immunogenicity Assessments

Not Applicable

7.11. Medical Resource Utilization and Health Economics

Not Applicable

8. Statistical Considerations

8.1. Statistical Hypotheses

The primary objective is to demonstrate that oxfendazole is superior to placebo in achieving a reduction of primary endpoint (mf or eggs counts) between baseline and follow-up for (18 days for trichuriasis, 12 months for loasis, mansonellosis, and onchocerciasis) each of the four diseases, participants infected with onchocerciasis, loiasis, mansonellosis and trichuriasis. Thus, the null hypothesis to be tested in relation to the primary estimand is as follows:

- Null hypothesis: Active treatment is not different from placebo with respect to the achievement of mf count at 12 months (onchocerciasis, loiasis, mansonellosis).
- Null hypothesis: Active treatment is not different from placebo with respect to the achievement of egg counts at 18 days (trichuriasis).

8.1.1. Multiplicity Adjustment

Within each disease the family-wise error rate across the comparisons of the different doses against control for the primary endpoint will be controlled at one-sided level of 0.025 using the partial conditional error approach for loasis, mansonellosis, and onchocerciasis (outlined in the Statistical Report). For trichuriasis a Bonferroni-Holm adjustment will be applied. No adjustment for multiplicity will be performed across the diseases as hypothesis testing will be done independently.

For each disease, there will be no adjustment for multiplicity between the primary and secondary endpoints, nor between the different secondary endpoints as these analyses are considered to be describing the characteristics of the primary endpoint.

8.2. Analysis Sets

The following populations will be used in the statistical analyses:

Intent-to-treat (ITT):	All randomized participants, and participants will be analyzed according to the randomized treatment assignment
Modified Intent-to-treat (mITT):	All participants who received at least one intake of IP and for which the primary endpoint (M12/D18) was observed OR who died OR took rescue medication
Per protocol (PP):	All participants in the ITT population who were free from major protocol violations that could lead to bias of results
Safety:	All participants who received at least one intake of IP, and participants will be analyzed according to the actual treatment received.

Other analysis populations may be described in the Statistical Analysis Plan.

8.3. Statistical Analyses

8.3.1. General Considerations

A general description covering the main aspects of the statistical analysis of the basket trial is given below. The detailed statistical analysis plan (SAP) that encompasses the entire basket trial will be provided before the first unblinding of efficacy data (as required by ICH E9). When analyses for specific diseases deviate from the overall SAP, a separate SAP will be developed.

8.3.2. Primary Endpoint/Estimand Analysis

- **Main Analytical Approach**

The master SAP for the basket trial will include a more technical and detailed description of the statistical analyses that will apply to all diseases. Any disease-specific analyses will be described in the disease-specific SAPs that will be provided before the unblinding of efficacy data of the respective disease.
The statistical analysis will be performed in alignment with the ICH-E9 standards. All available data will be used in the analysis. The following paragraphs describe the statistical analysis for the evaluation of the primary and secondary endpoints and other analysis that will be performed at the end of the study or in interim analyses (onchocerciasis, mansonellosis and loasis).
Participants demographics and baseline characteristics will be summarized on the ITT set, overall for each disease and by randomized dose groups, by means of summary descriptive statistics.
For qualitative variables (e.g. sex), absolute and relative frequencies will be calculated per treatment group. Data will be visualized by bar plots. For quantitative data (e.g. age), the number of valid observations, mean, standard deviation, standard error, median, geometric mean, interquartile range, minimum and maximum will be calculated for each randomized group. Data will be visualized by boxplots.

The primary analysis will be performed in each of the 4 diseases independently, controlling within each disease the family-wise error rate at the one-sided level of 0.025 adjusting for the multiple comparisons to control. For the *O. volvulus* and *M. perstans* and *L. loa*, adaptive tests will be performed to account for the adaptive dose selection and sample size reassessment. For *T. trichiura* a fixed sample tests will be applied. For all diseases non-parametric tests will be used in the treatment control comparisons, as based on published results from clinical trials investigating similar indications, it is expected that both proposed endpoints follow a mixture distribution with an atom at 0 and a component resembling a lognormal distribution (Gardon et al. 2022; Pion et al. 2013; Wanji et al. 2016). The atom at 0 corresponds to total responders, for which no mf and/or eggs can be detected at the 12 month / 18 day follow-up.

Primary Efficacy for *O. volvulus* and *M. perstans* and *L. loa*:

The primary endpoint of the study is the parasite load at month 12 (mf count). The primary estimand is defined in Section 2.2.

Primary Efficacy Analysis (primary estimand) for *O. volvulus* and *M. perstans* and *L. loa*:

The primary analysis will be based on the mITT population. Below the analysis for a specific disease is described as for each of the three diseases *O. volvulus* and *M. perstans* and *L. loa* an independent and identical analysis strategy will be performed. Within each of the 3 diseases, Cohorts with the same total dose will be combined using the adaptive test.

For each dose the null hypothesis of no treatment effect is tested against the one-sided alternative hypothesis of a positive treatment effect based on non-parametric, independent sample, one-sided Wilcoxon tests (which are equivalent to the Mann-Whitney-U test) and the partial conditional error rate principle to account for adaptions (Klinglmueller et al., 2014). For each dose i= Low dose (L), Medium dose (M), High dose (H) we consider the summary measure probabilistic index or concordance defined as $\Delta(i)=P(Y(0)>Y(i))+0.5\ P(Y(0)=Y(i))$, where $Y(0)$, $Y(i)$ denote the mf counts in the control and dose group i. To participants who died or received rescue medication the highest possible rank will be assigned. Δ corresponds to the parameter of the Mann-Whitney-U test for the comparison of independent groups. If there are no ties, it corresponds to the probability that a randomly selected patient under treatment has a better outcome (lower mf load or lower egg count) than a randomly selected patient from the control group. In addition, both in the cases with and without ties, the probabilistic index is directly related to the "net benefit", defined as $P(Y(0)>Y(i)) - P(Y(0)<Y(i)) = 2\ \Delta(i)-1$ (see, e.g., (Verbeeck et al., 2021)). The net benefit is interpreted as the difference between the probability of superiority and the probability of inferiority.

For each dose i=L, M, H we test the null hypothesis

H0: $\Delta(i)=1/2$ against H1: $\Delta(i)>1/2$

controlling the FWER at one-sided level 0.025 in the strong sense for the comparisons of (up to) 3 doses to control in each disease. No adjustment for the comparison across diseases is planned.

To adjust for multiple testing (3 treatment control comparisons) and account for the adaptive dose selection and sample size reassessment, hypothesis tests using the partial conditional error approach (Klinglmueller et al., 2014) will be applied. The pre-planned tests for each individual null hypothesis, required to apply the partial conditional error rate approach, are combination tests based on the inverse normal combination function. As stage-wise tests, one-sided, independent samples Wilcoxon tests will be applied. Initially, the significance level alpha will be equally split across the three hypotheses. In the interim analysis, after 40 participants per group have been recruited, doses for the second stage are selected and/or the high dose may be started (see below for the selection strategy). According to the partial conditional error rate principle and based on the number and type of treatment arms selected for the second stage, the cumulative conditional error rate across hypothesis will be redistributed to the selected hypothesis to perform the second stage test for each of the selected doses. The second stage tests will also be one-sided, independent sample Wilcoxon tests.

As treatment effect estimates, both the estimated probabilistic index and the net benefit will be reported together with unadjusted 95% confidence intervals. These estimates are not conditioned on baseline covariates.

The SAP will provide further details on the primary analysis.

Interim Analysis and Dose Selection (for *O. volvulus* and *M. perstans* and *L. loa)*:

At the interim analysis, no early stopping for superiority of a dose compared to control is foreseen. The selection decision in the interim analysis will be performed separately for the three diseases and will be mainly based on the mf values at 6 months which are expected to be available for all participants. For the sample size calculation, a specific selection rule based on the mf values at 6 months is assumed which should serve as guidance for the dose selection at the interim analysis (see below). However, the actual decision may additionally take the mf value at 12 month (for participants for which this data is available at the time of the interim analysis), safety data and data from other endpoints into account and may incorporate safety considerations, and data available from the other diseases. In addition, the sample size of each arm may be adapted at the interim analysis based on the observed data. The adaptive testing strategy guarantees family-wise error rate control, regardless of the chosen adaptation strategy.

Dose selection rule assumed in the sample size calculations: One-sided independent sample Wilcoxon tests for the comparison of each active dose against Placebo of the mf value at 6 months are performed based on the first stage data. A dose is stopped for futility in the interim analysis if the first stage p-value falls above the futility boundary alpha1 0.3. There are four different scenarios:

- Scenario 1: Both the comparison of the low and the medium dose results in a p-value smaller than alpha1. In this case both the low and the medium dose are continued in the second stage and the high dose (2 weeks 5 x 800) is not started.
- Scenario 2: The p-value of the medium dose is below alpha1, the p-value of the low dose is larger alpha1. Then the medium dose is continued the second stage and additionally the high dose is started in the second stage. The low dose is dropped.
- Scenario 3: The p-value of the medium dose is larger than alpha1, the p-value of the low dose is below alpha1. In this case both the low and the medium dose are continued in the second stage and the high dose (2 weeks 5 x 800) is not started.
- Scenario 4: Both the p-value of the lower and the medium dose are larger than alpha1. In this case, both doses are dropped and the second stage is performed with the high dose only.

The selection will be performed individually for the 3 diseases. For stage 2, participants are equally allocated to the selected doses and the Placebo group.

In case that the 1x5x800 treatment arm is continued and in addition the repeated 2x5x800 treatment arm is started in stage 2, participants will be randomized to two different placebo groups (5xplacebo or repeated 2x5xplacebo). For the treatment control comparisons, each arm will be compared to the pooled placebo group (including the 5xplacebo and repeated 2x5xplacebo group). While this pooling could introduce a bias if there is a different dropout rate between placebo groups and drop out is correlated with outcome, we expect that this effect will be not relevant. To assess potential biases, dropout rates will be reported by placebo group.

Primary Efficacy Analysis for *T. trichiura*:

For the three tested doses i=L, M, H the null and alternative hypotheses

H0: $\Delta(i)=1/2$ against H1: $\Delta(i)>½$

will be tested, corresponding to the probabilistic index (concordance) with respect to the primary endpoint 18 days egg count. One-sided, independent samples Wilcoxon tests comparing Placebo against each active dose will be calculated. Adjustment for multiplicity will be performed using the Bonferroni-Holm test adjusting for 3 comparisons.

As treatment effect estimates, both the probabilistic index and the net benefit will be reported together with unadjusted 95% confidence intervals.

Handling of Missing Values for all Diseases:

In case of dropouts with the exception of patients who died or received rescue medication, and patients with missing values for other reasons, the primary analyses will be based on the mITT population with all available cases. As a secondary analyses an analysis on the ITT population will be performed using different strategies to impute missing values. Details will be specified in the SAP.

8.3.3. Secondary Endpoint(s)/Estimand(s) Analysis

For *O. volvulus* and *M. perstans* and *L. loa*, the secondary endpoint of the study is mf reduction after 6 and after 12 months. The evaluation of the secondary efficacy endpoint will follow the same line as the primary outcome.
Secondary binary endpoints such as mf response will be analyzed by logistic regression with baseline mf score and time period as covariate. Separate models will be fitted for each disease and odds ratios will be reported. The secondary analyses will be performed in the mITT population.

In further, exploratory secondary analysis we will explore two-part models to test the primary estimand (Gleiss et al, 2015). Two-part models are two-component models for zero-inflated data, where one is modeling the non-zero responses and the second is modeling the non-zero response values. We apply the R-package GLMMadaptive (Rizopoulos D, 2023), where a logistic regression for the dichotomous indicator that the outcome is zero or not, and a standard linear mixed model for the logarithmic transformation of the non-zero responses is specified. In further exploratory secondary analysis a Bayesian analysis allowing for borrowing of information across indications will be explored.

Further details of the statistical analyses, and details for the secondary endpoints for *T. trichiura* will be specified in the SAP.

8.3.4. Safety Analyses

All safety analyses across all interventions in the basket trial will be descriptive and based on the Safety analysis dataset including all randomized participants who are exposed to study intervention(s). Participants will be analyzed according to the intervention(s) they actually receive.

8.3.5. Other Analyses

Any other analyses will be described in the SAP.

8.4. Sample Size Determination

General:

The overall sample size for each disease is fixed (onchocerciasis, mansonellosis, loiasis 200 each (120 each for the 1st stage and 80 each for the second stage), trichuriasis 160). With the help of simulation studies different scenarios are evaluated and resulting power values are compared for each disease. In the simulations, the significance level for each disease is 0.025 (one-sided). The assumed drop-out rate is 10%.

Sample size determination and power analysis were assessed through a simulation study. The sponsor's evaluation focused on estimating the power to select a dose during interim analysis and subsequently reject the corresponding null hypothesis in the final analysis for each disease.

In the context of mansonellosis, onchocerciasis, and loiasis, power calculation involves not only sample size and assumed effect sizes but also the dose selection rule at interim analysis and the predictivity of the 6-month endpoint for the 12-month endpoint. Three scenarios were considered: (1) efficacy only in the high dose, (2) trends in effect sizes, and (3) equal efficacy across all doses. The assumed effect size are detailed in Table 9 and are assumed to be lower at the six-month endpoint compared to the 12-month endpoint. The sponsor adopted a lognormal distribution for mf level/egg counts at baseline. Correlation between measurements at baseline, 6 months, and 12 months was assumed to be ½, with an AR(1) correlation structure. Measurements at month 6 and month 12 were assumed to follow a mixture distribution with a proportion of total responders whose mf load / egg count drops to 0 and a lognormal component, which, in mean drops by a certain rate (the reduction rate) compared to baseline. Table 9 lists the assumed effect sizes and total responder rates in the different scenarios. Variability of parasite load across the four diseases was assumed based on baseline estimates from prior trials (see Table 13) and appropriately transformed under the assumption of a log-normal distribution.

With a first-stage total sample size of N1 = 120 (160 for trichuris, resulting in 40 participants per dose), a second-stage total sample size of N2 = 80 (resulting in either 40 participants per active dose if only one active dose is selected for stage 2 or 26 participants per active dose if two active doses are selected for stage 2), a drop-out rate of 10% and a selection threshold $\alpha 1 = 0.3$ (applied to the p-values of the 6-month mf load comparisons in the interim analysis for dose selection) for mansonellosis, onchocerciasis, and loiasis, the power to detect any effective dose in the scenarios where there is a positive dose-response trend (and the highest dose has a reduction rate of 60%) ranges from 72%-98% for onchocerciasis, 73-98% for mansonellosis and 86%-100% for loiasis. If there is only an effect in the highest dose with a reduction rate of 60%, the probability that the high dose is selected, and the corresponding hypothesis is rejected is 68% for Onchocerciasis, 68% for mansonellosis and 71% for loiasis. Conditional on the event that the high dose is selected, the power is at least 95% in the different diseases. See Table 11 for the probabilities for each dose to be selected in the interim analysis. Table 9 gives the power in each of the scenarios.

For trichuriasis, in the proposed multiarm trial with three comparisons, an overall sample size of N=160 (resulting in 40 participants per dose) and a drop-out rate of 10%, power to reject any dose exceeds 90% across the considered scenarios where at least one dose has a reduction rate of 60% (Bonferroni-Holm adjustment for the three comparison with the Wilcoxon test, one-sided). Table

12 shows the assumed effect sizes, total responder rates in different scenarios, the resulting power for each dose compared to placebo and the power to detect any effective dose.

Table 9. Definition of scenarios of reduction rates and total responder rates (low dose (LD)/medium dose (MD)/high dose (HD)) for the power calculations for onchocerciasis, mansonellosis and loiasis.

The proportion of total responders at months 6 and 12 in each setting is assumed to be 20 percentage points less than the reduction rate at month 12. Throughout we assumed that also in the control group there is a proportion of 10% responders at month 6 and month 12 but no change in the lognormal component.

	Reduction rates		Total responder rates at 6 and 12 months
	6 months LD/MD/HD	12 months LD/MD/HD	LD/MD/HD
Efficacy only in high dose 1	0/0/50	0/0/60	10/10/40
Efficacy only in high dose 2	0/0/60	0/0/70	10/10/50
Trend 0	0/20/50	0/30/60	10/10/40
Trend 1	0/30/50	0/40/60	10/20/40
Trend 2	0/40/50	0/50/60	10/30/40
All doses effective	50/50/50	60/60/60	40/40/40

Table 10. Power in % for low dose (LD)/medium dose (MD)/high dose (HD), defined as the probability that the dose is selected and that the corresponding null hypothesis is rejected. In addition, the conditional power that the null hypothesis corresponding to the highest dose is rejected, conditional on the event that it is selected in the interim analysis is given in brackets. Finally, the probability that any null hypothesis is rejected is reported.

**Power in % for LD, MD, HD** *Scenarios*	*Onchocerciasis* LD/MD/HD	*Mansonellosis* LD/MD/HD	*Loiasis* LD/MD/HD
Efficacy only in high dose 1	1/1/66 (95) **68**	1/1/67 (95) **68**	1/1/70 (99) **71**
Efficacy only in high dose 2	1 /1/69 (99) **71**	1 /1/70 (100) **71**	1/1/71 (100) **72**
Trend 0	1/15/64 (92) **72**	1 /17/65 (92) **73**	1/39/70 (98) **86**
Trend 1	1/63/62 (88) **87**	1/67/63 (90) **89**	1/90/69 (98) **98**
Trend 2	1/95/62 (88) **98**	1/96/63 (90) **98**	1/100/69 (98)**100**
All doses effective	100/100/0 (-)**100**	100/100/0 (-) **100**	100/100/0 (-) **100**

Table 11. Selection probabilities in % for low dose/medium dose/high dose, which is the frequency (%) the dose is selected for/started in stage 2 for scenarios : efficacy only in the high dose, linear trend in effect sizes, and equal efficacy across all doses.

**Selection Probabilities in % for LD, MD, HD** *Scenarios*	*Onchocerciasis* LD/MD/HD	*Mansonellosis* LD/MD/HD	*Loiasis* LD/MD/HD
Efficacy only in high dose 1	30/44/70	30/45/70	30/44/70
Efficacy only in high dose 2	30/45/70	30/44/70	29/44/71
Trend 0	30/62/70	29/64/71	29/72/71
Trend 1	30/88/70	30/90/70	29/96/71
Trend 2	30/99/70	30/99/70	30/100/70
All doses effective	100/100/0	100/100/0	100/100/0

Table 12. Definition of scenarios of reduction rates and total responder rates at day 18 and resulting power in % for low dose (LD)/medium dose (MD)/high dose (HD) for trichuriasis. In addition, the probability that any null hypothesis is rejected is reported (in bold).

	Reduction Rates	*Responder Rates*	*Power each dose and any dose*
	LD/MD/HD	LD/MD/HD	LD/MD/HD
Efficacy only in high dose 1	0/0/60	10/10/40	1/1/90 **90**
Efficacy only in high dose 2	0/0/70	10/10/50	1/1/99 **99**
Trend 0	0/30/60	10/10/40	2/20/90 **91**
Trend 1	0/40/60	10/20/40	2/30/90 **91**
Trend 2	0/50/60	10/30/40	2/67/91 **93**
All doses effective 1	60/60/60	40/40/40	95/95/95 **99**
All doses effective 2	30/50/70	20/30/50	28/68/99 **99**

Table 13. Considered baseline parameters for mean and standard deviation of the raw values for each disease.

**Disease**	**mean**	**Standard deviation**	**Source**
Mansonellosis	1838	2565	Internal data
Onchocerciasis	19	30	Internal data
Loiasis	5000	4000	(Campillo et al., 2022)[a]
Trichuriasis	1000	3000	(Schneeberger et al., 2022; Welsche et al., 2023)[b]

[a]inclusion criteria: 1-15000; [b]EPG> 48, age 12-19

9. Supporting Documentation and Operational Considerations

9.1. Appendix 1: Regulatory, Ethical, and Trial Oversight Considerations

9.1.1. Regulatory and Ethical Considerations

This trial will be conducted in accordance with the protocol and with the following:

- Consensus ethical principles derived from international guidelines including the Declaration of Helsinki and Council for International Organizations of Medical Sciences (CIOMS) international ethical guidelines
- Applicable ICH Good Clinical Practice (GCP) guidelines
- Applicable laws and regulations

The protocol, protocol amendments, ICF, IB and other relevant documents (e.g., advertisements) must be submitted to an IRB/IEC and other institutions as applicable, by the investigator and reviewed and approved by the IRB/IEC before the trial is initiated.

Any amendments to the protocol will require IRB/IEC and other institutions as applicable, approval before implementation of changes made to the trial design, except for changes necessary to eliminate an immediate hazard to trial participants.

Protocols and amendments to the protocol (in some cases only substantial amendments as per country specific requirements), will require regulatory authority approval prior to initiation/implementation, except for amendments where changes are necessary to eliminate an immediate hazard to trial participants.

The investigator will be responsible for the following, as applicable:

- Providing written summaries of the status of the trial to the IRB/IEC and other institutions as applicable, annually or more frequently in accordance with the requirements, policies, and procedures established by the IRB/IEC and other institutions as applicable
- Notifying the IRB/IEC of SAEs or other significant safety findings as required by IRB/IEC and other institutions as applicable, procedures.
- Providing oversight of the conduct of the trial at the site and adherence to ICH guidelines, the IRB/IEC and other institutions as applicable, all other applicable local regulations and the principles outlined in 21 CFR or EU reg 536/2014 when these can be applied or make sense.
- Notifying the sponsor, and documenting and explaining any deviation to the approved protocol.

9.1.2. Informed Consent Process

The investigator or the investigator's representative will explain the nature of the trial, including the risks and benefits, to the potential participant or their legally authorized representative and answer all questions regarding the trial.

Potential participants must be informed that their participation is voluntary. They or their legally authorized representatives will be required to sign a statement of informed consent that meets local

regulations, ICH guidelines, privacy and data protection requirements, where applicable, and the IRB/IEC or trial center requirements.

The medical record must include a statement that written informed consent was obtained before the participant was enrolled in the trial and the date the written consent was obtained. The authorized person obtaining the informed consent must also sign the ICF.

Participants must be reconsented to the most current version of the ICF(s) during their participation in the trial (unless exceptionally justified as not-applicable, and agreed with sponsor).

A copy of the ICF(s) must be provided to the participant or their legally authorized representative.

Participants who re-enter the screening process under the circumstances listed in section 4.4.1 must sign a new informed consent form, will receive a new participant identification number and, if included, a new randomisation number

The ICF will contain a separate section that addresses the use of remaining mandatory samples for optional exploratory research. The investigator or authorized designee will explain to each participant the objectives of the exploratory research. Participants will be told that they are free to refuse to participate and may withdraw their consent at any time and for any reason during the storage period. If not included in the ICF, a separate signature will be required to document a participant's agreement to allow any remaining specimens to be used for exploratory research. Participants who decline to participate in this optional research will not provide this separate signature.

9.1.3. Recruitment strategy

Please refer to appendix 5 country specifics.

9.1.4. Data Protection

Participants will be assigned a unique identifier by sponsor. Any participant records or datasets that are transferred to sponsor will contain the identifier only; participant names or any information which would make the participant directly identifiable will not be transferred.

The participant must be informed that their personal trial-related data will be used by sponsor in accordance with relevant data protection law. Participants must be informed about the legal basis used for processing of their data (e.g. consent, legitimate basis etc). The participants will be required to give consent for their data to be used as described in the informed consent.

The participant must be informed that their medical records may be examined by Clinical Quality Assurance auditors or other authorized personnel appointed by sponsor (e.g., monitors) as described in the ICF, by appropriate IRB/IEC members, and by inspectors from regulatory authorities.

The investigator must notify any personal data breach to sponsor as soon as possible.

The contract between sponsor and trial sites and other data processors (e.g., labs, data management companies, consultants etc.) specifies responsibilities of the parties related data protection, including handling of data security breaches and respective communication and cooperation of the parties.

Information technology systems used to collect, process, and store trial-related data are secured by technical and organizational security measures designed to protect such data against accidental or unlawful loss, alteration, or unauthorized disclosure or access.

9.1.5. Committees Structure

The eWHORM Consortium is responsible for the set up and management of the trial and for any scientific decisions.

Consortium lead:

- Universitätsklinikum Bonn, [UKB], Germany

Consortium members :

- Institut National de Recherche Biomédicale, [INRB], Democratic Republic of Congo
- University of Buea, [UB], Cameroon
- Centre de Recherches Médicales de Lambaréné, [CERMEL], Gabon
- Bernhard-Nocht-Institut für Tropenmedizin, [BNITM], Germany
- Medizinische Universität Wien, [MUW], Austria responsible for the statistic
- Erasmus University Medical Center Rotterdam, Netherlands [EMC],
- EURICE EUROPEAN RESEARCH AND PROJECT OFFICE GMBH [EURICE], Germany

And the following Associated Partners

- Drugs for Neglected Diseases Initiative, [DNDi], Switzerland, responsible for the Data Management and Pharmacovigilance
- Schweizerisches Tropen- und Public Health-Institut, [Swiss TPH], Switzerland, responsible for the clinical trial on *T. trichiura* and co-monitoring

Data and Safety Monitoring Board

An independent global Data Safety Monitoring Board (DSMB) will monitor accumulating efficacy and safety data on an ongoing basis. The mission of the DSMB will be to safeguard the interests of study participants and to enhance the integrity and credibility of the trial. The DSMB will be asked to recommend stopping the study early for efficacy only when there is clear and substantial evidence of a treatment benefit. Information of membership, terms of reference, roles, responsibilities, authority, decision-making and relationships of the independent DSMB, including statistical decision principles, will be detailed in a separate DSMB charter. The DSMB charter will be finalized prior to the trial initiation.

9.1.6. Dissemination of Clinical Trial Data

Sponsor will post trial information before the first participant in, and tabular trial results (even with negative outcomes and/or from terminated development programs) on a recognized public registry.

For publication planning and other activities related to nonpromotional, peer-reviewed publications. Sponsor policy for "Scientific communication" will apply, ensuring scientific integrity and credibility of publication performed by or on behalf of sponsor.

Trial results and future development plan will be communicated to the participants of this study.

9.1.7. Data Quality Assurance

All participant data relating to the trial will be entered into electronic CRFs unless transmitted to sponsor or designee electronically (e.g., ECG or PK data). The investigator is responsible for verifying that data entries are accurate and correct by electronically signing the eCRF.

The site team will be trained on eCRF completion and the eCRF completion guide will be provided for reference.

The eCRFs are built using fully validated secure web-enabled software that conforms to 21 CFR part 11 requirements. Investigator site staff will not be given access to the electronic data capture (EDC) system until they have been trained. Automatic validation checks for data discrepancies will be programmed in the eCRFs and allow modification and/or verification of the entered data by the investigator staff.

The investigator must permit trial-related monitoring, audits, IRB/IEC review, and regulatory agency inspections and provide direct access to source documents.

Monitoring details describing strategy, including definition of trial critical data items and processes (e.g., risk-based initiatives in operations and quality such as risk management and mitigation strategies and analytical risk-based monitoring), methods, responsibilities, and requirements, including handling of noncompliance issues and monitoring techniques (central, remote, or on-site monitoring) are provided in the monitoring plan.

The sponsor or associated partners (DM, co-monitoring) are responsible for the data management of this trial, including quality checking of the data.

Sponsor assumes accountability for actions delegated to other individuals (e.g., contract research organizations).

Records and documents, including signed ICFs, pertaining to the conduct of this trial must be retained by the investigator for 25 years after trial completion unless local regulations or institutional policies require a longer retention period. No records may be destroyed during the retention period without the written approval of sponsor. No records may be transferred to another location or party without written notification to sponsor.

The investigator/institution should take measures to prevent accidental or premature destruction of these documents.

9.1.8. Source Documents

The investigator must maintain adequate and accurate source documents and trial records that include all pertinent observations on each of the site's trial participants. Source data should be attributable, legible, contemporaneous, original, accurate, and complete.

Source documents provide evidence for the existence of the participant and substantiate the integrity of the data collected. Source documents are filed at the investigator's site.

The verification of the eCRF data must be by direct inspection of source documents. Definition of what constitutes source data, and its origin can be found in monitoring guidelines.

Data entered in the eCRF or PV forms that are transcribed from source documents must be consistent with the source documents or the discrepancies must be explained. The investigator may need to request previous medical records or transfer records, depending on the trial. Also, current medical records must be available.

The monitor or designee will perform monitoring to confirm that data entered into the CRF by authorized site personnel are accurate, complete, and verifiable from source documents; that the safety and rights of participants are being protected; and that the trial is being conducted in accordance with the currently approved protocol and any other trial agreements, ICH GCP, and all applicable regulatory requirements.

9.1.9. Trial and Site Start and Closure

The trial start date is the date on which the clinical trial will be opened by the sponsor for recruitment of participants.

Trial/Site Termination

Sponsor or designee reserves the right to close the trial site or terminate the trial. Trial sites will be closed upon trial completion. A trial site is considered closed when all required documents and trial supplies have been collected and a trial-site closure visit has been performed.

The investigator may initiate trial-site closure at any time, provided there is reasonable cause and sufficient notice is given in advance of the intended termination.

Reasons for the early closure of a trial site by sponsor or investigator may include but are not limited to:

For trial termination:

- Discontinuation of further IP development

For site termination:

- Failure of the investigator to comply with the protocol, the requirements of the IRB/IEC or local health authorities, sponsor's procedures, or GCP guidelines.
- Inadequate or no recruitment (evaluated after a reasonable amount of time) of participants by the investigator.
- Total number of participants included earlier than expected.

If the trial is prematurely terminated or suspended, sponsor shall promptly inform the investigators, the IECs/IRBs, the regulatory authorities, and any contract research organization(s) used in the trial of the reason for termination or suspension, as specified by the applicable regulatory requirements. The investigator shall promptly inform the participant and should assure appropriate participant therapy and/or follow-up.

9.1.10. Publication Policy

- The results of this trial may be published or presented at scientific meetings. If this is foreseen, the investigator agrees to submit all manuscripts or abstracts to sponsor before submission. This allows sponsor to protect proprietary information and to provide comments.
- Sponsor will comply with the requirements for publication of trial results. In accordance with standard editorial and ethical practice, sponsor will generally support publication of multicenter trials only in their entirety and not as individual site data.
- Authorship will be determined by mutual agreement and in line with International Committee of Medical Journal Editors authorship requirements.
- A refined publication strategy will be developed jointly by all partners. The consortium will publish in Open Access journals and at international conferences.

9.1.11. Insurance

Sponsor will cover any claims made by a participant, or by a family member or legal representative on his/her behalf, for damage resulting directly from the trial and will also indemnify the investigator against any liability resulting from such claims. Coverage of the damage to the participant and indemnification of the investigator by sponsor will not apply if the damages to the participant result from negligence or intentional misconduct on the part of employees or agents of the investigational centre(s). Such claims will be covered by insurance contracted by the investigational centre(s).

9.1.12. Funding

This project (No 101103053) is supported by the Global Health EDCTP3 Joint Undertaking and its members as well as the Swiss Confederation.

9.2. Appendix 2: Clinical Laboratory Tests

The tests detailed in Table 14 will be performed by the local laboratory.

Additional tests may be performed at any time during the trial as determined necessary by the investigator, in case of abnormalities (including in liver function or hematological tests as described in Appendix 6), or required by local regulations.

Table 14. Protocol-required Safety Laboratory Tests

Laboratory Tests	Parameters	
**Hematology**	• Platelet count<br>• Red blood cell (RBC) count<br>• White blood cell (WBC) count with differential:<br><br><br><br>• Basophils<br>• Hemoglobin<br>• Hematocrit	 <br>- Neutrophils<br>- Lymphocytes<br>- Monocytes<br>- Eosinophils
**Clinical chemistry**	• Blood urea nitrogen (BUN)<br>• Potassium<br>• Creatinine<br>• Sodium<br>• Calcium<br>• Glucose	• Aspartate aminotransferase (AST)/serum glutamic-oxaloacetic transaminase (SGOT)<br>• Alanine aminotransferase (ALT)/serum glutamic-pyruvic transaminase (SGPT)<br>• Gamma glutamyl transpeptidase (gamma GT)<br>• Alkaline phosphatase<br>• Total and direct bilirubin<br>• Total protein<br>• C-reactive protein
**Routine urinalysis**	• Specific gravity<br>• pH, glucose, protein, blood, ketones, bilirubin, urobilinogen, nitrite, leukocyte esterase by dipstick<br>• Microscopic examination (if blood or protein is abnormal)	
**Pregnancy testing**	• Highly sensitive serum or urine human chorionic gonadotropin (hCG) pregnancy test (as needed for women of childbearing potential)	

Investigators must document their review of each laboratory safety report.

9.3. Appendix 3: AEs and SAEs: Definitions and Associated Instructions

9.3.1. Definition of AE

AE Definition

An AE is any untoward medical occurrence in a clinical trial participant, to whom an IP is administered, and which does not necessarily have a causal relationship with this treatment.

An AE can therefore be any unfavorable and unintended sign (e.g., an abnormal laboratory finding), symptom, or disease temporally associated with the use of a medicinal product IP whether or not considered related to this IP.

NOTE: Definition of an AE includes worsening (in severity and/or frequency) of pre-existing conditions ("Medical history") before the first IP administration and/or abnormalities of procedures (i.e., ECG, X-ray…) and/or laboratory results which are assessed as "clinically significant".

Screening Period:

Medical occurrences/conditions that begin before the start of trial treatment but after obtaining informed consent will be recorded as medical history/current medical conditions (not as AEs), except if the events could be caused to specific circumstances in which the participant has been placed (e.g. withdrawal of a previous drug to enter in the clinical trial or "protocol related").

Such event is not considered as AE *per se*, but for monitoring purpose, any non-serious "event" to be reported after signature of the ICF should be reported on an "AE form".

Events Meeting the AE Definition

- Any abnormal laboratory test results (hematology, clinical chemistry, or urinalysis) or other safety assessments (e.g., ECG, radiological scans, vital signs measurements…), including those that worsen from baseline, considered "clinically significant" in the medical and scientific judgment of the investigator (i.e., not related to progression of underlying disease, or more severe than expected for the participant's condition)

  Guidance on the assessment of clinical significance of laboratory/procedures abnormalities:

  Laboratory/procedures abnormalities (or worsening in severity or frequency of pre-existing abnormalities) should be assessed as "clinically significant" (and therefore have to be reported as an AE) if they meet AT LEAST ONE of the following conditions:

  - The abnormality suggests a disease and/or organ toxicity AND this abnormality was not present at the screening visit or is assessed as having evolved since the screening visit
  - The abnormality results in discontinuation of the trial drug
  - The abnormality requires medical intervention or concomitant therapy.

  Note: It means an abnormal laboratory result should be considered as « clinically significant » if this abnormality (=outside normal values) is assessed as « significant », even in the absence of signs and symptoms. Investigators should attempt to establish a

diagnosis of the event based on signs/symptoms and other clinical information/tests. When all necessary information to confirm a medical diagnostic will be available, then only , a syndromic clinical diagnosis should be recorded rather than the abnormal value itself (or signs/symptoms) used in initial notification, (e.g., 'acute pancreatitis' instead of each finding separately: 'high levels of amylase', 'high levels of lipase', 'abdominal pain and vomiting'; or 'anaemia' rather than 'decreased red blood cell count').

- Exacerbation of a chronic or intermittent pre-existing condition ("Medical history") before first IP administration including either an increase in frequency and/or intensity of the condition
- New condition detected or diagnosed after IP administration even though it may have been present before the start of the trial
- Signs, symptoms, or the clinical sequelae of a suspected drug-drug interaction
- Signs, symptoms, or the clinical sequelae of a suspected overdose of either IP or a concomitant medication. Overdose per se will not be reported as an AE/SAE unless it is an intentional overdose taken with possible suicidal/self-harming intent. Such overdoses should be reported regardless of sequelae.
- **Lack of efficacy or failure of expected pharmacological action per se will not be reported as an AE or SAE**. Such instances will be captured in the efficacy assessments. However, the signs, symptoms, and/or clinical sequelae resulting from lack of efficacy will be reported as AE or SAE if they fulfil the definition of an AE or SAE.

Events not Meeting the AE Definition

- Any abnormal laboratory findings or other abnormal safety assessments that are associated with the underlying disease, unless judged by the investigator to be more severe than expected for the participant's condition
- The disease/disorder being studied or expected progression, signs, or symptoms of the disease/disorder being studied, unless more severe than expected for the participant's condition
- Medical or surgical procedure (e.g., endoscopy, appendectomy): the condition that leads to the procedure is the AE
- Situations in which an untoward medical occurrence did not occur (social and/or convenience admission to a hospital)
- Anticipated day-to-day fluctuations of pre-existing disease(s) or condition(s) present or detected at the start of the trial that do not worsen

9.3.2. Definition of SAE

An SAE is defined as any untoward medical occurrence that, at any dose, meets one or more of the criteria listed:

a. Results in death

i.e., causes or contributes to the death.

b. Is life-threatening

The term *life threatening* in the definition of *serious* refers to an event in which the participant was at risk of death at the time of the event. It does not refer to an event, which hypothetically might have caused death, if it were more severe.

c. Requires inpatient hospitalization or prolongation of existing hospitalization

i.e., the AE requires at least an overnight admission or prolongs a hospitalisation beyond the expected length of stay. Hospital admissions for surgery planned before trial entry, for social reasons, for any elective surgery (i.e., plastic surgery) or for normal disease management (including treatment adjustment) are NOT to be considered as SAE according to this criterion (i.e., if the protocol or the standard management of the disease under trial requires planned hospitalisation).

d. Results in persistent or significant disability/incapacity

i.e., the AE resulted in a substantial disruption of the participant´s ability to conduct normal activities.

e. Is a congenital anomaly/birth defect

i.e., an AE outcome in a child or foetus of a participant exposed to the IP before conception or during pregnancy.

f. Is an important medical event, i.e. medically significant:

- Medical or scientific judgment should be exercised by the investigator in deciding whether other situations should be considered serious, such as important medical events that might not be immediately life-threatening or result in death or hospitalization but may jeopardize the participant or might require medical or surgical intervention to prevent one of the other outcomes listed above.
    - Examples of such events include invasive or malignant cancers, intensive treatment in an emergency room or at home for allergic bronchospasm, blood dyscrasias, convulsions not resulting in hospitalization, or development of IP dependency or IP abuse.
- In addition, any suspected transmission via a medicinal product of any infectious agent is also considered as serious.

9.3.3. Recording and Follow-Up of AE and/or SAE

AE and SAE Recording

The investigator is obligated to perform or arrange for the conduct of supplemental measurements and/or evaluations as medically indicated or as requested by sponsor to elucidate the nature and/or causality of the AE or SAE as fully as possible. This may include additional laboratory tests or investigations, histopathological examinations, or consultation with other health care professionals.

If a participant dies during participation in the study or during a recognized follow-up period, the investigator will provide sponsor with a copy of any postmortem findings including histopathology.

When an AE/SAE occurs, it is the responsibility of the investigator to review all documentation (e.g., hospital progress notes, laboratory reports, and diagnostics reports) related to the event.

The investigator will then record all relevant AE/SAE information.

It is **not** acceptable for the investigator to send photocopies of the participant's medical records to sponsor in lieu of completion of the sponsor required form.

There may be instances when copies of medical records for certain cases are requested by sponsor. In this case, all participant identifiers, with the exception of the participant number, will be redacted on the copies of the medical records before submission to sponsor.

Assessment of Severity (Intensity):

For each serious and non-serious AE, the investigator is required to assess the severity of each adverse event.

It is to be noted the distinction between severity (intensity) and seriousness of AEs. A severe AE is not necessarily a serious AE.

The severity is a clinical **determination of the intensity** of a specific event. The severity of AEs must be assessed by investigators using the National Cancer Institute's Common Terminology Criteria for Adverse Events (CTCAE) version 5.0 (see Appendix 8), as a guide in the grading of the severity of AEs.

In case of AEs that are not described in the CTCAE version 5.0 AE severity grading system, the investigator will use the terminology mild, moderate, severe, life-threatening or death to describe the maximum severity of the AE as follows:

Mild:	**An AE that is easily tolerated by the participant, causes minimal discomfort and does not interfere with everyday activities.**
Moderate:	**An AE that is sufficiently discomforting to interfere with normal everyday activities; intervention may be needed.**
Severe:	**An AE that prevents normal everyday activities; treatment or other intervention usually needed.**
Life-Threatening:	**Participant is at significant risk of life; urgent intervention required. It does not refer to an event which *hypothetically* might have caused death if it were more severe**
Death:	**Death related to an event.**

When the intensity of an AE changes over time, each change in intensity will be recorded in the source documents until the event resolves. However, only one AE and the maximum intensity will

be recorded in the CRF for each separate event. If the AE resolves but then recurs, each will be recorded as a separate AE, with the appropriate start and stop times.

Assessment of Causality

- **Causality Assessment made by the Investigator:**

For each serious and non-serious adverse event, the investigator is required to assess the possible relationship between the adverse event and the study drug(s)/IP(s) i.e., to determine whether there exists a reasonable possibility that the study drug caused or contributed to the adverse event. This means that there are facts (evidence) or arguments to suggest a causal relationship. "The investigator will use clinical judgment to determine the relationship".

The causality assessment of an AE to the investigational medicinal product will be rated as follows:

Not related:	There is no reasonable possibility of causal relationship.
Related:	There is at least a reasonable possibility of a causal relationship between an adverse event and an investigational medicinal product. This means that there are facts (evidence) or arguments to suggest a causal relationship.

To help investigators with the decision binary tree (i.e., Related/Not related) in the evaluation of causality, the Council for International Organizations of Medical Sciences (CIOMS VI) group recommends that investigators be asked to consider the following before reaching a decision:

- Medical history (including presence of risk factors)
- Lack of efficacy/worsening of existing condition
- Study medications
- Other medications (concomitant or previous)
- Withdrawal of study medication, especially following trial discontinuation / end of clinical trial
- Erroneous treatment with study medication (or concomitant)
- Protocol-related procedure

For causality assessment, the investigator will also consult the IB and/or product information, for marketed products.

The investigator must document these assessments of causality in the medical notes There may be situations in which an SAE has occurred and the investigator has minimal information to include in the initial report to sponsor. However, it is very important that the investigator always make an assessment of causality for every event before the initial transmission of the SAE data to sponsor.

The investigator may change their opinion of causality in light of follow-up information and send an SAE follow-up report with the updated causality assessment.

The causality assessment is one of the criteria used when determining regulatory reporting requirements.

9.4. Appendix 4: Contraceptive and Barrier Guidance

Highly effective method of contraception: “methods that can achieve a failure rate of less than 1% per year when used consistently and correctly are considered as highly effective birth control methods.” Such as:

- combined (estrogen and progestogen containing) hormonal contraception associated with inhibition of ovulation:
    - oral
    - intravaginal
    - transdermal
- progestogen-only hormonal contraception associated with inhibition of ovulation:
    - oral
    - injectable
    - implantable
- intrauterine device (IUD)
- intrauterine hormone-releasing system (IUS)
- bilateral tubal occlusion
- vasectomised partner
- sexual abstinence, only if part of their usual and preferred lifestyle.

(Clinical Trials Facilitation and Coordination Group, 2020)

In this trial, preferably methods with low user dependency should be used.

The investigator should evaluate the potential for contraceptive method failure (eg, noncompliance, recently initiated) in relationship to the first dose of study intervention.

9.5. Appendix 5: Country-specific Requirements

**Appendix 5 version number/date:**	v4.0/12 April 2025
**Country:**	Tanzania (TZA)
**Sponsor:**	Swiss Tropical and Public Health Institute (Swiss TPH), Allschwil, Switzerland
**Clinical Trial Site:**	Public Health Laboratory Ivo de Carneri (PHL IdC), Chake Chake, Pemba, Tanzania

Country-specific additions and clarifications to the respective sections of the master protocol are detailed below:

Section 1. Introduction

Section 1.3. Benefit/Risk Assessment.

- The following additional steps will be taken by the clinical trial site to minimize potential risks.
- Participants will be kept under close supervision during the dosing period(s) of study treatment, to help ensure adherence to the protocol.
- Adequate medical surveillance will be assured during the dosing period(s), and provisions made for medical care, or referral, in case of emergency.

Section 2. Objectives, Endpoints, and, Estimands

- Exploratory objective: Due to the molecular assay for soil-transmitted helminth (STH) infections still being in development, PCR/LAMP (Loop-mediated Isothermal Amplification) testing for helminth infections from stool samples will not be conducted.

Section 5. Blinding, Masking

- This section of the master protocol describes the blinding of study team members throughout the duration of the study. As a clarification, for this part of the basket study conducted in Pemba, Tanzania (the sub-study in trichuriasis), the blinding described will be for the duration of the study in Tanzania.

Section 4. Clinical Trial Population

- In the Pemba, Tanzania site, the primary disease targeted for participant recruitment in this trial is trichuriasis. During screening, the disease-specific inclusion criteria for trichuriasis (Section 4.1) will be applied.
- Participants will not be queried regarding alcohol consumption, as it is not relevant to the trial population.

Section 7. Clinical Trial Assessments and Procedures

- No diagnosis of *Mansonella perstans*, *Loa loa* and *Onchocerca volvulus* will be performed.
- Blood and urine sample collection for safety laboratory test D3 (post-dose, *T. trichiura*) will not be performed.
- The secondary endpoint of pharmacokinetics characteristics of oxfendazole will not be assessed. PK samples will not be taken.

- The re-treatment with Albendazole (400 mg on three consecutive days) will be done for all participants at Day 47 post-dose, which is the last day of follow-up for women of child bearing potential (WOCBP).
- The trichuriasis trial in Pemba has its own schedule of events (see below):

Screening: Month -3 to Day -1

- Information session and informed consenting
- Collection of demographic data
- Collection of two stool samples on two consecutive days for parasitological assessment

Baseline: Day -7 to Day -1

- Collection of medical history
- Collection of concomitant treatments
- Vital signs (pulse, respiratory rate, blood pressure, temperature, weight, and height)
- Complete physical examination and medical symptoms
- Neurological examination
- Blood sample collection for safety laboratory test (no electrolytes (potassium, sodium, calcium) will be tested)
- Urine sample collection for safety laboratory test
- Verification of last menstrual period, and confirmation of contraceptive method used, evaluation of contraceptive method and potential for failure.

Randomization and Pre-Dose: Day 1

- Pregnancy test in women of child bearing potential (WOCBP) (highly sensitive urine test). <u>Note this must be within 24 hours of 1st dose of IP</u>
- Vital signs (pulse, respiratory rate, blood pressure, temperature)
- Symptom directed physical examination and baseline medical symptoms
- Assessment of eligibility (Inclusion and Exclusion Criteria)
- Randomization

Treatment: Day 1 to Day 3

- Treatment administration on 3 consecutive days
- Vital signs (pulse, respiratory rate, blood pressure, temperature) within 3h after each dose
- Collection of Adverse Events 3h and 24h after each dose

Follow-up: Day 18 post-dose

- Collection of concomitant treatments
- Collection of Adverse Events
- Vital signs (pulse, respiratory rate, blood pressure, temperature)
- Complete physical examination
- Neurological examination
- Blood sample collection for safety laboratory test (no electrolytes (potassium, sodium, calcium) will be tested)
- Urine sample collection for safety laboratory test
- Pregnancy test in WOCBP (highly sensitive urine or serum test), verification of last menstrual period, and evaluation of contraceptive method and potential for failure
- Collection of two stool samples on two consecutive days for parasitological assessment

Follow-up: Day 47 post-dose

- Pregnancy test in WOCBP (highly sensitive urine or serum test) and verification of last menstrual period
- Collection of Adverse Events in WOCBP

Section 9. Supporting Documentation and Operational Considerations

Section 9.1.3. Recruitment:

- The trial will be implemented as community-based study in communities with endemicity of *T. trichiura* infection above 20% identified during previous trials.
- Potential participants will be invited to participate in an information session, which will take place in their villages.
- The research team will explain the purpose and procedures of the study, as well as potential benefits and risks of participation.
- Attendees will be encouraged to ask questions which will be discussed in an open setting. Potential participants in the study will be invited to complete the process of informed consent by signing the informed consent form (ICF).
- Participants having a signed ICF will be assessed for eligibility during screening procedures.

Section 9.1.4. Data Protection:

- No images from which a subject can be visually identified will be transmitted outside of the clinical trial site.

Other Country-Specific Information:

Study Duration and Site

- The clinical trial duration for each participant will be up to Day 18 (47 days post last dose for WOCBP). The total duration of the study, inclusive of the screening phase, is estimated to be four months.
- The sites for this community-based study in Pemba will include Wesha and Ndagoni in Chake-Chake district.

Declaration of conflict of Interest, Privacy/Confidentiality

- The investigators of the Pemba study agree that:
  All participant data collected during this study will be treated with the highest level of confidentiality. Personal identifying information will be anonymized and stored in secure, password-protected databases. Access to data will be restricted to authorized study personnel only, and all data handling will comply with applicable data protection laws and ethical guidelines.
  They declare that there is no conflict of interest regarding the design, conduct, or publication of this study.

Sponsor and Clinical Trial Site Contacts

A separate Trial Contact List is maintained below, where names and contact details of the clinical trial site, investigators, clinical laboratories and other technical departments and/or institutions involved in the trial are listed.

Clinical Trial Contact List

Sponsor: Swiss Tropical and Public Health Institute
Country: Switzerland
Protocol: OXF-01
Title: A phase-IIa, multi-country, randomized, placebo-controlled, double-blinded, adaptive, basket trial to assess the efficacy and safety of oxfendazole in adults with trichuriasis, and /or mansonellosis, and/or onchocerciasis and/or loiasis

----

Sponsor

Swiss Tropical and Public Health Institute
Kreuzstrasse 2, 4123 Allschwil
Switzerland

Sponsor's Representative:
Prof. Dr. Jennifer Keiser
Swiss Tropical and Public Health Institute
Kreuzstrasse2, 4123 Allschwil
Switzerland

Sponsor's Medical Responsible:
Prof. Dr. Daniel Paris
Swiss Tropical and Public Health Institute
Kreuzstrasse2, 4123 Allschwil
Switzerland

Manufacture of Investigational Product:
Syngene International Ltd.,
Biocon Park, SEZ,
Bommasandra Industrial Area - Phase-IV
Bommasandra-Jigani Link Road,
Bangalore 560 099
India

Packaging and labelling of Investigational Product:
Bilcare Ltd.,
1028 Shiroli, Rajgurunagar,
Pune – 410505
India

Data Management:
Drugs for Neglected Diseases initiative (DNDi),
One Padmore Place, 8th Floor,
George Padmore Road, Kilimani,
Nairobi,
Kenya

Statistical Analysis:
Medizinische Universität Wien
Spitalgasse 23, 1090 Wien,
Austria

Clinical Trial Site

Clinical Trial Site:
Public Health Laboratory Ivo de Carneri,
P.O. Box 122, Wawi, Chake Chake, Pemba
Tanzania

Clinical Laboratory:
Public Health Laboratory Ivo de Carneri,
P.O. Box 122, Wawi, Chake Chake, Pemba
Tanzania

Co-Principal Investigator:
Said Mohammed Ali, MSc
Public Health Laboratory Ivo de Carneri
+255 242 452 003

Study Physician:
Dr. Ibrahim Said Mohammed
Ministry of Health Pemba
Phone: +255 772 550 531

Co-Principal Investigator:
Annina Schnoz, MSc
Swiss Tropical and Public Health Institute
+41 61 284 92 86

9.6. Appendix 6: Suggested Actions and Follow-up Assessments [and IP Restart/Rechallenge Guidelines]

9.6.1. Liver Safety Management

Based on published criteria (Guidance for Industry: Drug-Induced Liver Injury, DILI (U.S. Department of Health and Human Services Food and Drug Administration, 2009), discontinuation of IMP administration should be considered if any of the following criteria are met:

- ALT or AST >8xULN
- ALT or AST >5xULN for more than 2 weeks
- ALT or AST >3xULN and (Total Bilirubin (TBL) >2xULN or prothrombin time INR >1.5)
- ALT or AST >3xULN with the appearance of fatigue, nausea, vomiting, right upper quadrant pain or tenderness, fever, rash, and/or eosinophilia (>5%)

Liver enzymes will be repeated as soon as possible after the initial abnormality is identified. After that, the participant should be followed every 96 hours (4 days), or the closest possible interval, until the serum ALT and/or AST falls below 3xULN or stabilizes.

All data on liver safety will be presented to the DSMB for review.

If a participant develops hepatotoxicity, the following measures will be instituted:

- Capture information pertinent to DILI in specific case report form
- The results will be confirmed by repeating the test results, and other causes of hepatitis will be sought
- The participant will be asked to return regularly (approximately every 48-72 hours, at the most every 96 hours) for monitoring of symptoms and clinical chemistry data
- Dechallenge (Stopping of IMP administration)
- A DILI expert will be consulted to follow the participant with us.
- If warranted, the participant will be admitted a general hospital for monitoring and for any necessary intervention.

9.6.2. Hematologic Safety Management

Monitoring Parameters

- **Complete Blood Count (CBC)**: Monitoring of CBC per protocol is crucial, with a focus on white blood cell count, hemoglobin, hematocrit, platelets, and differential count.
- The DSMB will regularly review safety data, particularly focusing on hematologic events

IMP administration should be discontinued in case of any of the following parameters:

- WBC count below 2.0 x 10^9/L or
- Hemoglobin level below 7.0 g/dL or
- Platelet count below 50 x 10^9/L.

In this case the results will be confirmed by repeating the test results, and other causes of the abnormality will be sought.

Subsequent regular monitoring is essential, weekly or bi-weekly.

9.7. Appendix 7: Protocol Amendment History

Not applicable for the current version of the protocol. To be completed in the event of a protocol amendment.

The Protocol Amendment Summary of Changes Table for the current amendment is located directly before the table of contents (TOC).

Amendment [amendment number]: ([date])

This amendment is considered to be [substantial/non substantial] based on the criteria set forth in [local regulation].

Overall Rationale for the Amendment

[Rationale]

Section # and Name	Description of Change	Brief Rationale

9.8. Appendix 8: Adverse Event Severity Grading

Common Terminology Criteria for Adverse Events (CTCAE) version 5.0 (U.S. National Institutes of Health National Cancer Institute, 2017)

https://ctep.cancer.gov/protocoldevelopment/electronic_applications/docs/CTCAE_v5_Quick_Reference_8.5x11.pdf

10. References

An, G., Murry, D. J., Gajurel, K., Bach, T., Deye, G., Stebounova, L. V., Codd, E. E., Horton, J., Gonzalez, A. E., Garcia, H. H., Ince, D., Hodgson-Zingman, D., Nomicos, E. Y. H., Conrad, T., Kennedy, J., Jones, W., Gilman, R. H., & Winokur, P. (2019). Pharmacokinetics, Safety, and Tolerability of Oxfendazole in Healthy Volunteers: a Randomized, Placebo-Controlled First-in-Human Single-Dose Escalation Study. *Antimicrobial Agents and Chemotherapy*, *63*(4). https://doi.org/10.1128/AAC.02255-18

Bach, T., Galbiati, S., Kennedy, J. K., Deye, G., Nomicos, E. Y. H., Codd, E. E., Garcia, H. H., Horton, J., Gilman, R. H., Gonzalez, A. E., Winokur, P., & An, G. (2020). Pharmacokinetics, Safety, and Tolerability of Oxfendazole in Healthy Adults in an Open-Label Phase 1 Multiple Ascending Dose and Food Effect Study. *Antimicrobial Agents and Chemotherapy*, *64*(11). https://doi.org/10.1128/AAC.01018-20

Bach, T., Murry, D. J., Stebounova, L. V., Deye, G., Winokur, P., & An, G. (2021). Population Pharmacokinetic Model of Oxfendazole and Metabolites in Healthy Adults following Single Ascending Doses. *Antimicrobial Agents and Chemotherapy*, *65*(4). https://doi.org/10.1128/AAC.02129-20

Basáñez, M.-G., Pion, S. D., Boakes, E., Filipe, J. A., Churcher, T. S., & Boussinesq, M. (2008). Effect of single-dose ivermectin on Onchocerca volvulus: a systematic review and meta-analysis. *The Lancet Infectious Diseases*, *8*(5), 310–322. https://doi.org/10.1016/S1473-3099(08)70099-9

Bauters, T. (2019). Clinically Relevant Drug Interactions in HSCT. In *The EBMT Handbook* (pp. 229–235). Springer International Publishing. https://doi.org/10.1007/978-3-030-02278-5_31

Beck, G. (2023). Modification of Diet in Renal Disease (V4) [Dataset]. In *NIDDK Central Repository*. https://doi.org/10.58020/h754-eg50

Boussinesq, M. (2006). Loiasis. *Annals of Tropical Medicine & Parasitology*, *100*(8), 715–731. https://doi.org/10.1179/136485906X112194

Boussinesq, M., Gardon, J., Gardon-Wendel, N., & Chippaux, J.-P. (2003). Clinical picture, epidemiology and outcome of Loa-associated serious adverse events related to mass ivermectin treatment of onchocerciasis in Cameroon. *Filaria Journal*, *2*(Suppl 1), S4. https://doi.org/10.1186/1475-2883-2-S1-S4

Buell, K. G., Whittaker, C., Chesnais, C. B., Jewell, P. D., Pion, S. D. S., Walker, M., Basáñez, M.-G., & Boussinesq, M. (2019). Atypical Clinical Manifestations of Loiasis and Their Relevance for Endemic Populations. *Open Forum Infectious Diseases*, *6*(11). https://doi.org/10.1093/ofid/ofz417

Campillo, J. T., Bikita, P., Hemilembolo, M., Louya, F., Missamou, F., Pion, S. D. S., Boussinesq, M., & Chesnais, C. (2022). Safety and Efficacy of Levamisole in Loiasis: A Randomized, Placebo-controlled, Double-blind Clinical Trial. *Clinical Infectious*

Diseases : An Official Publication of the Infectious Diseases Society of America, *75*(1), 19–27. https://doi.org/10.1093/cid/ciab906

Chesnais, C. B., Takougang, I., Paguélé, M., Pion, S. D., & Boussinesq, M. (2017). Excess mortality associated with loiasis: a retrospective population-based cohort study. *The Lancet Infectious Diseases*, *17*(1), 108–116. https://doi.org/10.1016/S1473-3099(16)30405-4

Clinical Trial Facilitation Group. (2014). *Recommendations related to contraception and pregnancy testing in clinical trials*. https://www.hma.eu/fileadmin/dateien/Human_Medicines/01-About_HMA/Working_Groups/CTFG/2014_09_HMA_CTFG_Contraception.pdf?fbclid=IwAR3AY5Ha0ESDyqIBeUaYI9VTFWmx9bbt8NZ-80N-5ME6pkBb1UHvFsTwqlQ

Clinical Trials Facilitation and Coordination Group. (2020, September 21). *Recommendations related to contraception and pregnancy testing in clinical trials Version 1.1*. https://www.hma.eu/fileadmin/dateien/Human_Medicines/01-About_HMA/Working_Groups/CTFG/2020_09_HMA_CTFG_Contraception_guidance_Version_1.1_updated.pdf

Codd, E. E., Ng, H. H., McFarlane, C., Riccio, E. S., Doppalapudi, R., Mirsalis, J. C., Horton, R. J., Gonzalez, A. E., Garcia, H. H., & Gilman, R. H. (2015). Preclinical Studies on the Pharmacokinetics, Safety, and Toxicology of Oxfendazole. *International Journal of Toxicology*, *34*(2), 129–137. https://doi.org/10.1177/1091581815569582

El- Makawy, A., Radwan, H. A., Ghaly, I. S., & Abd El-Raouf, A. (2006). Genotoxical, teratological and biochemical effects of anthelmintic drug oxfendazole Maximum Residue Limit (MRL) in male and female mice. *Reproduction Nutrition Development*, *46*(2), 139–156. https://doi.org/10.1051/rnd:2006007

European Medicines Agency. (1997). *COMMITTEE FOR VETERINARY MEDICINAL PRODUCTS Oxfendazole Summary report 3*. https://www.ema.europa.eu/en/documents/mrl-report/oxfendazole-summary-report-3-committee-veterinary-medicinal-products_en.pdf

European Medicines Agency. (2004). *COMMITTEE FOR MEDICINAL PRODUCTS FOR VETERINARY USE FENBENDAZOLE (Extrapolation to all ruminants) SUMMARY REPORT (4)*. https://www.ema.europa.eu/en/documents/mrl-report/fenbendazole-extrapolation-all-ruminants-summary-report-4-committee-veterinary-medicinal-products_en.pdf

Fuchs, R. (n.d.). *Oxfendazole*. Retrieved March 27, 2024, from https://www.inchem.org/documents/jecfa/jecmono/v29je05.htm

Gardon, J., Gardon-Wendel, N., Demanga-Ngangue, Kamgno, J., Chippaux, J.-P., & Boussinesq, M. (1997). Serious reactions after mass treatment of onchocerciasis with ivermectin in an area endemic for Loa loa infection. *The Lancet*, *350*(9070), 18–22. https://doi.org/10.1016/S0140-6736(96)11094-1

Gelbe Liste. (2019). *CYP1A2*. https://www.gelbe-liste.de/arzneimitteltherapiesicherheit/cyp-interaktionen/cyp1a2

Gleiss, A., Dakna, M., Mischak, H., & Heinze, G. (2015). Two-group comparisons of zero-inflated intensity values: the choice of test statistic matters. *Bioinformatics*, *31*(14), 2310–2317. https://doi.org/10.1093/bioinformatics/btv154

Gonzalez, A. E., Codd, E. E., Horton, J., Garcia, H. H., & Gilman, R. H. (2019). Oxfendazole: a promising agent for the treatment and control of helminth infections in humans. *Expert Review of Anti-Infective Therapy*, *17*(1), 51–56. https://doi.org/10.1080/14787210.2018.1555241

Hallesy, D. W., Shott, L. D., & Hill, R. (1976). *Two-week Oral Toxicity Study with RS 8858 (Oxfendazole) Formulation Using Dog. Unpublished Report No. 39-D-75-8858-PO-TX from Syntex Inc. Submitted to WHO by Syntex Inc.*

Hoerauf, A., Specht, S., Büttner, M., Pfarr, K., Mand, S., Fimmers, R., Marfo-Debrekyei, Y., Konadu, P., Debrah, A. Y., Bandi, C., Brattig, N., Albers, A., Larbi, J., Batsa, L., Taylor, M. J., Adjei, O., & Büttner, D. W. (2008). Wolbachia endobacteria depletion by doxycycline as antifilarial therapy has macrofilaricidal activity in onchocerciasis: a randomized placebo-controlled study. *Medical Microbiology and Immunology*, *197*(3), 295–311. https://doi.org/10.1007/s00430-007-0062-1

Hotez, P. J., Brindley, P. J., Bethony, J. M., King, C. H., Pearce, E. J., & Jacobson, J. (2008). Helminth infections: the great neglected tropical diseases. *Journal of Clinical Investigation*, *118*(4), 1311–1321. https://doi.org/10.1172/JCI34261

Hübner, M. P., Martin, C., Specht, S., Koschel, M., Dubben, B., Frohberger, S. J., Ehrens, A., Fendler, M., Struever, D., Mitre, E., Vallarino-Lhermitte, N., Gokool, S., Lustigman, S., Schneider, M., Townson, S., Hoerauf, A., & Scandale, I. (2020). Oxfendazole mediates macrofilaricidal efficacy against the filarial nematode Litomosoides sigmodontis in vivo and inhibits Onchocerca spec. motility in vitro. *PLOS Neglected Tropical Diseases*, *14*(7), e0008427. https://doi.org/10.1371/journal.pntd.0008427

Jourdan, P. M., Lamberton, P. H. L., Fenwick, A., & Addiss, D. G. (2018). Soil-transmitted helminth infections. *The Lancet*, *391*(10117), 252–265. https://doi.org/10.1016/S0140-6736(17)31930-X

Kelly-Hope, L. A., Hemingway, J., Taylor, M. J., & Molyneux, D. H. (2018). Increasing evidence of low lymphatic filariasis prevalence in high risk Loa loa areas in Central and West Africa: a literature review. *Parasites & Vectors*, *11*(1), 349. https://doi.org/10.1186/s13071-018-2900-y

Kim, Y. E., Stolk, W. A., Tanner, M., & Tediosi, F. (2017). Modelling the health and economic impacts of the elimination of river blindness (onchocerciasis) in Africa. *BMJ Global Health*, *2*(2), e000158. https://doi.org/10.1136/bmjgh-2016-000158

Klinglmueller, F., Posch, M., & Koenig, F. (2014). Adaptive graph-based multiple testing procedures. *Pharmaceutical Statistics*, *13*(6), 345–356. https://doi.org/10.1002/pst.1640

Kyu, H. H., Abate, D., Abate, K. H., Abay, S. M., Abbafati, C., Abbasi, N., Abbastabar, H., Abd-Allah, F., Abdela, J., Abdelalim, A., Abdollahpour, I., Abdulkader, R. S., Abebe, M., Abebe, Z., Abil, O. Z., Aboyans, V., Abrham, A. R., Abu-Raddad, L. J., Abu-Rmeileh, N. M. E., … Murray, C. J. L. (2018). Global, regional, and national disability-adjusted life-years (DALYs) for 359 diseases and injuries and healthy life expectancy (HALE) for 195 countries and territories, 1990–2017: a systematic analysis for the Global Burden of Disease Study 2017. *The Lancet*, *392*(10159), 1859–1922. https://doi.org/10.1016/S0140-6736(18)32335-3

Metzger, W. G., & Mordmüller, B. (2014). Loa loa—does it deserve to be neglected? *The Lancet Infectious Diseases*, *14*(4), 353–357. https://doi.org/10.1016/S1473-3099(13)70263-9

Moser, W., Coulibaly, J. T., Ali, S. M., Ame, S. M., Amour, A. K., Yapi, R. B., Albonico, M., Puchkov, M., Huwyler, J., Hattendorf, J., & Keiser, J. (2017). Efficacy and safety of tribendimidine, tribendimidine plus ivermectin, tribendimidine plus oxantel pamoate, and albendazole plus oxantel pamoate against hookworm and concomitant soil-transmitted helminth infections in Tanzania and Côte d'Ivoire: a randomised, controlled, single-blinded, non-inferiority trial. *The Lancet Infectious Diseases*, *17*(11), 1162–1171. https://doi.org/10.1016/S1473-3099(17)30487-5

Osei-Atweneboana, M. Y., Awadzi, K., Attah, S. K., Boakye, D. A., Gyapong, J. O., & Prichard, R. K. (2011). Phenotypic Evidence of Emerging Ivermectin Resistance in Onchocerca volvulus. *PLoS Neglected Tropical Diseases*, *5*(3), e998. https://doi.org/10.1371/journal.pntd.0000998

Pionnier, N. P., Sjoberg, H., Chunda, V. C., Fombad, F. F., Chounna, P. W., Njouendou, A. J., Metuge, H. M., Ndzeshang, B. L., Gandjui, N. V., Akumtoh, D. N., Tayong, D. B., Taylor, M. J., Wanji, S., & Turner, J. D. (2019). Mouse models of Loa loa. *Nature Communications*, *10*(1), 1429. https://doi.org/10.1038/s41467-019-09442-0

Rizopoulos D. (2023). *GLMMadaptive: Generalized Linear Mixed Models using Adaptive Gaussian Quadrature*. https://drizopoulos.github.io/GLMMadaptive/

Schneeberger, P. H. H., Gueuning, M., Welsche, S., Hürlimann, E., Dommann, J., Häberli, C., Frey, J. E., Sayasone, S., & Keiser, J. (2022). Different gut microbial communities correlate with efficacy of albendazole-ivermectin against soil-transmitted helminthiases. *Nature Communications*, *13*(1), 1063. https://doi.org/10.1038/s41467-022-28658-1

Simonsen, P. E., Onapa, A. W., & Asio, S. M. (2011). Mansonella perstans filariasis in Africa. *Acta Tropica*, *120*, S109–S120. https://doi.org/10.1016/j.actatropica.2010.01.014

U.S. Department of Health and Human Services Food and Drug Administration. (2009). *Guidance for Industry Drug-Induced Liver Injury: Premarketing Clinical Evaluation*. https://www.fda.gov/media/116737/download?attachment

U.S. National Institutes of Health National Cancer Institute. (2017). *Common Terminology Criteria for Adverse Events (CTCAE) Version 5.0*. https://ctep.cancer.gov/protocoldevelopment/electronic_applications/docs/CTCAE_v5_Quick_Reference_8.5x11.pdf

Verbeeck, J., Deltuvaite-Thomas, V., Berckmoes, B., Burzykowski, T., Aerts, M., Thas, O., Buyse, M., & Molenberghs, G. (2021). Unbiasedness and efficiency of non-parametric and UMVUE estimators of the probabilistic index and related statistics. *Statistical Methods in Medical Research*, *30*(3), 747–768. https://doi.org/10.1177/0962280220966629

Verver, S., Walker, M., Kim, Y. E., Fobi, G., Tekle, A. H., Zouré, H. G. M., Wanji, S., Boakye, D. A., Kuesel, A. C., de Vlas, S. J., Boussinesq, M., Basáñez, M.-G., & Stolk, W. A. (2018). How Can Onchocerciasis Elimination in Africa Be Accelerated? Modeling the Impact of Increased Ivermectin Treatment Frequency and Complementary Vector Control. *Clinical Infectious Diseases*, *66*(suppl_4), S267–S274. https://doi.org/10.1093/cid/cix1137

Vinkeles Melchers, N. V. S., Coffeng, L. E., Boussinesq, M., Pedrique, B., Pion, S. D. S., Tekle, A. H., Zouré, H. G. M., Wanji, S., Remme, J. H., & Stolk, W. A. (2020). Projected Number of People With Onchocerciasis–Loiasis Coinfection in Africa, 1995 to 2025. *Clinical Infectious Diseases*, *70*(11), 2281–2289. https://doi.org/10.1093/cid/ciz647

Walker, M., Stolk, W. A., Dixon, M. A., Bottomley, C., Diawara, L., Traoré, M. O., de Vlas, S. J., & Basáñez, M.-G. (2017). Modelling the elimination of river blindness using long-term epidemiological and programmatic data from Mali and Senegal. *Epidemics*, *18*, 4–15. https://doi.org/10.1016/j.epidem.2017.02.005

Welsche, S., Mrimi, E. C., Hattendorf, J., Hürlimann, E., Ali, S. M., & Keiser, J. (2023). Efficacy and safety of moxidectin and albendazole compared with ivermectin and albendazole coadministration in adolescents infected with Trichuris trichiura in Tanzania: an open-label, non-inferiority, randomised, controlled, phase 2/3 trial. *The Lancet. Infectious Diseases*, *23*(3), 331–340. https://doi.org/10.1016/S1473-3099(22)00589-8

Wolstenholme, A. J., Fairweather, I., Prichard, R., von Samson-Himmelstjerna, G., & Sangster, N. C. (2004). Drug resistance in veterinary helminths. *Trends in Parasitology*, *20*(10), 469–476. https://doi.org/10.1016/j.pt.2004.07.010

World Health Organization. (2017). Progress Report on the Elimination of Human Onchocerciasis. *Weekly Epidemiological Record*, *45*, 681–700.

Investigator Signature Page

I have read this protocol and agree that it contains all information necessary to carry out the trial. I will conduct the trial as described herein.

I will provide copies of the protocol and all pertinent information to all individuals responsible to me who assist in the conduct of the trial. I will discuss this material with them to ensure they are fully informed regarding the Investigational Product and the conduct of the trial.

I will use only the Informed Consent Form approved by the Sponsor or its representative, and the ethics committee(s) and (where applicable) the regulatory authorities and will fulfil all responsibilities for submitting pertinent information to the ethics committee(s) responsible for the trial if required by national law.

I agree that the Sponsor or its representatives, and respective ethics committee(s) and regulatory authorities shall have access to any source documents from which case report form information may have been generated.

I agree that the trial will be conducted in accordance with all applicable laws and regulations, including, but not limited to, the International Conference on Harmonisation (ICH) Guideline for Good Clinical Practice (GCP), the ethical principles that have their origins in the Declaration of Helsinki, and applicable privacy laws.

Nothing in this document is intended to limit the authority of a physician to provide emergency medical care under applicable regulations.

Name of Co-Principal Investigator	Said Mohammed Ali
Affiliation:	Public Health Laboratory Ivo de Carneri
Date: 19-Nov-2024 | 07:06:03 CET	Signature: DocuSigned by: [signature] 02C1CF019D1B4EA...
Name of Study Physician:	Dr. Ibrahim Said Mohammed
Affiliation:	Ministry of Health Pemba
Date: 18-Nov-2024 | 20:08:30 CET	Signature: Signed by: [signature] 03049BB2DF354F5...
Name of Co-Principal Investigator	Annina Schnoz
Affiliation:	Swiss Tropical and Public Health Institute
Date: 20-Nov-2024 | 08:04:08 CET	Signature: DocuSigned by: Annina Schnoz 64CE9CBC485F4F2...

Signed copies of this signature page are stored in the Sponsor's trial master file and in the Investigator Site File at the investigational centre.

Investigator Signature Page

I have read Appendix 5 for Tanzania of this protocol and agree that it contains all information necessary to carry out the trial. I will conduct the trial as described herein.

Nothing in Appendix 5 for Tanzania of this protocol is intended to limit the authority of a physician to provide emergency medical care under applicable regulations.

Name of Sponsor Representative: Jennifer Keiser

Affiliation: Swiss Tropical and Public Health Institute

Date: 14-Apr-2025 | 08:51:30 MESZ Signature: DocuSigned by: Jennifer Keiser F8F6BD544E39400...

Name of Co-Prinicpal Investigator: Said Mohammed Ali

Affiliation: Public Health Laboratory Ivo de Carneri

Date: 15-Apr-2025 | 10:22:01 CEST Signature: DocuSigned by: 02C1CF019D1B4EA...

Name of Study Physician: Dr. Ibrahim Said Mohammed

Affiliation: Ministry of Health Pemba

Date: 14-Apr-2025 | 13:15:21 CEST Signature: Signed by: 62757F27FF414FE...

Name of Co-Principal Investigator: Annina Schnoz

Affiliation: Swiss Tropical and Public Health Institute

Date: 14-Apr-2025 | 08:55:43 CEST Signature: DocuSigned by: Annina Schnoz 64CE9CBC485F4F2...

Signed copies of this signature page are stored in the Sponsor's trial master file and in the Investigator Site File at the investigational centre.
