# Supplementary material for: Efficacy and safety of oxfendazole in adults with Trichuris spp. infection in Pemba Island, Tanzania: a double-blind, phase 2a, randomised controlled trial
Source: eClinicalMedicine. 2026 Oct 26;101:104273. doi: 10.1016/j.eclinm.2026.104273 (PMC13734800; doi:10.1016/j.eclinm.2026.104273)
Supplement: Supplementary file 2 [file mmc1.docx]

**Supplementary Tables and Figures**

**Table of Contents**

[Summary in Swahili.	2](#_Toc240290999)

[*Table S1:* Prohibited Concomitant Therapy.	3](#_Toc240291000)

[*Table S2*: Egg count by treatment arm with multiple imputations of missing values, ITT population.	4](#_Toc240291001)

[*Table S3*: A baseline adjusted sensitivity analysis of the primary endpoint.	5](#_Toc240291002)

[*Table S4: Trichuris* spp*.* EPG at day 18 by treatment arm, mITT population.	6](#_Toc240291003)

[*Table S5*: Results of repeated analysis.	7](#_Toc240291004)

[*Table S6:* *A. lumbricoides* and hookworm cure rates by treatment arm, mITT population.	8](#_Toc240291005)

[*Figure S1:* *A. lumbricoides* and hookworm individual trajectories of egg count by treatment arm, mITT population.	8](#_Toc240291006)

[*Table S7:* *A. lumbricoides* and hookworm egg reduction rates.	9](#_Toc240291007)

[*Table S8:* *Trichuris* spp.*,* *A. lumbricoides* and hookworm egg reduction rates by baseline infection intensity.	10](#_Toc240291008)

[*Table S9:* Adverse events including duration and relatedness.	11](#_Toc240291009)

[*Table S10:* Vital Signs.	13](#_Toc240291010)

[*Table S11:* Physical Examination.	16](#_Toc240291011)

[*Table S12:* Neurological Examination.	18](#_Toc240291012)

[*Table S13:* Clinical Chemistry.	19](#_Toc240291013)

[*Table S14:* Haematology.	22](#_Toc240291014)

[*Table S15:* Urinalysis.	25](#_Toc240291015)

# Summary in Swahili.

The following translations in Swahili were submitted by the authors and we reproduce them as supplied. They have not been peer reviewed. Our editorial processes have only been applied to the original abstract in English, which should serve as reference for this manuscript.

**Muhtasari**

**Usuli:** Ugonjwa wa minyoo mjeledi (trikuriasisi), unaosababishwa na minyoo *Trichuris trichiura* na *T. incognita*, bado ni tatizo kubwa la afya ya jamii. Dawa za kundi la benzimidazole, kama vile albendazole na mebendazole, zinazotumika sana kwa matibabu yake zinaonyesha ufanisi mdogo, jambo linaloashiria kuwa matibabu bora zaidi yanahitajika. Dawa mojawapo yenye matumaini ni oxfendazole, dawa ya minyoo yenye ufanisi mkubwa ambayo hutumika sana katika tiba ya mifugo. Ndani ya mradi wa eWHORM, mradi wa kimataifa wa utafiti wa matibabu unaolenga kutokomeza magonjwa ya minyoo yaliyosahaulika, oxfendazole inafanyiwa tafiti ili kubaini ufanisi wake katika kukabiliana na magonjwa ya minyoo, ikiwemo trikuriasisi.

**Mbinu:** Katika jaribio hili la awamu ya 2a, lililofanywa kwa njia ya upofu mara mbili, ugawaji wa nasibu na kulinganisha na dawa bandia (placebo), kati ya tarehe 29 Aprili na 16 Julai 2025 katika Kisiwa cha Pemba, Tanzania, usalama na ufanisi wa oxfendazole dhidi ya maambukizi ya *Trichuris* spp. ulitathminiwa miongoni mwa watu wazima wenye umri wa miaka 18 hadi 65. Jumla ya washiriki 127 waligawanywa kwa nasibu katika uwiano wa 1:1:1:1 na kupokea mojawapo ya matibabu yafuatayo: dawa bandia kwa siku tatu mfululizo; miligramu 400 za oxfendazole siku ya kwanza ikifuatiwa na dawa bandia kwa siku mbili; miligramu 800 za oxfendazole siku ya kwanza ikifuatiwa na dawa bandia kwa siku mbili; au miligramu 800 za oxfendazole kwa siku tatu mfululizo. Ufanisi ulitathminiwa kwa kutumia kiwango cha kupona, kilichofafanuliwa kama uwiano wa washiriki waliokuwa na maambukizi ya *Trichuris* spp. mwanzoni mwa utafiti na waliothibitika kutokuwa na maambukizi baada ya matibabu katika siku ya 18. Matukio mabaya ya kiafya yalirekodiwa saa 3, saa 24 na siku ya 18 baada ya matibabu. Jaribio hili lilisajiliwa katika Daftari la Majaribio ya Kliniki la Pan African (PACTR202412611774752).

**Matokeo:** Kati ya tarehe 29 Aprili na 21 Mei 2025, washiriki 229 walifanyiwa uchunguzi wa awali wa maambukizi ya *Trichuris* spp., ambapo 127 walitimiza vigezo vya ushiriki na kupatiwa matibabu. Miongoni mwao, washiriki 31 walipokea dozi ya miligramu 800 za oxfendazole kwa siku tatu mfululizo (3x800 mg), 34 walipokea dozi moja ya miligramu 800 (1x800 mg), 30 walipokea dozi moja ya miligramu 400 (1x400 mg), na 32 walipokea dawa bandia (placebo). Kiwango cha kupona kilikuwa 75·9% (95% CI: 57·9 – 87·8) kwa kundi lililopokea dozi ya miligramu 800 kwa siku tatu mfululizo. Viwango vya kupona kwa dozi moja ya oxfendazole vilikuwa vya chini, yaani 31·0% (95% CI: 17·3 – 49·2) kwa dozi ya miligramu 400 na 35·3% (95% CI: 21·5 – 52·1) kwa dozi ya miligramu 800. Kundi la placebo lilikuwa na kiwango cha kupona cha 20·0% (95% CI: 9·5 – 37·3). Dozi zote za oxfendazole zilistahimilika vyema na zilionyesha idadi ya matukio mabaya ya kiafya iliyofanana na ile ya kundi la placebo. Athari mbaya iliyoripotiwa mara nyingi zaidi ilikuwa maumivu ya tumbo.

**Tafsiri:** Dozi moja ya oxfendazole haikuonyesha faida ya ziada ikilinganishwa na viwango vya kupona vilivyoripotiwa katika tafiti za awali za albendazole na mebendazole dhidi ya maambukizi ya Trichuris spp. Hata hivyo, kutokana na ufanisi mkubwa ulioonekana kwa matumizi ya dozi nyingi, oxfendazole inaweza kuwa na nafasi katika mikakati ya matibabu inayolenga makundi mahususi ya wagonjwa. Aidha, matumizi ya dozi nyingi za oxfendazole yanaweza kutoa manufaa ya ziada kwa afya ya jamii katika kudhibiti maambukizi ya *Trichuris* spp., hasa inapotekelezwa sambamba na matibabu ya maambukizi mengine ya minyoo yanayohitaji dozi za siku kadhaa katika maeneo ambako maambukizi hayo hutokea kwa pamoja.

# *Table S1:* Prohibited Concomitant Therapy.

Throughout the entire clinical trial, the use of medication that may have an impact on the clinical trial objectives was prohibited.

a) Anthelmintic treatment: ivermectin, moxidectin, diethylcarbamazine, albendazole, mebendazole and doxycycline.
b) Drugs with known hepatoxicity or with myelosuppressive activity.
c) Drugs prohibited while on clinical trial up to end of exposure (Day 18), CYP1A2 substrates (Gelbe Liste, 2019).

# *Table S2*: Egg count by treatment arm with multiple imputations of missing values, ITT population.

A zero-inflated Poisson model was used for multiple imputation because egg count data are non-negative integer counts and results of available data showed a large excess of zero values. The zero-inflated Poisson model represents this data-generating structure by modelling both the probability of a zero count and the mean of the positive count observations. The multiple imputation analysis assumed that data were missing at random, conditional on the variables included in the imputation model.

# *Table S3*: A baseline adjusted sensitivity analysis of the primary endpoint.

| **Arm** | **Adjusted mean difference in log-transformed egg counts** |
| --- | --- |
| 1x400mg OXF | *−1.29 (95% CI −2.42 to −0.17; adjusted one-sided p=0.0251)* |
| 1x800mg OXF | *−1.19 (95% CI −2.35 to −0.04; adjusted one-sided p=0.0251)* |
| 3x800mg OXF | *−3.95 (95% CI −5.01 to −2.82; adjusted one-sided p=<0.001)* |

Because the egg counts were highly right-skewed and included zeros, follow-up counts were transformed as log(egg count+1). We fitted generalized least-squares (GLS) models including treatment and baseline log(egg count + 1) count as covariates. To account for heterogeneity of variances between treatment groups, group-specific residual variances were allowed in the model. We note, however, that the follow-up distribution contained a substantial proportion of zeros, reaching approximately 75% in in the 3x800 treatment group. Therefore, this analysis should be interpreted as a sensitivity analysis rather than as a distributionally optimal model for the endpoint.

We report the baseline-adjusted treatment effects with two-sided 95% confidence intervals and the corresponding one-sided Bonferroni Holm adjusted p-values, consistent with the direction of the prespecified primary hypothesis. The adjusted analyses were consistent with the direction of the effects observed in the primary Wilcoxon analysis and provided stronger statistical evidence for all treatment comparisons. The prespecified Wilcoxon analysis remains the primary analysis, whereas the baseline-adjusted GLS analysis is reported as a sensitivity analysis.

# *Table S4: Trichuris* spp*.* EPG at day 18 by treatment arm, mITT population.

The probabilistic index is reported for each active dose in comparison to placebo group. It is the probability that a randomly chosen participant from the active dose group has a smaller EPG at day 18 than a randomly chosen participant from the Placebo group. A value of 0.5 means that there is no difference between the two groups, a higher value means that participants in the active dose groups tend to have smaller EPG at day 18 than participants in the placebo group.

# *Table S5*: Results of repeated analysis.

a) The Wilcoxon was chosen for the comparison of egg counts because the count distributions were strongly right-skewed, included zero values. The anticipated sample size was 40 per group. The tests were one-sided because the treatment effect of interest was prespecified in the direction of lower egg counts under treatment.

The analysis was repeated for the primary endpoint with an exact test and obtained very similar results.

| **Arm** | **p-values and CI (95%, two-sided) for the  probabilistic index** |
| --- | --- |
| 1x400mg OXF | p=0.174 (0.422 – 0.700) |
| 1x800mg OXF | p=0.132 (0.465 – 0.749) |
| 3x800mg OXF | p=<0.001 (0.591 – 1.000) |

The p-values differed only in the 3^rd^ decimal place the confidence intervals for the probabilistic index were slightly wider.

b) The chi-square approximation may be inadequate for comparisons of cure rates when cell counts are small, particularly in the moderate/high-intensity subgroup. For trichuriasis the analysis was therefore recalculated by Fisher’s exact test for these comparisons.

| **Arm** | **p-values for Fisher’s exact test** | **p-values for chi-square test** |
| --- | --- | --- |
| 1x400mg OXF | p=0.28 | p=0.17 |
| 1x800mg OXF | p=0.28 | p=0.17 |
| 3x800mg OXF | p=<0.001 | p=<0.001 |

The corresponding one-sided tests changed only slightly and the 3x800 group remained statistically significant. For *A. lumbricoides* and hookworm cure rates were analysed with Fisher’s exact test due to the small sample sizes in the secondary  analyses.

# *Table S6:* *A. lumbricoides* and hookworm cure rates by treatment arm, mITT population.

N=number; CI=confidence interval.

a) Cure rate by treatment arm - *A. lumbricoides.*
b) Cure rate by treatment arm – Hookworm.

# *Figure S1:* *A. lumbricoides* and hookworm individual trajectories of egg count by treatment arm, mITT population.

Log egg counts per gram of stool displayed for every participant at baseline (Day 0) and follow-up (Day 18), by treatment arm. Every point represents a single participant, paired values (same participant at Day 0 and Day 18) are connected with a line. The boxplots represent the median, 25% and 75% percentile (lower and upper limit respectively).

a)*A. lumbricoides*.
b) Hookworm.

# *Table S7:* *A. lumbricoides* and hookworm egg reduction rates.

a) Arithmetic egg reduction rates by parasite and treatment group, mITT population is presented for each treatment arm.

b) Geometric egg reduction rates by parasite and treatment group, mITT population is presented for each treatment arm.

# *Table S8:* *Trichuris* spp.*,* *A. lumbricoides* and hookworm egg reduction rates by baseline infection intensity.

a) Arithmetic egg reduction rates by parasite, baseline infection intensity and treatment group, mITT population is presented for each treatment arm.

b) Geometric egg reduction rates by parasite, baseline infection intensity and treatment group, mITT population is presented for each treatment arm

# *Table S9:* Adverse events including duration and relatedness.

For each adverse event, the severity, relatedness to study treatment, and duration (days) are provided in parentheses. OXF = oxfendazole.

|  | Placebo  (n=32) | 1x400mg OXF (n=30) | 1x800mg OXF (n=34) | 3x800mg OXF (n=31) |
| --- | --- | --- | --- | --- |
| Adverse events 1^st^ treatment day | | | | |
| Abdominal discomfort/pain (mild, RELATED, 1 day) | 2 (6.2%) | 0 | 2 (5.9%) | 0 |
| Abdominal discomfort/pain (mild, RELATED, 2 days) | 0 | 0 | 0 | 2 (6.5%) |
| Dizziness (mild, RELATED, 1 day) | 2 (6.2%) | 0 | 3 (8.8%) | 0 |
| Headache (mild, RELATED, 1 day) | 0 | 0 | 1 (2.9%) | 1 (3.2%) |
| Nausea (mild, RELATED, 1 day) | 0 | 0 | 1 (2.9%) | 0 |
| Adverse events 2^nd^ treatment day | | | | |
| Abdominal discomfort/pain (mild, RELATED, 1 day) | 2 (6.2%) | 0 | 0 | 2 (6.5%) |
| Headache (mild, RELATED, 1 day) | 1 (3.1%) | 1 (3.3%) | 0 | 0 |
| Adverse events 3^rd^ treatment day | | | | |
| Abdominal discomfort/pain (mild, RELATED, 17 days) | 0 | 1 (3.3%) | 0 | 0 |
| Chest pain (mild, NOT RELATED, 1 day) | 0 | 0 | 0 | 1 (3.2%) |
| Diarrhoea (mild, NOT RELATED, 1 day) | 0 | 0 | 1 (2.9%) | 0 |
| Diarrhoea (mild, RELATED, 1 day) | 1 (3.1%) | 0 | 0 | 0 |
| Dizziness (mild, RELATED, 1 day) | 0 | 0 | 0 | 1 (3.2%) |
| Headache (mild, RELATED, 1 day) | 0 | 1 (3.3%) | 0 | 0 |
| Nausea (mild, RELATED, 1 day) | 0 | 2 (6.7%) | 0 | 0 |
| Nausea (mild, RELATED, 16 days) | 0 | 0 | 0 | 1 (3.2%) |
| Palpitations (mild, NOT RELATED, 1 day) | 0 | 0 | 1 (2.9%) | 0 |
| Panic attack (moderate, NOT RELATED, 1 day) | 0 | 0 | 1 (2.9%) | 0 |
| Adverse events day 4 | | | | |
| Headache (mild, RELATED, 16 days) | 1 (3.1%) | 0 | 0 | 0 |
| Adverse events with unknown start date after treatment | | | | |
| Abdominal discomfort/pain (NA, NOT APPLICABLE, Unknown duration) | 1 (3.1%) | 0 | 0 | 0 |
| Influenza (mild, NOT APPLICABLE, Unknown duration) | 0 | 0 | 1 (2.9%) | 0 |
| Pain in extremity (mild, NOT RELATED, Unknown duration) | 0 | 0 | 0 | 1 (3.2%) |
| Pollakiuria (mild, NOT RELATED, Unknown duration) | 0 | 0 | 0 | 1 (3.2%) |
| Pyrexia (NA, NOT APPLICABLE, Unknown duration) | 0 | 0 | 1 (2.9%) | 0 |
| Abdominal discomfort/pain (NA, NOT APPLICABLE, Unknown duration) | 1 (3.1%) | 0 | 0 | 0 |
| Total adverse events | 10 | 5 | 12 | 10 |

# *Table S10:* Vital Signs.

a) Descriptive statistics of vital signs (baseline), safety population.

b) Descriptive statistics of vital signs (day 18), safety population.

c) Change from baseline to day 18 in vital signs, safety population.

# *Table S11:* Physical Examination.

a) Summary of complete physical examination findings (baseline) by treatment arm, safety population.

b) Summary of complete physical examination findings (day 18) by treatment arm, safety population. Findings at day 18 revealed no new abnormalities compared with baseline.

# *Table S12:* Neurological Examination.

a) Summary of neurological examination findings (baseline) by treatment arm, safety population.
b) Summary of neurological examination findings (day 18) by treatment arm, safety population. Findings at day 18 revealed no new abnormalities compared with baseline*.*

# *Table S13:* Clinical Chemistry.

a) Elevated liver enzyme values at day 18 compared with baseline and day 47.

|  | Baseline | Day 18 | Day 47 | Arm |
| --- | --- | --- | --- | --- |
| Participant 1: Alanine Aminotransferase (U/L) | 27 | 64 | 22 | 1x400 mg OXF |
| Participant 2: Alanine Aminotransferase (U/L) | 33 | 82 | 23 | 3x800 mg OXF |
| Participant 2: Aspartate Aminotransferase (U/L) | 30 | 67 | 22 | 3x800 mg OXF |
| Participant 3: Alanine Aminotransferase (U/L) | 18 | 50 | 22 | 3x800 mg OXF |
| Participant 3: Aspartate Aminotransferase (U/L) | 16 | 67 | 36 | 3x800 mg OXF |

b) Chemistry - descriptive summary (baseline), safety population.

* According to the note to file OXF-01 protocol v2.0 from December 10, 2025, the measured urea values were converted to blood urea nitrogen (BUN) using the following formula: BUN = UREA / 2.1428.

c) Chemistry - descriptive summary (day 18), safety population.

# *Table S14:* Haematology.

a)
Haematology - descriptive summary (baseline), safety population.

b) Haematology - descriptive summary (day 18), safety population.

# *Table S15:* Urinalysis.

a) Urinalysis (continuous parameters: specific gravity and pH) - descriptive summary (baseline), safety population.
b) Urinalysis (continuous parameters: specific gravity and pH) - descriptive summary (day 18), safety population.

c) Urinalysis (ordinal parameters) - contingency table (baseline), safety population.

d) Urinalysis (ordinal parameters) - contingency table (day 18), safety population.
